# Supplementary figures and images for: Derivation of Russian-specific reference intervals for complete blood count, iron markers and related vitamins
Source: PLoS One. 2024 Sep 30;19(9):e0304020. doi: 10.1371/journal.pone.0304020 (PMC11441669; doi:10.1371/journal.pone.0304020)

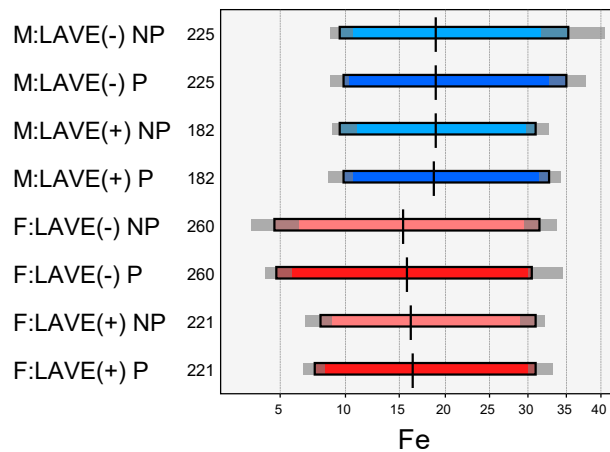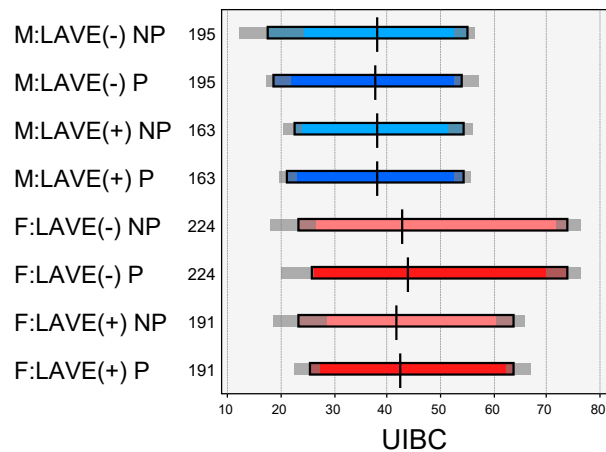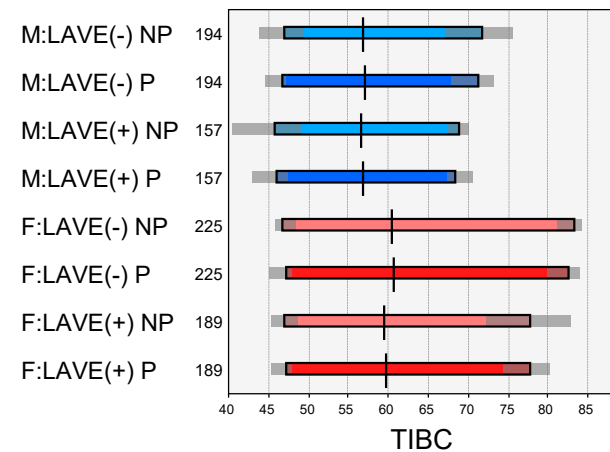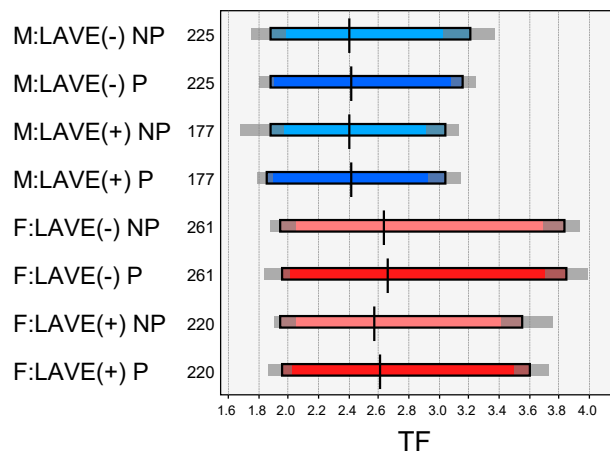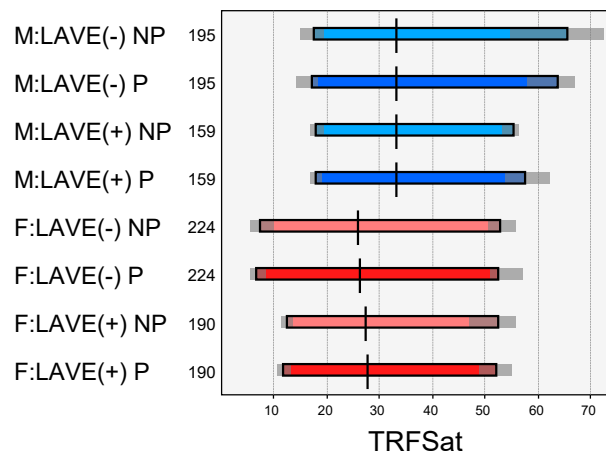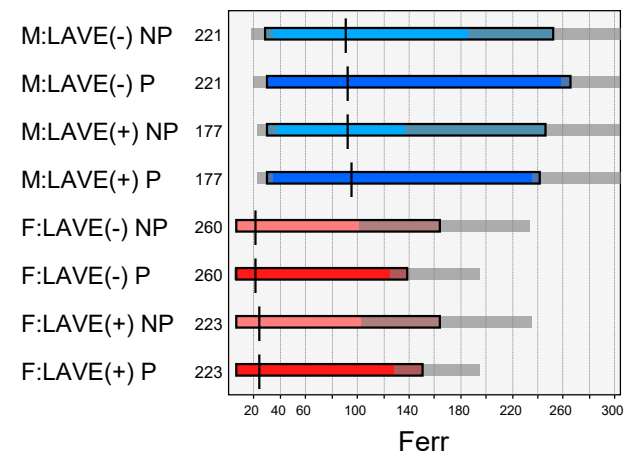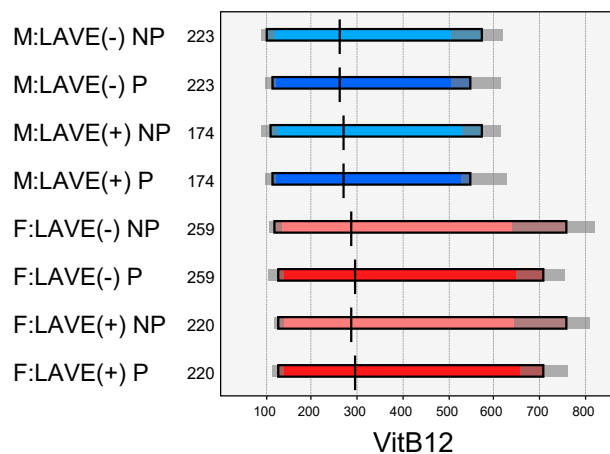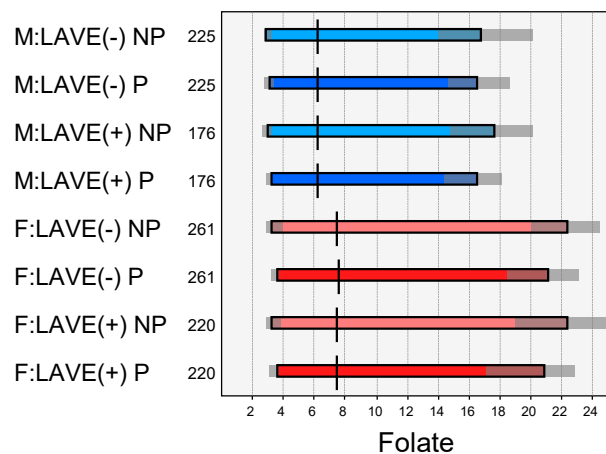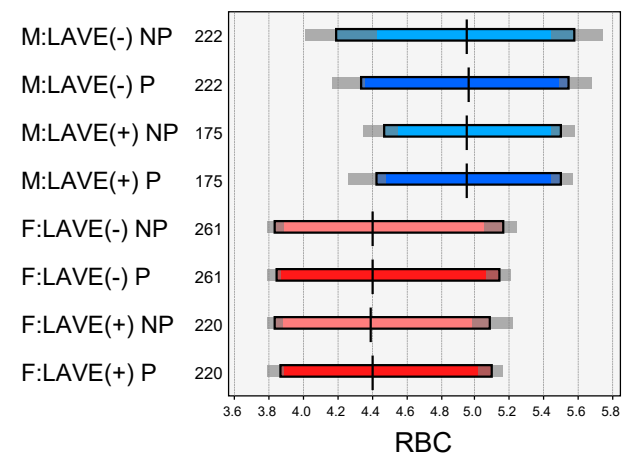

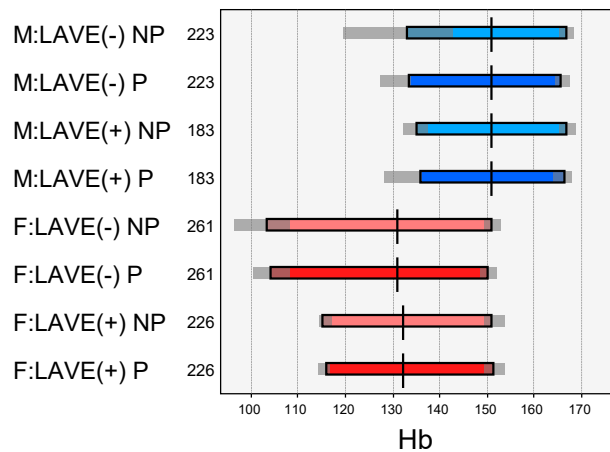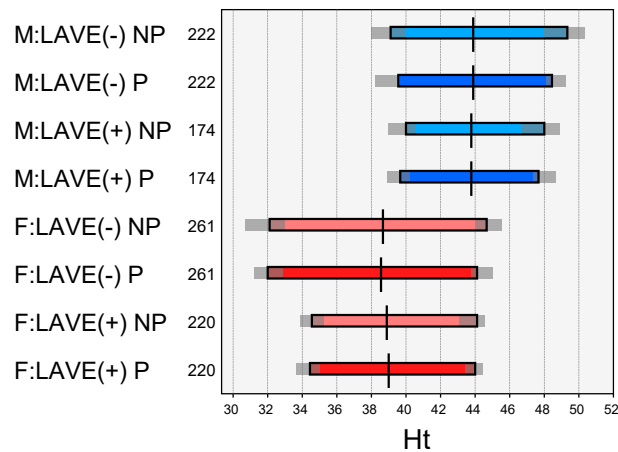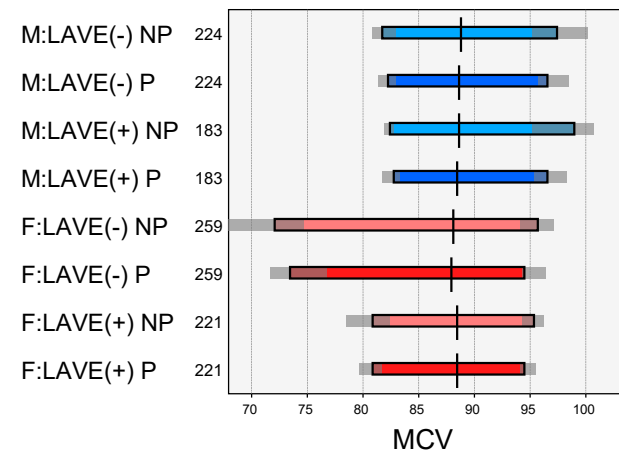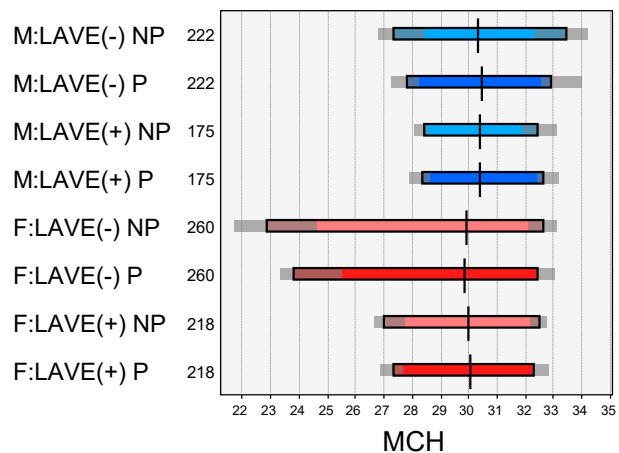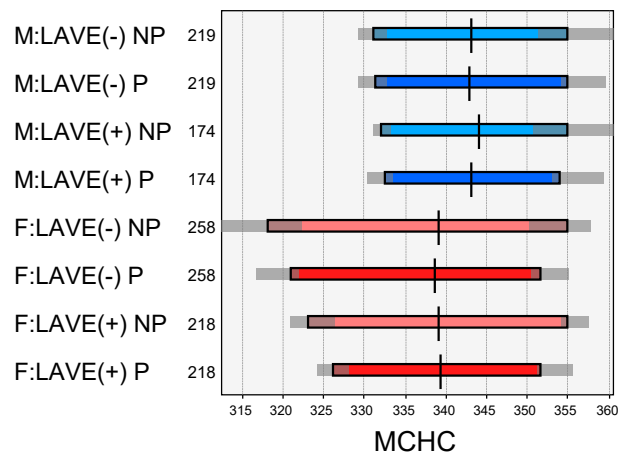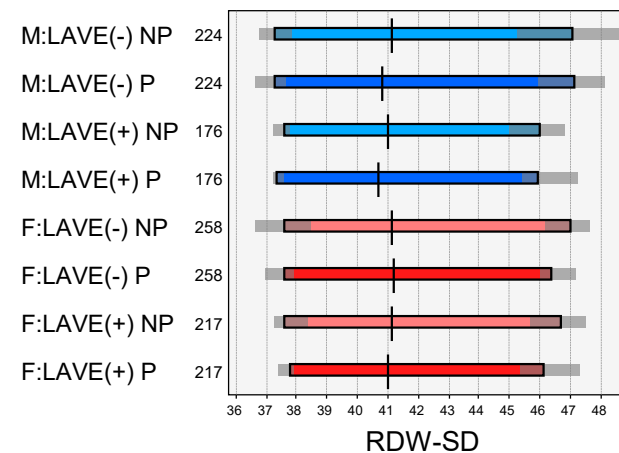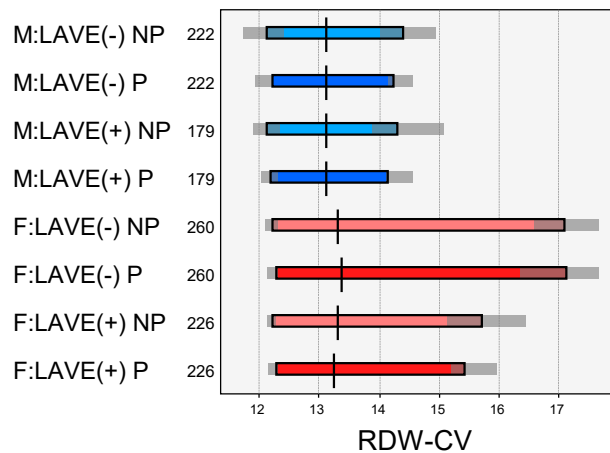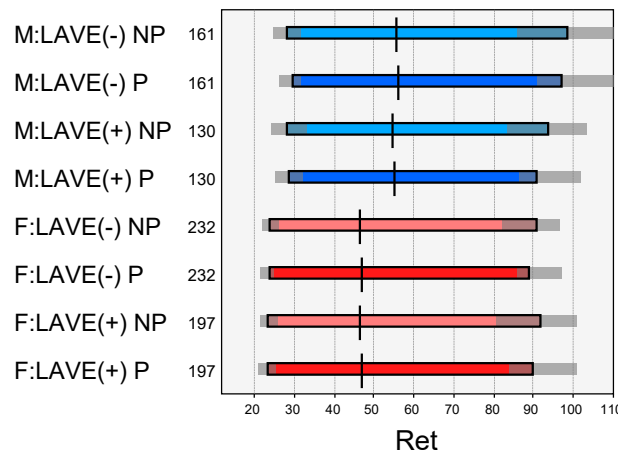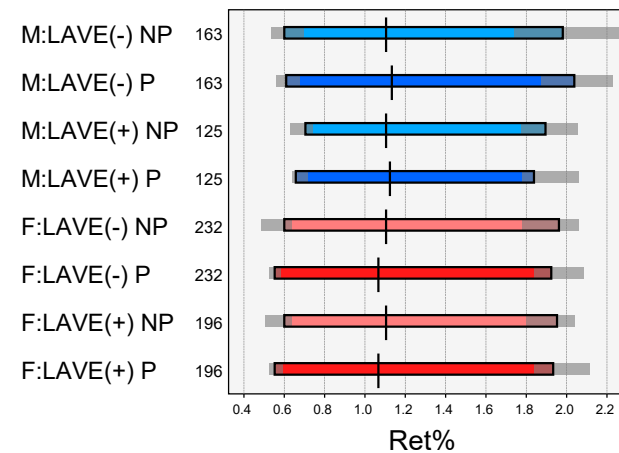

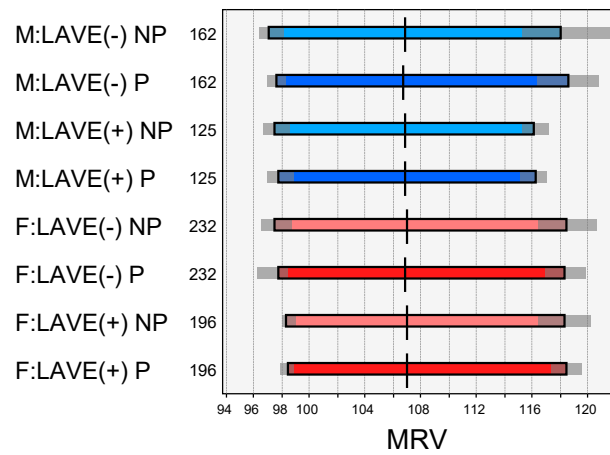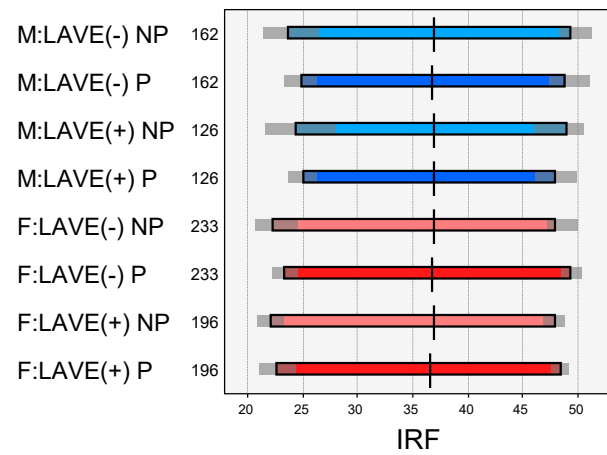

Supplement: S1 Fig — The RIs were derived in four ways: by parametric (P) or non-parametric (NP) method with or without application of latent abnormal values exclusion method (LAVE) method. Each horizontal bar represents the RI, and the vertical line in the center corresponds to the midpoint. The shades on both ends of the bar represent 90% CI for the limits of the RI predicted by the bootstrap method. The RIs derived for twenty parameters related to red blood cells (RBC) and anemia associated markers (AAM) for males (M: blue) and females (F: red) are shown in this figure. (PDF) [file pone.0304020.s001.pdf]
