# Supplementary material for: Derivation of Russian-specific reference intervals for complete blood count, iron markers and related vitamins
Source: PLoS One. 2024 Sep 30;19(9):e0304020. doi: 10.1371/journal.pone.0304020 (PMC11441669; doi:10.1371/journal.pone.0304020)
Supplement: Attachment — Submitted filename: PONE-D-23-22704_Reviewed.pdf [file pone.0304020.s004.pdf]

PLOS ONE

Derivation of Russian-specific reference intervals for complete blood count, iron markers and related vitamins

--Manuscript Draft--

**Manuscript Number:**	PONE-D-23-22704
**Article Type:**	Research Article
**Full Title:**	Derivation of Russian-specific reference intervals for complete blood count, iron markers and related vitamins
**Short Title:**	Hematology reference intervals for Russian Population
**Corresponding Author:**	Kiyoshi Ichihara, M.D. & Ph.D<br>Yamaguchi Daigaku Igakubu Daigakuin Igakukei Kenkyuka<br>Ube, Yamaguchi JAPAN
**Keywords:**	reference value (RV), reference interval (RI), sources of variation; latent abnormal values exclusion method (LAVE); nested ANOVA; standard deviation ratio (SDR); multiple regression analysis (MRA); latent anemia
**Abstract:**	Objectives<br>This study was aimed to establish reference intervals (RIs) for Russian adults for hematological parameters including related iron markers and vitamins. Sources of variation of reference values (RVs) and needs for secondary exclusion were explored for proper derivations of RIs.<br>Methods<br>Following the harmonized protocol of the IFCC Committee on Reference Intervals and Decision Limits (C-RIDL), 506 healthy Russians (age 18−80; 46% male) were recruited. Complete blood counts (CBC) and leukocyte differentials, iron markers, vitamin B12, and folate were measured by Beckman Coulter's analyzers. Sources of variation were analyzed by multiple regression analysis, and ANOVAs, and the need for partitioning RVs was decided accordingly. Two schemes of excluding latent anemia were compared: (1) latent abnormal values exclusion method (LAVE) by specifying 5−7 reference tests among CBC parameters, or (2) explicit exclusion of individuals with either ferritin or iron below the respective lower limit. RIs were determined by the parametric method using two-parameter Box-Cox power-transformation formula.<br>Results<br>Gender-specific RIs were required for most analytes, while age-specific RIs were set only to ferritin in females. BMI-related increase in RVs was observed prominently for reticulocyte parameters, for which we chose to exclude individuals with BMI>28 kg/m2 when establishing the RIs. The LAVE method was found to be more effective in excluding individuals with latent anemia, than exclusion based on low ferritin and/or iron values. International comparison revealed that Russian RIs featured a lower side shift of platelet counts. As in African countries, Russian RIs for total leukocyte and neutrophil counts were lower compared to most of other countries.<br>Conclusion<br>RIs for the Russian population for 34 hematological and related parameters were established using up-to-date methods proposed by C-RIDL. Reducing influences of latent anemia and obesity on RIs was crucial for erythrocyte parameters. Low levels of Russian RIs observed for platelet and neutrophil count need further investigation.
**Order of Authors:**	Anna Ruzhanskaya, PhD
	Kiyoshi Ichihara, Professor, MD, PhD
	Elena Sukhacheva, PhD
	Irina Skibo, MD, PhD
	Nina Vybornova
	Dmitry Butlitski, MD, PhD
	Anton Vasiliev
	Galina Agarkova

Powered by Editorial Manager® and ProduXion Manager® from Aries Systems Corporation

	Ekaterina Vilenskaya
	Vladimir Emanuel, Professor, MD, PhD
	Svetlana Lugovskaya, Professor, MD, PhD
**Additional Information:**	
**Question**	**Response**
**Financial Disclosure**<br><br>Enter a financial disclosure statement that describes the sources of funding for the work included in this submission. Review the submission guidelines for detailed requirements. View published research articles from *PLOS ONE* for specific examples.<br><br>This statement is required for submission and **will appear in the published article** if the submission is accepted. Please make sure it is accurate.<br><br>**Unfunded studies**<br>Enter: *The author(s) received no specific funding for this work.*<br><br>**Funded studies**<br>Enter a statement with the following details:<br>• Initials of the authors who received each award<br>• Grant numbers awarded to each author<br>• The full name of each funder<br>• URL of each funder website<br>• Did the sponsors or funders play any role in the study design, data collection and analysis, decision to publish, or preparation of the manuscript?<br>• **NO** - Include this sentence at the end of your statement: *The funders had no role in study design, data collection and analysis, decision to publish, or preparation of the manuscript.*<br>• **YES** - Specify the role(s) played.<br><br>* typeset	Beckman Coulter, LLC (Moscow, Russia; https://www.beckmancoulter.com/ru) supported the study of the assay reagents and Helix Laboratories Services (Saint-Petersburg, Russia; https://helix.ru/) assisted in the recruitment of volunteers, sample preparations, and provision of sampling equipment.<br><br>The funders provided support in the form of salaries for authors (AR, ES, DB, GA, EV, IS, NV, AV), but did not take any role in the study design, data collection and analysis, decision to publish, or preparation of the manuscript. The specific roles of these authors are articulated in the ‘author contributions’ section.
**Competing Interests**<br><br>Use the instructions below to enter a competing interest statement for this submission. On behalf of all authors, disclose any competing interests that could be perceived to bias this	The authors have declared that no competing interests exist.

work—acknowledging all financial support and any other relevant financial or non-financial competing interests.<br><br>This statement is required for submission and **will appear in the published article** if the submission is accepted. Please make sure it is accurate and that any funding sources listed in your Funding Information later in the submission form are also declared in your Financial Disclosure statement.<br><br>View published research articles from *PLOS ONE* for specific examples.<br><br>**NO authors have competing interests**<br><br>Enter: *The authors have declared that no competing interests exist.*<br><br>**Authors with competing interests**<br><br>Enter competing interest details beginning with this statement:<br><br>*I have read the journal's policy and the authors of this manuscript have the following competing interests: [insert competing interests here]*<br><br>* typeset	
**Ethics Statement**<br><br>Enter an ethics statement for this submission. This statement is required if the study involved:<br><br>• Human participants<br>• Human specimens or tissue<br>• Vertebrate animals or cephalopods<br>• Vertebrate embryos or tissues<br>• Field research<br><br>Write "N/A" if the submission does not require an ethics statement.	The study protocol, including methods for invitation, provision of information about the study for volunteers, taking written informed consent and questionnaire regarding current health status and lifestyle, was approved by the Ethic Committee of City Hospital #40, Saint-Petersburg, Russia.

General guidance is provided below. Consult the submission guidelines for detailed instructions. **Make sure that all information entered here is included in the Methods section of the manuscript.**<br><br>**Format for specific study types**<br><br>**Human Subject Research (involving human participants and/or tissue)**<br>• Give the name of the institutional review board or ethics committee that approved the study<br>• Include the approval number and/or a statement indicating approval of this research<br>• Indicate the form of consent obtained (written/oral) or the reason that consent was not obtained (e.g. the data were analyzed anonymously)<br><br>**Animal Research (involving vertebrate animals, embryos or tissues)**<br>• Provide the name of the Institutional Animal Care and Use Committee (IACUC) or other relevant ethics board that reviewed the study protocol, and indicate whether they approved this research or granted a formal waiver of ethical approval<br>• Include an approval number if one was obtained<br>• If the study involved *non-human primates*, add *additional details* about animal welfare and steps taken to ameliorate suffering<br>• If anesthesia, euthanasia, or any kind of animal sacrifice is part of the study, include briefly which substances and/or methods were applied<br><br>**Field Research**<br><br>Include the following details if this study involves the collection of plant, animal, or other materials from a natural setting:<br>• Field permit number<br>• Name of the institution or relevant body that granted permission	
**Data Availability**<br><br>Authors are required to make all data underlying the findings described fully	Yes - all data are fully available without restriction

available, without restriction, and from the time of publication. PLOS allows rare exceptions to address legal and ethical concerns. See the PLOS Data Policy and FAQ for detailed information.<br><br>A Data Availability Statement describing where the data can be found is required at submission. Your answers to this question constitute the Data Availability Statement and **will be published in the article**, if accepted.<br><br>**Important:** Stating 'data available on request from the author' is not sufficient. If your data are only available upon request, select 'No' for the first question and explain your exceptional situation in the text box.<br><br>Do the authors confirm that all data underlying the findings described in their manuscript are fully available without restriction?	
**Describe where the data may be found in full sentences. If you are copying our sample text, replace any instances of XXX with the appropriate details.**<br><br>• If the data are **held or will be held in a public repository**, include URLs, accession numbers or DOIs. If this information will only be available after acceptance, indicate this by ticking the box below. For example: *All XXX files are available from the XXX database (accession number(s) XXX, XXX.*).<br>• If the data are all contained **within the manuscript and/or Supporting Information files**, enter the following: *All relevant data are within the manuscript and its Supporting Information files.*<br>• If neither of these applies but you are able to provide **details of access elsewhere**, with or without limitations, please do so. For example:<br><br>*Data cannot be shared publicly because of [XXX]. Data are available from the XXX Institutional Data Access / Ethics*	Source data of this study are available on request

Committee (contact via XXX) for researchers who meet the criteria for access to confidential data.<br><br>*The data underlying the results presented in the study are available from (include the name of the third party and contact information or URL).*<br>• This text is appropriate if the data are owned by a third party and authors do not have permission to share the data.<br><br>* typeset	
Additional data availability information:	

Powered by Editorial Manager® and ProduXion Manager® from Aries Systems Corporation

Manuscript
Click here to access/download;Manuscript;Hematology RIs for Russian population_Final2.docx

Derivation of Russian-specific reference intervals for complete blood count, iron markers and related vitamins.

Anna Ruzhanskaya[1], Kiyoshi Ichihara[2*], Elena Sukhacheva[3], Irina Skibo[4], Nina Vybornova[4], Dmitry Butlitski[1], Anton Vasiliev[4], Galina Agarkova[1], Ekaterina Vilenskaya[1], Vladimir Emanuel[5], Svetlana Lugovskaya[6]

1. Beckman Coulter Inc., Moscow, Russia;
2. Faculty of Health Sciences, Yamaguchi University Graduate School of Medicine, Ube, Japan;
3. Beckman Coulter Eurocenter, Nyon, Switzerland;
4. Helix laboratory, Saint-Petersburg, Russia;
5. Pavlov First Saint-Petersburg State Medical University, Saint-Petersburg, Russia;
6. Russian Medical Academy of Continuing Professional Education of the Ministry of Health, Moscow, Russia.

Running Title: Hematology reference intervals for Russian population

***Corresponding author:**

*Corresponding author:

Kiyoshi Ichihara, M.D., Ph.D.,

Department of Clinical Laboratory Sciences, Faculty of Health Sciences,

Yamaguchi University Graduate School of Medicine,

Minami-Kogushi 1−1−1, Ube, 755-8505 Japan

Phone: +81-90-7895-0432, Fax: +81-836-35-5213,

E-mail: ichihara@yamaguchi-u.ac.jp

Conflict of interest statement

Anna Ruzhanskaya (AR), Elena Sukhacheva (ES), Dmitry Butlitski (DB), Galina Agarkova (GA) and Ekaterina Vilenskaya (EV) are the employees of Beckman Coulter, Irina Skibo (IS), Nina Vybornova (NV) and Anton Vasiliev (AV) are the employees of Helix Laboratory, but this commercial affiliation does not alter our adherence to PLOS ONE policies on sharing data and research results. In fact, this study was initiated by IFCC, C-RIDL, that promoted harmonized derivation of country-specific RIs around the world. Prof. Kiyoshi Ichihara (KI), the coordinator of the C-RIDL project supported this study all through without any monetary reward whatsoever.

Funding statement

Beckman Coulter, LLC (Moscow, Russia; https://www.beckmancoulter.com/ru) supported the study of the assay reagents and Helix Laboratories Services (Saint-Petersburg, Russia) assisted in the recruitment of volunteers, sample preparations, and provision of sampling equipment. The funder provided support in the form of salaries for authors (AR, ES, DB, GA, EV), but did not take any role in the study design, data collection and analysis, decision to publish, or preparation of the manuscript. The specific roles of these authors are articulated in the ‘author contributions’ section.

Author contributions

KI shared the conceptualization of this study, AR, KI, IS, NV, AV performed the data curation, IS, AR, NV, AV, DB, and EV shared the sample collection, transportation, storage. AR, DB, and GA carried out measurements and controlled over analytical performance and AR took part in data-entry. KI and AR performed the data-analysis. AR and KI wrote the manuscript. All co-authors shared in reviewing the manuscript.

Abstract (300 words)

Objectives

This study was aimed to establish reference intervals (RIs) for Russian adults for hematological parameters including related iron markers and vitamins. Sources of variation of reference values (RVs) and needs for secondary exclusion were explored for proper derivations of RIs.

Methods

Following the harmonized protocol of the IFCC Committee on Reference Intervals and Decision Limits (C-RIDL), 506 healthy Russians (age 18−80; 46% male) were recruited. Complete blood counts (CBC) and leukocyte differentials, iron markers, vitamin B12, and folate were measured by Beckman Coulter's analyzers. Sources of variation were analyzed by multiple regression analysis, and ANOVAs, and the need for partitioning RVs was decided accordingly. Two schemes of excluding latent anemia were compared: (1) latent abnormal values exclusion method (LAVE) by specifying 5−7 reference tests among CBC parameters, or (2) explicit exclusion of individuals with either ferritin or iron below the respective lower limit. RIs were determined by the parametric method using two-parameter Box-Cox power-transformation formula.

Results

Gender-specific RIs were required for most analytes, while age-specific RIs were set only to ferritin in females. BMI-related increase in RVs was observed prominently for reticulocyte parameters, for which we chose to exclude individuals with BMI>28 kg/m^2 when establishing the RIs. The LAVE method was found to be more effective in excluding individuals with latent anemia, than exclusion based on low ferritin and/or iron values. International comparison revealed that Russian RIs featured a lower side shift of platelet counts. As in African countries, Russian RIs for total leukocyte and neutrophil counts were lower compared to most of other countries.

Conclusion

RIs for the Russian population for 34 hematological and related parameters were established using up-to-date methods proposed by C-RIDL. Reducing influences of latent anemia and obesity on RIs was crucial for erythrocyte parameters. Low levels of Russian RIs observed for platelet and neutrophil count need further investigation.

Word count: **300** words

Number of references: 50

4 Figures + 4 Tables

6 Supplemental Tables + 1 Supplemental Figure

Key words: reference value (RV), reference interval (RI), sources of variation; latent abnormal values exclusion method (LAVE); nested ANOVA; standard deviation ratio (SDR); multiple regression analysis (MRA); latent anemia.

Abbreviations:

2N-ANOVA: two-level-nested ANOVA
3N-ANOVA: three-level-nested ANOVA
Mon: monocyte count
Mon%: percentage of monocyte count
Bas: basophil count
Bas%: percentage of basophil count
BC: Beckman Coulter
BMI: body mass index
CBC: complete blood count
CI: confidence interval
CLSI: Clinical and Laboratory Standards Institute
C-RIDL: Committee on Reference Intervals and Decision Limits
CV: coefficient of variation
Eos: eosinophil count
Eos%: percentage of eosinophil count
Fe: iron
Ferr: ferritin
Hb: hemoglobin
Ht: hematocrit
RBC: red blood cell count
RDW: red blood cell distribution width
Ret: reticulocyte count
Ret%: percentage of reticulocyte count
RI: reference interval
r_p: standardized partial regression coefficient
RSF: red blood cell size factor
RUO: research use only
RV: reference value
SD: standard deviation
SDR: standard deviation ratio
Smk: smoking cigarettes
TIBC: total iron binding capacity
TRF: transferrin
TRFSat: transferrin saturation
UIBC: unsaturated iron binding activity
IFU: instruction for use
IRF: immature reticulocyte fraction
LAVE: latent abnormal values exclusion
LHD%: percentage of low hemoglobin density
Lym: lymphocyte count
Lym%: percentage of lymphocyte count
M: male
MAF: microcytic anemia factor
MCV: mean corpuscular volume
MCH: mean corpuscular hemoglobin
MCHC: mean corpuscular hemoglobin concentration
Me: median
MPV: mean platelet count
MRA: multiple regression analysis
MRV: mean reticulocyte volume
Neu: neutrophil count
Neu%: percentage of neutrophil count
NP: non-parametric
P: parametric
PDW: platelet distribution width
PLT: platelet count

VitB12: Vitamin B12
WBC: white blood cell count

Introduction

Interpretation of laboratory test results starts with a comparison of values with reference intervals (RIs) served by laboratory. RIs provided by manufacturers are mostly derived for the American or European population, which may not match local populations. CLSI EP 28 A3 guidelines recommend each laboratory to establish its own reference intervals [1]. With the recent advancement of standardization in laboratory tests, the consensus is to establish reference intervals for common use within a region or country by collaborating with multiple laboratories [2]. Accordingly, the IFCC Committee on Reference Intervals and Decision Limits (C-RIDL) developed an internationally harmonized protocol to facilitate country- or region-specific studies to define RIs [3]. It provides optimal techniques for recruitment, sampling, measurements, secondary exclusion, and statistical analyses to enable reproducible and standardized derivation of RIs. In 2012, C-RIDL launched a global multicenter study on RVs to promote the establishment of country-specific RIs. To make the derived RIs comparable across countries, a value-assigned serum panel was provided to the participating countries [4-13].

We joined the project from Russia by collaborating with laboratories in three major cities to establish Russian population-specific RIs and explore sources of variations of RVs for the major chemistry analytes [12] and for hormones and tumor markers measured by immunoassay [13]. This is our third report targeting RVs of hematological tests, including complete blood counts (CBC), iron markers, and related vitamins.

In the C-RIDL global project targeting biochemistry analytes, the latent abnormal values exclusion (LAVE) method was used to reduce the influence of inappropriate values, attributable to pre-sampling nutritional problems and/or muscular exertion. In the current study we investigated the utility of the LAVE method to reduce the influence of latent iron deficiency anemia in comparison to the conventional approach of explicit exclusion of individuals with low levels of serum ferritin and iron. Additionally, we compared Russian RIs with those reported from other countries.

Materials and Methods

1) Recruitment and samples collection

The study protocol, including methods of invitation, provision of information about the study for volunteers, taking written informed consent and questionnaire regarding current health status and lifestyle, was approved by the Ethics Committee of the City Hospital #40, Saint-Petersburg. The volunteers' recruitment period was started on December 19, 2013, and finished on December 18, 2014. The scheme used for recruitment, sampling, and measurements was described in the first part of our report, which dealt with RIs and sources of variation of chemistry analytes [12].

Briefly, 506 healthy individuals >18 years of age were recruited in Saint-Petersburg (North-West region). The ratio of males to females was 46 to 54%. 99% of the recruited volunteers were Caucasians. They were chosen according to the inclusion and exclusion criteria stipulated in the C-RIDL protocol, and blood samples were drawn at basal conditions [3].

Blood from each volunteer was collected into four 9 mL-vacuum tubes with clot activator and separation gel (Becton Dickinson, USA) and into one 5 mL-vacuum tube with K2-EDTA (Becton Dickinson, USA) for hematology analysis. Serum aliquots prepared from each volunteer were immediately frozen and then stored at −70°C until the time of collective measurements.

2) Target analytes

In total, 34 hematology-related parameters were included in this study, which were grouped as follows:

(2-1) Tests reflecting the metabolism of iron, vitamin B12 and folate: iron (Fe), unsaturated iron-binding capacity (UIBC), total iron-binding capacity (TIBC), transferrin (TRF), transferrin saturation (TRFSat), ferritin (Ferr), vitamin B12 (VitB12), and folate;

(2-2) Tests related to erythrocytes: red blood cell count (RBC), hemoglobin (Hb), hematocrit (Ht), mean corpuscular volume (MCV), mean corpuscular hemoglobin (MCH), mean corpuscular hemoglobin concentration (MCHC), red blood cell distribution width in SD and in CV (RDW-SD, RDW-CV);

(2-3) Tests related to immature erythrocytes: reticulocyte count and percentage (Ret, Ret%), mean reticulocyte volume (MRV), immature reticulocyte fraction (IRF);

(2-4) Tests related to leukocytes: white blood cell count (WBC), neutrophil count and percentage (Neu, Neu%), eosinophil count and percentage (Eos, Eos%), basophil count and percentage (Bas, Bas%), monocytes count and percentage (Mon, Mon%), lymphocytes count and percentage (Lym, Lym%);

(2-5) Tests related to platelet: platelet count (PLT), mean platelet volume (MPV), and platelet distribution width (PDW).

The measurements of Fe, UIBC, TIBC, TRF, and TRFSat were performed on the biochemistry analyzer AU 5800 (Beckman Coulter Inc., USA); ferritin, Vit B12 and folate were measured on the immunochemistry analyzer UniCel DxI 800 (Beckman Coulter Inc., USA) according to the requirements provided in the manufacturer’s instructions for each assay. Hematological tests, or so-called CBC, were performed using the hematology analyzer UniCel DxH 800 (Beckman Coulter Inc., USA) on the day of blood collection. Description of methods and calibrators traceability is provided in **Suppl. Table 1**.

3) Quality Control

All laboratory measurements were done according to the laboratory's Quality Manual and standard operating procedures (SOPs). Instructions described in the manuals of each analyzer and in the reagent/kit

package inserts were adhered to. For clinical chemistry and immunochemistry tests, quality control was performed in two ways. One was through twice-daily measurement of 2 or 3 levels of QC specimens obtained from Beckman Coulter Inc. and Bio-Rad Laboratories, Inc., and the other was through daily measurement of a mini panel, composed of six sera from healthy volunteers (3 women and 3 men), as described in the common protocol and Part 1 and Part 2 of the current study reports [3,12,13]. Additionally, as a part of the study objectives, a worldwide comparison of RVs was performed based on the test results of a serum panel consisting of 40 specimens provided by C-RIDL, measured in four batches over a period of four days. For CBC parameters, special control materials COULTER 6C cell and Retic-X cell provided by the manufacturer were used for monitoring between-day analytical variations.

4) Statistical methods

Data analyses and statistical methods used were those recommended in the C-RIDL protocol [3,14], as described in detail in the first two parts of our report [12, 13]. In brief, the procedures are as follows:

4.1) Analyses of biological sources of variations

The multiple regression analysis (MRA) was used to assess the individual contribution of multiple variation factors on each analyte. It was performed by setting the RVs of each analyte as an objective variable, and the following factors as a fixed set of explanatory variables: sex, age, body mass index (BMI), the levels (see below) of cigarette smoking, alcohol consumption, and regular physical exercise [15]. The smoking, alcohol consumption, and physical exercise levels were classified into 3, 5, and 8 categories, respectively, using the following boundaries: none, $\leq$20, >20 cigarettes/day; none, <12.5, 12.5–25, 25–50, >50 g ethanol/day; none, 1–7 days/week. The contribution of each factor on RVs was expressed using a standardized partial regression coefficient (r_p), which corresponds to the partial correlation coefficient, taking a value between −1.0 and 1.0. In reference to the Cohen's guide [16] on practical significance ("effect size") of correlation, r_p can be interpreted as small $0.1\leq |r_p| <0.3$; medium $0.3\leq |r_p| <0.5$, large $|r_p| \leq 0.5$. Hence, we regarded $|0.3| \leq r_p$ as a significant level of a given factor influencing the RVs.

4.2) Criteria for partitioning RVs.

The standard deviation ratio (SDR) was used as a criterion for assessing the need of partitioning RVs by sex and age. To calculate SDR, pure components of between-sex SD (SD_{sex}) and between-age SD (SD_{age}) were first computed by two-level nested ANOVA and within-group SD (approximately 1/4 the width of RI, or SD_{RI}). The SDRs for sex or age (SDR_{sex}, SDR_{age}) were calculated by taking respective ratios of SD_{sex} and SD_{age} to SD_{RI}. Since SDR_{age} often differs between sexes, it was also calculated by one-way ANOVA after partitioning RVs by male and female, as SDR_{ageM}, SDR_{ageF}. The need for partition of RVs was considered by setting $SDR\geq0.4$ as a primary guide [3].

However, SDR can be too sensitive, when the width of RI constituting the denominator of SDR is narrow. Conversely, SDR may be insensitive, when between-subgroup differences occur only at the periphery of distribution (LL or UL) — SDR represents between-subgroup differences at the center of the distributions. Therefore, we additionally considered actual difference (bias) at LL or UL as "bias ratio" (BR) using the following formula, illustrating a case of gender difference:

$$\mathrm{BR_{LL}} = \frac{|LL_M - LL_F|}{(UL_{MF} - LL_{MF})/3.92}, \quad \mathrm{BR_{UL}} = \frac{|UL_M - UL_F|}{(UL_{MF} - LL_{MF})/3.92}$$

where subscript M, F, and MF represent male, female, and male+female, respectively. The denominator of each formula represents the standard deviation (SD_{RI}) comprising the RI, the width of which corresponds to 3.92 times SD_{RI}. To make the threshold of BR aligned to SDR with the common denominator (SD_{RI}), we multiplied the threshold of SDR=0.4 by $\sqrt{2}$ to obtain BR's threshold of 0.57 (i.e., absolute difference (bias) of two values (x_1 and x_2), or $|x_1 - x_2|$, is $\sqrt{2}$ times larger than SD of the two values).

Whereas BR was used for judging the effectiveness of the LAVE method, its threshold was set according to the conventional specification of allowable analytical bias (=difference) of a minimum level: $0.375 \times \sqrt{SD_G^2 + SD_I^2}$ (= SD_{RI}) [15], and thus 0.375 was set as the threshold for BR rather than 0.57 [17].

4.3) Derivation of reference intervals.

RIs were derived by both parametric and nonparametric methods for comparative purposes. The former was performed after the Gaussian transformation of RVs using the two-parameter Box-Cox power transformation formula [16]. The 90% confidence interval (CI) of the lower limit (LL) and upper limit (UL) of RI was calculated by the bootstrap method through random resampling of the same dataset 50 times. Accordingly, the final LL and UL of RI, derived both by parametric and nonparametric methods, were chosen as the average of iteratively derived LLs and ULs. The RIs derived by the nonparametric method were adopted only when the parametric method failed in Gaussian transformation.

To reduce the influence of latent iron deficiency, the following two methods of secondary exclusion were implemented and compared:

1) Latent abnormal values exclusion (LAVE) method with an iterative exclusion of individuals with abnormal results among reference tests associated with erythrocyte parameters. Two sets of reference tests for the LAVE method were chosen based on Spearman's correlation matrix shown in **Suppl Table 2** [see Discussion] for comparative purposes:

#1: Seven reference tests with iron markers: Hb, MCH, MCHC, RDW-CV, Ret, Fe, and ferritin.

#2: Five reference tests without iron markers: Hb, MCH, MCHC, RDW-CV, and Ret

In both schemes, no abnormal results among the reference tests were allowed, but the RIs of respective tests were empirically extended to 3% of the width of the RI on both limits (i.e., LL'= LL –0.03×

(UL−LL)/3.92; UL’= UL +0.03× (UL−LL)/3.92) so as not to delete too many records. While the number of iterative derivations was set to 6.

2)) Explicit exclusion of individuals with IDA as defined, with either ferritin or Fe being below respective decision limit, provided in the Access Ferritin reagent instructions for use (IFU), and for the AU Fe reagent (Beckman Coulter Inc.) (i.e., for ferritin <23.9 µg/L (male) and <11 µg/L (female); for Fe<12.5 µmol/L (male) and <10.7 µmol/L (female)) [18,19].

For the leukocyte and platelet-related parameters, we performed a preliminary analysis on the effectiveness of the LAVE method in reducing the influence of latent infectious conditions on their test results in reference to the results of inflammatory markers: TF, and ferritin. However, the LAVE method did not cause much change in reference limits. Spearman’s correlation coefficient matrix built with leukocyte and platelet parameters did not show many cross-associations among them. These findings led us to interpret, that subclinical infectious conditions among healthy subjects were not as frequent as to affect the derivation of RIs for those analytes. Hence, we did not apply the LAVE method to the analytes related to leukocyte and platelet (**Suppl Table 2**).

Results

1. Sources of variation of reference values

Sources of variations of each analyte were evaluated by MRA and ANOVA as described in part 1 of our report [12].

1.1 Multiple regression analysis (MRA) to explore sources of variation of RVs

The r_{ps} computed by MRA analyte by analyte for 34 hematology-related parameters are presented in **Table 1**. By setting $|r_p| \geq 0.3$ as practical importance, positive association of RVs with age was observed for 2 analytes in males, including MCV and RDW-SD (r_p = 0.306 and 0.376, respectively) and for 4 analytes in females, with age-related decrease of RVs for TIBC, TF, and Mon (−0.339, −0.330, and −0.320, respectively) and age-related increase of RVs for ferritin (0.391).

A significant BMI-related increase in RVs was observed for Ret, Ret% and RBC in both sexes (r_p = 0.467, 0.431, and 0.302 for males; 0.591, 0.543, 0.308 for females). In males, BMI-related increase was also observed for TIBC and TF (0.331 and 0.313), in females for IRF (0.429). In contrast, no apparent influence of alcohol consumption, smoking and physical activities was found based on the MRA.

Table 1. Results of multiple regression analysis for sources of variation of RVs.

	Male							Female						
Analyte	n	R	Age	BMI	DrkLvl	SmkLvl	ExerLvl	n	R	Age	BMI	DrkLvl	SmkLvl	ExerLvl
Fe	224	0.138	0.110	0.001	-0.097	0.038	-0.026	262	0.211	0.038	-0.031	0.192	0.057	-0.029
UIBC	193	0.245	-0.066	0.247	0.030	-0.012	-0.042	225	0.279	-0.241	0.128	-0.169	0.026	0.093
TIBC	191	0.334	-0.087	**0.331**	-0.069	0.009	-0.005	225	0.327	**-0.339**	0.189	-0.098	0.059	0.093
TF	221	0.308	-0.110	**0.313**	-0.036	0.020	-0.024	261	0.331	**-0.330**	0.199	-0.115	0.098	0.053
TRFSat	192	0.123	0.089	-0.077	-0.056	0.046	0.022	223	0.228	0.133	-0.035	0.188	0.018	-0.060
Ferr	221	0.239	-0.086	0.173	0.129	-0.079	-0.088	262	0.580	**0.391**	**0.252**	0.176	0.066	-0.068
VitB12	221	0.253	0.031	0.123	-0.061	-0.013	0.194	257	0.215	0.098	0.092	0.010	-0.005	0.140
Folate	223	0.313	0.198	0.104	-0.001	-0.117	0.117	261	0.129	0.006	0.073	0.011	-0.010	0.100
RBC	223	0.421	-0.155	**0.302**	-0.219	-0.084	-0.201	262	0.298	-0.040	**0.308**	-0.045	-0.016	0.016
Hb	224	0.339	-0.010	**0.265**	-0.182	0.077	-0.144	261	0.341	0.166	0.225	0.057	0.056	-0.005
Ht	224	0.331	0.022	**0.260**	-0.158	0.097	-0.138	259	0.336	0.116	**0.263**	0.039	0.054	0.008
MCV	223	0.416	**0.306**	-0.181	0.064	0.235	0.117	251	0.302	0.204	0.017	0.124	0.172	0.077
MCH	220	0.345	**0.254**	-0.169	0.016	0.183	0.137	243	0.266	**0.256**	-0.034	0.086	0.097	0.045
MCHC	219	0.223	-0.137	-0.003	-0.082	-0.133	0.009	257	0.217	0.233	-0.055	0.038	0.006	-0.035
RDW-SD	222	0.484	**0.376**	-0.085	0.114	**0.261**	0.065	260	0.138	0.098	0.018	-0.009	0.097	-0.013
RDW-CV	224	0.308	0.218	0.089	0.035	0.158	-0.010	246	0.146	-0.079	-0.029	-0.057	-0.074	-0.064
Ret	162	0.464	-0.089	**0.467**	-0.036	-0.036	-0.115	233	0.585	-0.017	**0.591**	0.014	0.075	0.026
Ret%	161	0.424	-0.057	**0.431**	0.012	-0.025	-0.083	232	0.542	-0.008	**0.543**	0.033	0.073	0.031
MRV	160	0.302	**0.255**	-0.178	0.043	0.120	0.065	233	0.230	0.161	-0.171	0.084	0.107	0.094
IRF	163	0.320	0.090	0.220	0.008	0.151	0.123	233	0.432	-0.003	**0.429**	0.054	0.078	-0.021
WBC	199	0.384	-0.011	0.231	-0.167	0.217	-0.166	259	0.278	-0.233	**0.275**	-0.007	0.051	-0.099
Neu	220	0.323	0.060	0.197	-0.091	0.203	-0.113	259	0.259	-0.168	**0.269**	0.018	0.081	-0.075
Eos	201	0.225	-0.063	0.026	-0.057	0.173	-0.081	260	0.155	-0.060	0.121	-0.007	-0.058	-0.103
Bas	201	0.167	0.098	0.023	-0.122	0.089	-0.019	262	0.178	-0.192	0.060	-0.023	-0.058	-0.033
Mon	199	0.274	-0.015	0.135	-0.161	0.193	-0.028	262	0.342	**-0.320**	**0.287**	-0.002	0.006	-0.163
Lym	199	0.325	-0.155	0.202	-0.131	0.069	-0.178	261	0.279	**-0.279**	0.203	-0.069	0.041	-0.096
Neu%	223	0.224	0.170	0.045	-0.007	0.129	0.025	261	0.184	-0.013	0.150	0.046	0.105	0.015
Eos%	224	0.140	-0.056	-0.068	0.028	0.080	-0.039	260	0.123	0.049	-0.010	0.005	-0.100	-0.049
Bas%	221	0.128	0.065	-0.025	-0.052	-0.101	0.014	259	0.181	-0.044	-0.129	-0.028	-0.096	0.023
Mon%	221	0.146	0.028	-0.025	-0.051	0.007	0.134	261	0.131	-0.095	0.020	0.011	-0.093	-0.064
Lym%	224	0.268	-0.200	-0.001	0.033	-0.182	-0.058	261	0.178	-0.039	-0.125	-0.080	-0.063	0.015
PLT	224	0.135	-0.036	-0.005	-0.003	-0.014	-0.130	262	0.225	-0.208	0.188	0.042	-0.014	-0.110
MPV	223	0.289	**0.258**	-0.060	-0.071	0.078	-0.110	262	0.133	-0.093	0.100	0.006	0.049	0.068
PDW	222	0.251	-0.181	0.187	0.011	0.010	0.101	262	0.064	0.038	-0.021	-0.022	0.004	-0.046

The listed values are standardized partial correlation coefficients (r_p)
R: multiple regression coefficient; BMI: body-mass index; DrkLvl, SmkLvl, ExerLvl: grades of drinking, smoking, and regular exercise

1.2 The magnitude of sex, age, BMI-related changes in RVs computed by ANOVA

SDR_{sex}, a magnitude of between-sex differences in RVs, was computed by 2-level nested ANOVA in two ways, either with age or BMI as a covariate. The between-age and between-BMI differences in RVs were computed as SDR_{age} and SDR_{BMI} by one-way ANOVA, separately for each sex (**Table 2**), where age and BMI were subgrouped respectively at the boundary of 30, 40, and 50 years of age, and 20, 25, and 30 kg/m^2.

By setting SDR $\geq$ 0.4 as a threshold, the sex-related differences were significant in eight analytes: UIBC, TF, TFRSat, ferritin, RBC, Hb, Ht, and MCHC (SDR_{sex} = 0.478, 0.426, 0.429, 1.252, 1.106, 1.381, 1.369, and 0.460 respectively).

Table 2. List of SDRs representing between-subgroup variations by sex, age, and BMI:

2.1 Sex and age-related changes

	2N-ANOVA		1way-ANOVA	
**Analyte**	**SDRsex**	**SDRage**	**SDRageM**	**SDRageF**
**Fe***	**0.356**	0.000	0.000	0.000
**UIBC***	**0.478**	0.045	0.000	0.124
**TIBC***	**0.338**	0.206	0.000	0.265
**TF***	**0.426**	0.205	0.000	0.272
**TRFSat***	**0.429**	0.000	0.000	0.000
**Ferr***	**1.252**	**0.491**	0.000	**0.656**
**VitB12***	0.175	0.172	0.186	0.161
**Folate***	0.276	0.182	0.213	0.152
**RBC**	**1.106**	0.162	0.177	0.148
**Hb***	**1.381**	0.237	0.000	**0.334**
**Ht**	**1.369**	0.223	0.071	0.280
**MCV**	0.061	0.256	**0.308**	0.216
**MCH**	0.212	0.235	0.224	0.243
**MCHC**	**0.460**	0.249	0.264	0.241
**RDW-SD***	0.000	**0.337**	**0.470**	0.145
**RDW-CV***	0.256	0.143	0.292	0.000
**Ret***	**0.339**	0.226	0.000	**0.305**
**Ret%***	0.000	0.223	0.030	0.284
**MRV**	0.000	0.161	0.234	0.100
**IRF**	0.000	0.270	0.255	0.279

	2N-ANOVA		1way-ANOVA	
**Analyte**	**SDRsex**	**SDRage**	**SDRageM**	**SDRageF**
WBC*	0.167	0.000	0.000	0.042
Neu*	0.000	0.001	0.000	0.037
Eos*	0.219	0.118	0.043	0.158
Bas*	0.000	0.149	0.023	0.203
Mon*	**0.414**	0.116	0.000	0.201
Lym*	0.262	0.171	0.112	0.205
Neu%	0.250	0.169	0.193	0.149
Eos%*	0.172	0.102	0.068	0.130
Bas%	0.134	0.031	0.000	0.109
Mon%	**0.367**	0.000	0.000	0.000
Lym%	0.099	0.205	0.228	0.185
PLT	**0.317**	0.046	0.060	0.037
MPV*	0.129	0.237	**0.327**	0.063
PDW	0.171	0.158	0.147	0.164

2.2 Sex and BMI-related changes

	2N-ANOVA		1way-ANOVA	
	**SDRsex**	**SDR_{BMI}**	**SDR_{BMI}M**	**SDR_{BMI}F**
RBC	**1.134**	**0.375**	**0.340**	**0.402**
Hb*	**1.398**	**0.331**	0.249	**0.376**
Ret*	0.168	**0.691**	**0.602**	**0.743**
Ret%*	0.000	**0.625**	**0.568**	**0.657**
IRF	0.000	**0.451**	**0.310**	**0.523**

* Gaussian transformation done before the analysis

Between-gender differences are clearly observed for 20 analytes as shown in **Fig. 1.** For age-related changes, significant differences were observed for the following analytes: ferritin in females (SDR_{age} =0.656), and RDW-SD in males (SDR_{age} = 0.47), as illustrated in **Fig. 2.** BMI-related differences in RVs were significant for Ret, Ret% and IRF: i.e., SDR_{BMI} =0.691, 0.625, and 0.451 respectively, calculated for both genders with 2-level nested ANOVA. While SDR_{BMI} calculated for each gender by one-way ANOVA for Ret and Ret% were 0.602 and 0.568, respectively, in males, and 0.743 and 0.657, respectively, in females; for IRF and RBC SDR_{BMI} = 0.523 and 0.402, respectively, in females. BMI-related changes in RVs that were significant according to MRA ($r_p \geq 0.3$) are illustrated for RBC, Ret, Ret%, and IRF in **Fig. 2**, in which BMI values were stratified at < 20, 20–25, 25–30, ≥30 kg/m^2.

Fig. 1. Between-gender differences for major parameters

Gender-specific distributions of RVs for major parameters are drawn by scattergrams. The background shades represent RIs determined for male (blue), female (pink), and for both (purple). The central 50% range and median of RVs are shown by the horizontal box and the vertical line.

Fig 2. Hematology RVs influenced by BMI and age

RVs of RBC, Ret, Ret%, and IFR were partitioned by gender and BMI into five groups, while RVs were partitioned by age at 45 years. The central 50% range and median of RVs are shown by the horizontal box and the vertical line. No secondary exclusion was done while drawing the scattergrams.

1.3. Derivation of reference intervals

To find an optimal method of secondary exclusion of latent anemia, the following four methods were compared for derivation of RIs of erythrocyte parameters:

1) No exclusion was performed;

2) LAVE method using 7 reference tests（Fe, Ferr, Hb, MCH, MCHC, RDW-CV, Ret);

3) LAVE method using 5 reference tests (Hb, MCH, MCHC, RDW-CV, Ret);

4) Explicit exclusion of patients who belonged to IDA group by the criteria described in the Methods.

The distribution of RV datasets after selection by 1) to 4) are shown in **Fig. 3**. for six representative analytes as a scattergram located in the 1st, 2nd, 4th, and 6th row, respectively.

As a reference, the following three datasets were prepared and plotted in the 3rd, 5th, and 7th rows: i.e., 3rd for datasets of patients with abnormal results among 7 reference tests, 5th for patients with abnormal results among 5 reference tests, and 7th for patients belonging to the IDA group.

Fig.3 The effect of LAVE on RIs for hematology tests in comparison to IDA criteria

Female RIs of 6 representative hematology tests were determined parametrically using the following 4 sets of RV datasets.
1: All: RVs without any prior exclusion
2: LAVE(-) 7RT: RVs with no abnormal value among 7 tests (Hb, MCH, MCHC, RDW-CV, Ret, Fe, Ferr) other than itself
4: LAVE(-) 5RT: RVs with no abnormal value among 5 ref tests (Hb, MCH, MCHC, RDW-CV, Ret) other than itself
6: IDA(-): RVs with no abnormal results for Fe and Ferr from respective lower limit of the manufacturer's RI.
Each bar shown over the scattergram represents the span of RI with its lower and upper limits indicated by numerical labels. The three scattergrams of RVs shown in red dots in the 3rd, 5th, and 7th rows represent values that were excluded by the criteria applied to obtain RVs shown above in the 2nd, 4th, and 6th rows in green. Thus, their group labels were designated as follows:
As a reference, the following three datasets were prepared and plotted in the 3rd, 5th , and 7th row.
3: LAVE(+) 7RT: RVs with one or more abnormal values among 7 ref tests (Hb, MCH, MCHC, RDW-CV, Ret, Fe, Ferr)
5: LAVE(+) 5RT: RVs with one or more abnormal values among 5 ref tests (Hb, MCH, MCHC, RDW-CV, Ret)
7: IDA(+): RVs had abnormal results either for Fe or Ferr.

As a result, the fifth set of reference tests (Hb, MCH, MCHC, RDW-CV, and Ret) was found most efficient in terms of adjusting the effect of RI limits for erythrocyte parameters, iron markers and related vitamins, and the data size that remained after applying the LAVE method (i.e., the use of seven reference tests caused more reduction in sample size for calculating RIs, despite minor differences in RI limits), while the exclusion based on IDA criteria was regarded less efficient in adjusting RI limits.

The effect of partitioning RVs by sex and age on RIs is summarized in **Suppl Table 3** for erythrocyte parameters, iron markers and related vitamins, and in **Suppl Table 4** for leukocytes and platelets. The choice of partitioning by sex and age was primary decided based on SDR>0.4, r_p>0.3, and the effect of partition was also assessed from BR_{LL} or BR_{UL}, by setting its threshold of >0.57. Based on this information, partitioning RVs by sex was performed for 20 analytes, including RBC, Hb, Ht, MCV, RDW-CV, Fe, UIBC, TIBC, TF, TFRSat, ferritin, VitB12, Folate, Eos, Eos%, Mon, Mon%, Lym, and PLT. RIs were partitioned by age for ferritin in females (**Suppl. Table 5**).

Among analytes with BMI-related changes (Hb, Ht in females, and RBC, Ret, Ret% in both sexes), exclusion of individuals with BMI>28 was found effective in lowering UL judged from BR_{UL} (**Suppl. Table 6**).

Regarding the effect of the LAVE method on RI limits (by setting five reference tests: Hb, MCH, MCHC, RDW-SD, and Ret), which is shown in **Suppl. Table 4**, we regarded the following tests as "LAVE-sensitive": RBC, Hb, Ht, MCV, RDW-CV, Fe, UIBC, TF (<45 years) in females, ferritin in males, and MCH, MCHC, RDW-SD, TIBC, TRFSat in both sexes.

After applying all these schemes of partitioning RVs by sex or age, and of secondary exclusion of RVs by LAVE and/or BMI restriction, the final RIs for all the 34 parameters were summarized in **Table 3.**

Table 3 The list of RIs adopted for hematological parameters by sex and age

Item	Unit	LAVE	BMI	Sex	Age	N	LL_L	LL_H	LL	Me	UL	UL_L	UL_H
RBC	10^6 /µL	LAVE(+)	<28	M	All	124	4.41	4.53	4.46	4.92	5.46	5.39	5.51
		LAVE(+)	<28	F	All	132	3.84	3.94	3.88	4.34	4.94	4.83	5.05
Hb	g/L	LAVE(-)		M	All	223	132	137	134	151	166	165	168
		LAVE(+)	<28	F	All	139	112	116	114	130	148	145	151
Ht	%	LAVE(+)		M	All	179	39.7	40.5	40.1	44.0	48.3	47.8	48.8
		LAVE(+)	<28	F	All	132	33.6	34.7	34.1	38.4	42.5	42.0	42.9
MCV	fL	LAVE(-)		M	All	224	81.4	82.8	82.3	88.6	96.5	95.6	98.4
		LAVE(+)		F	All	179	81.9	83.2	82.5	88.5	94.2	93.7	94.8
MCH	pg	LAVE(+)		MF	All	364	27.8	28.2	28.2	30.3	32.7	32.5	32.9
MCHC	g/L	LAVE(+)		MF	All	370	327	331	330	341	353	352	355
RDW-SD	fL	LAVE(+)		MF	All	351	37.5	37.9	37.7	40.9	45.5	45.1	46.0
RDW-CV	%	LAVE(-)		M	All	222	12.0	12.2	12.2	13.1	14.2	14.2	14.5
		LAVE(+)		F	All	189	12.2	12.4	12.3	13.2	15.0	14.9	15.5
Ret	10^9/L	LAVE(-)	<28	MF	All	270	21.3	24.8	23.0	45.6	84.1	79.8	87.1
Ret%	%	LAVE(-)	<28	MF	All	269	0.52	0.57	0.54	1.01	1.77	1.70	1.85
MRV	fL	LAVE(-)		MF	All	392	96.9	98.2	97.6	106.8	117.8	117.1	119.3
IRF	%	LAVE(-)		MF	All	396	23.4	25.5	24.5	36.8	49.8	48.7	50.9
Fe	µmol/L	LAVE(-)		M	All	225	8.5	10.6	9.8	18.8	35.1	32.5	37.3
		LAVE(+)		F	All	177	6.0	7.8	7.2	16.4	30.5	29.0	33.7
UIBC	µmol/L	LAVE(-)		M	All	195	17.4	21.6	18.7	37.8	54.1	52.7	57.5
		LAVE(+)		F	All	155	22.8	27.9	26.2	42.9	63.5	60.2	65.8
TIBC	µmol/L	LAVE(+)		M	All	157	44.2	46.8	46.0	57.3	71.4	69.2	73.6
		LAVE(+)		F	All	155	44.9	47.8	47.0	59.6	78.4	75.5	81.0
TF	g/L	LAVE(-)		M	All	225	1.80	1.92	1.88	2.42	3.16	3.07	3.22
		LAVE(+)		F	All	178	1.85	1.98	1.94	2.59	3.57	3.43	3.70
TRFSat	%	LAVE(+)		M	All	157	12.9	18.1	16.6	33.1	64.3	58.7	67.6
		LAVE(+)		F	All	155	10.2	13.3	11.5	27.5	51.3	48.4	56.7
Ferr	µg/L	LAVE(+)		M	All	173	20.9	32.6	29.8	97	271	251	381
		LAVE(-)		F	~45	150	4.7	5.8	5.5	15	67	56	94
		LAVE(-)		F	45~	111	4.7	5.9	5.3	37	223	174	261
VitB12	pmol/L	LAVE(-)		M	All	223	99	127	113	263	549	519	653
		LAVE(-)		F	All	259	110	132	125	293	710	647	761
Folate	ng/mL	LAVE(-)		M	All	225	2.78	3.31	3.15	6.22	16.5	14.1	18.0
		LAVE(-)		F	All	261	3.29	3.69	3.58	7.51	21.1	18.3	22.7
WBC	10^3 /µL	LAVE(-)		MF	All	457	3.37	3.63	3.54	5.71	9.21	8.86	9.72
Neu	10^3 /µL	LAVE(-)		MF	All	480	1.53	1.70	1.67	3.06	5.85	5.73	6.42
Eos	10^3 /µL	LAVE(-)		M	All	200	0.042	0.054	0.050	0.15	0.59	0.524	0.689
		LAVE(-)		F	All	257	0.033	0.043	0.041	0.13	0.38	0.342	0.430
Bas	10^3 /µL	LAVE(-)		MF	All	460	0.010	0.012	0.011	0.039	0.092	0.090	0.100
Mon	10^3 /µL	LAVE(-)		M	All	200	0.30	0.32	0.31	0.51	0.88	0.84	0.95
		LAVE(-)		F	All	260	0.25	0.27	0.27	0.43	0.73	0.70	0.79
Lym	10^3 /µL	LAVE(-)		M	All	200	1.15	1.28	1.23	2.01	3.55	3.26	3.79
		LAVE(-)		F	All	260	1.04	1.16	1.13	1.78	3.17	2.91	3.33
Neu%	%	LAVE(-)		MF	All	485	36.5	39.9	38.8	54.1	70.1	68.9	71.8
Eos%	%	LAVE(-)		M	All	226	0.70	0.91	0.80	2.59	8.66	7.84	9.39
		LAVE(-)		F	All	259	0.63	0.82	0.75	2.24	6.56	5.80	7.37
Bas%	%	LAVE(-)		MF	All	483	0.19	0.22	0.21	0.67	1.51	1.44	1.65
Mon%	%	LAVE(-)		M	All	222	5.61	6.16	5.93	8.64	12.42	12.07	12.89
		LAVE(-)		F	All	260	4.64	5.11	4.91	7.82	11.29	10.90	11.92
Lym%	%	LAVE(-)		MF	All	484	19.2	20.8	20.6	33.8	47.9	47.2	50.3
PLT	10^3 /µL	LAVE(-)		M	All	222	108	135	132	205	288	279	302
		LAVE(-)		F	All	261	120	142	135	227	330	318	339
MPV	fL	LAVE(-)		MF	All	487	7.35	7.50	7.41	9.14	11.8	11.64	12.03
PDW	%	LAVE(-)		MF	All	406	15.8	16.0	16.0	16.8	17.9	18.0	18.5

Discussion

The main goals of the current study were the following: 1) to explore sources of variation of RVs (sex, age, and BMI) for better interpretation of hematology test results, 2) to derive RIs for a heterogeneous group of tests in the most rational way by applying an analyte-specific scheme of partitioning, and the secondary exclusion of latent diseases, and 3) to grasp the international perspective of Russian RIs by comparing to those reported in other studies or provided in clinical guidelines.

1. BMI-related changes in RVs

RVs of ferritin were strongly associated with age in females (SDRage = 0.656). This age-related increase in ferritin is well-known and can be explained by the cessation of menstrual losses. Ferritin is an inflammatory marker, and its level increases in elderly females [20].

A significant BMI-related increase in RVs of Ret and Ret% was conspicuously observed in both sexes (SDR_{BMI} = 0.60 and 0.57 in males; 0.74 and 0.66 in females, respectively). This finding wasn't regarded as confounded by the age-related increase in BMI, because MRA also showed an independent association of BMI with Ret and Ret% (r_p =0.47 and 0.43 in males; 0.59 and 0.54 in females, respectively) after adjusting for age. The effect of BMI was also detected in a lesser degree for RBC in both sexes, and for Hb and Ht in females. It could be explained by recent findings dedicated to the role of hepcidin, a hormonal peptide secreted by the liver and the adipocytes, in regulating iron homeostasis and erythrocyte production. High hepcidin levels block intestinal iron absorption and macrophage iron recycling, causing iron-restricted erythropoiesis and anemia [21]. This is essential for patients with increased BMI and a large volume of adipose tissue. Perez et al., showed that the hemoglobin and erythrocyte indices did not vary with body weight, and suggested that obesity and the resultant inflammatory state lead to iron deficiency, which, in turn, provoked reticulocytosis [22]. Meanwhile, Jeong et al., demonstrated a positive association of RBC count, Hb, and Ht levels with BMI [23]. This observation is matched with a report by He et al., that Hb could play a role in predicting new-onset metabolic syndrome [24].

Several studies reported that Hb levels were significantly associated with high blood pressure. Thus, Shimizu et al., reported that Ret levels in elderly Japanese individuals were positively associated with hypertension, which was associated with BMI. They postulated that the increased BMI causes oxidative stress in arterial walls, and may induce hematopoiesis and reticulocytosis, which was regarded as having an antioxidant effect [25]. The mechanism underlying the increased hematology parameters, including reticulocytes, in obesity is not fully known, and further investigations are necessary.

2. Comparison of Russian RIs with those reported by other countries

In **Table 4**, we summarized (some) RIs reported for hematology parameters and anemia-associated markers, as well as those provided by the manufacturer in the IFU. In the Turkish, Kenyan, Ghanian, Japanese, Saudi, Chinese and Indian studies [4-11], RIs were derived by the parametric method with the application of LAVE procedure according to the harmonized protocol provided by the IFCC C-RIDL. While in Canadian, Malaysian, African (Asmara), Moroccan, Omani, and Ethiopian studies, RIs were determined nonparametrically according to the CLSI guideline [26-32]. Additionally, we included hematology RIs commonly used across the Russian laboratories that are published in the Russian Methodological guidelines [33,34], as well as the cutoff values that are provided in anemia-related guidelines [35-40].

For ease of comparison, the differences in the spans of RIs are shown by bar-charts in **Fig 4 A** and **B** for representative parameters.

2.1 Erythrocyte and iron markers

The World Health Organization (WHO), the American Gastroenterological Association (AGA) and the Russian National Hematology Society (RNHS) define iron deficiency anemia as blood Hb levels below 130 g/L in men and below 120 g/L in nonpregnant women, and ferritin levels < 15 µg/L (in WHO and AGA guidelines) and < 11.0 µg/L in RNHS, in addition to the related clinical symptoms [35-37].

Based on the results of the current study, LL for Hb in males was slightly higher compared to the international guidelines listed above, and to the IFU (134 vs. 130 and 125 g/L, respectively), but comparable to other studies [4-9, 26-32]. The highest LLs and ULs for Hb and RBC were reported from Kenya and Ethiopia [7,32]. This corresponds to Muckenthaler et al., who demonstrated that Hb, RBC and ferritin levels significantly rose with every 300-m increase in residential altitude, starting from as low as 300 m above the sea level [41] (Saint-Petersburg is located at the altitude of 3 m, vs 1795 m and 2355 m of Nairobi and Addis Ababa, respectively). Similar effects of high altitude were also reported in the Turkish multicenter study [6]. Omuse et al., also mentioned that the Kenyan study included urban population only with sufficient nutrition and easy access to medical treatment. Therefore, their RIs limits for Hb and RBC in both sexes are raised in comparison to other African studies (**Table 4, Fig 4 A, B**) [7]. Their interpretation of raised levels of erythrocyte parameters by nutrition was supported by our findings of BMI-related raise of those RIs.

Ferritin LL in males (29.8 µg/L) in this study was much higher compared to the cutoffs provided by WHO, AGA and RNHS guidelines (15, 15 and 11 µg/L, respectively) [35-37], to the Turkish study (13 µg/L) [6] and comparable to the LL in the IFU (23.9 µg/L) [18].

In Russian females we observed a slightly reduced LL of Hb in comparison to the AGA and RNHS guidelines (114 vs. 120 g/L) [36,37], meanwhile significantly lower ferritin LL was observed in both age groups <45 and ≥45 years (5.5 and 5.3 vs. 15 µg/L, respectively) [35-37]. Even a lower level of ferritin

was reported in the Turkish and Japanese studies (4.3 and 3 µg/L, respectively) [5,6]. Low levels of ferritin in age group <45 years are associated with reduced iron storage due to menstrual bleeding, pregnancy or latent anemia [8]. Bawua et. al. showed age-related increase in ferritin and decrease in TFR, which was confirmed in this study. However, LL for ferritin in Russian females for the age group >45 is still very low (5.3 µg/L). LL for Fe in Russian females (7.2 µmol/L) was also low compared to the IFU (10.7 µmol/L), but twice higher than those reported from Japan, Turkey, China, and India (4.0, 3.5, 5.0 and 4.0 µmol/L, respectively) (**Table 4**, **Fig 4B**). The discrepancy is attributable to the fact, that those studies did not refer to ferritin and CBC results while establishing RIs for Fe. In any case, these findings generally point to a high prevalence of latent IDA among Russian population, as was shown in the WHO report (21.1% prevalence of IDA among non-pregnant Russian women of reproductive age) [42].

Table 4 Comparison of standardized RIs from this study with those from other studies

Analyte	Unit	Sex	Russian study[a]	Beckman IFU[a]	Russian Methodological guidelines[d,e] (33,34)	Kenyan study[f] (7)	Turkish study[g,h,i] (6)	Ghanian study[j] (8,31)	Japanese study[a,o] (5)	Canadian study CALIPER[m] (26)	Malaysian study[a,o] (27)	Erithea (Asmara), Africa[g] (28)	Saudi Arabian study[i] (9)	Moroccan study[r] (29)	Ethiopian study[s] (32)	Omani study[i,t] (30)
RBC	x10^6/µL	M	**4.46-5.46**	4.06 - 5.63	4.0 - 5.0	4.94 - 6.52	4.43 - 6.07	4.57-6.5	4.35-5.55	Age15-49: 4.3-5.7<br>Age50-79: 4.2-5.5	<60yo: 4.53-5.95<br>>60yo: 3.86-5.62	4.2 - 6.07	4.56-6.22	4.37-5.96	4.26-6.68	4.45-6.75
		F	**3.88-4.94**	3.63 - 4.92	3.9 - 4.7	4.31 - 5.76	3.96 - 5.31	4.0-5.46	3.86-4.92	Age15-49: 3.8-5.0	3.87 - 5.21	4.0 - 5.7	3.94-5.25	3.86-5.2	4.02-6.15	4.07-6.17
		MF		3.73 - 5.5		4.41 - 6.48						4.07 - 6.02				
Hb	g/L	M	**134-166**	125 - 163	130 - 160	145 - 187	131 - 175	128-172	137-168	136-169	<60yo: 135-174<br>>60yo: 118-169	126 - 178	136-180	130-171	120.6-187.6	124-164
		F	**114-148**	109 - 143	120 - 140	120 - 165	110 - 152	107-143	116-148	N/A	116 - 151	125 - 176	110-152	111-148	123-178.6	111-151
		MF		114 - 159		128 - 190						126 - 177				
Ht	%	M	**40.1-48.3**	36.7 - 47.1	40 - 48	43 - 55	39.2 - 52.2	39.4-52.1	41-50	40-50	<60yo: 40.1-50.6<br>>60yo: 35.7-48.9	40.5 - 55.0<br>37.9 - 52	40.2-52.0	38.3-50	36.72-54.48	36-47
		F	**34.1-42.5**	31.2 - 41.9	36 - 42	36 - 49	33.7 - 46.1	34.0-44.2	35-44	N/A	35.1 - 44.9	38.3 - 54.4	33.6-44.5	33.5-43.9	36.86-51.59	33-43
		MF		33.3 - 45.7		38 - 55										
MCV	fL	M	**82.3-96.5**	73.0 - 96.2		76.5 - 95.5				82.5-98.0		85.7 - 100		77.4-94.2	74.8-93.94	
		F	**82.5-94.2**	75.5 - 95.3		73.4 - 95.8				82.5-98.0		85.5 - 100		75.1-94.7	77.3-98.82	
		MF		73.7 - 95.5	80.0 - 100.0	75.7 - 95.6	77.2 - 95.7	71.9-97.4	83.6-98.2		80.6 - 95.5	85.8 - 100	75-94.4			62.5-88.5
MCH	pg	M		23.8 - 33.4		25.1 - 32.8				27.6-33.3		28 - 33		25.2-32.3	24.86-32.8	
		F		24.7 - 32.8		24.4 - 32.7				27.6-33.3		26.5 - 32.6		24-32.3	26.3-33.58	
		MF	**28.2 - 32.7**	24.3 - 33.2	27.0 - 31.0	24.8 - 32.8	25.2 - 32.2	23.2-31.8	27.5-33.2		26.9 - 32.3	27.4 - 32.8	25-32.5			20.81-31.2
MCHC	g/L	M		325 - 363		324 - 354				325-352		304 - 337		317-360	320.6-365	
		F		323 - 356		320 - 350				325-352		300 - 337		312-360	320.0-360	
		MF	**330 - 353**	325 - 358	300.0 - 380.0	322 -352	319 - 350[g]	290-352	317 - 353		319 - 353	302 - 338	314-361			31-37.2
RDW-SD	fL	M		36.5 - 45.9												
		F		37.6 - 50.3												
		MF	**37.7-45.5**	37.1 - 47.8							37.5 - 48.1					
RDW-CV	%	M	**12.2-14.2**	12.1 - 16.2		11.3 - 14.7		11.7-16.0		11.4-13.5		12.3 - 15.5	11.3-14.1		12.46-17.56	
		F	**12.3-15.0**	12.3 - 17.7		11.4 - 15.8		12.0-17.3		11.4-13.5		12.2 - 17	11.0-16.4		12.4-15.59	
		MF		12.3 - 17.0	11.5 - 14.5	11.3 - 15.2	12.2 - 16.3[g,h]				12 - 14.8	12.3 - 15.6				11.1-17.8
Ret	x10^9/L	M		18.8 - 108.6	10.0-90.0e											
		F		23.0 - 93.5	10.0-90.0[e]											
		MF	**23.0 - 84.1**	22.1 - 96.3												
Ret%	%	M		0.42-2.23	0.24-1.67											
		F		0.51 - 2.17	0.12-2.05[e]											
		MF	**0.54-1.77**	0.5 - 2.17	0.2 - 1.2[e]						0.4-1.6					
MRV	fL	M		97.5 - 122.7	101.1-128.8[e]											
		F		96.4 - 118	101.4-125.0[e]											
		MF	**97.6-117.8**	97.4 - 120.2							93.9-124.1					
IRF	%	M		30 - 54	19-41[e]											
		F		26 - 52	17-40[e]											
		MF	**23.5-49.8**	29 - 53												

Analyte	Unit	Sex	Russian study[a]	Beckman IFU[a]	Russian Methodological guidelines[d,e] (33,34)	Kenyan study[f] (7)	Turkish study[g,h,i] (6)	Ghanian study[j] (8,31)	Japanese study[a,o] (5)	Canadian study CALIPER[m] (26)	Malaysian study[a,o] (27)	Erithea (Asmara), Africa[g] (28)	Saudi Arabian study[i] (9)	Moroccan study[r] (29)	Ethiopian study[s] (32)	Omani study[i,t] (30)
WBC	x10^3/µL	M		3.6 - 10.2		3.13 - 8.1			3.3 - 8.6	3.8-10.4		3.7 - 9.3		4.1-10.8	3.31-11.62	
		F		3.8 - 11.8		2.89 - 7.72				3.8-10.4		3.3 - 8.9		4.1-10.7	3.24-10.05	
		MF	**3.54-9.21**	3.6 - 11.2		3.08 - 7.83	4.39 - 11.59	3.08-7.53			4.078-11.37	3.4 - 9.0	3.38-11.31			2.79-8.09
Neu	x10^3/µL	M		1.7 - 7.6		1.02 - 3.92				Age17-50:1.8-7.2<br>Age51-79:2.0-6.4				1.8-7	1.01-7.22	
		F		1.9 - 8.2		1.07 - 4.42				Age17-50:2.0-7.4<br>Age51-79:2.0-6.4				1.8-7	1.08-6.69	
		MF	**1.67-5.85**	1.8 - 7.8	2.0 - 5.5	1.05 - 4.08	2.04 - 7.54	1.17-3.81			3.929 - 7.147		1.21-6.81			0.91-4.61
Eos	x10^3/µL	M	**0.05-0.59**	0.0 - 0.5		0.05 - 0.64				0.1-0.2			0.05-0.64	0-0.6	0.05-1.21	
		F	**0.041-0.38**	0.0 - 0.5		0.04 - 0.49				0.1-0.2			0.04-0.51	0-0.5	0.04-1.12	
		MF		0 - 0.5	0.02 - 0.3	0.04 - 0.59	0.02 - 0.5	0.02-0.29			0 - 0.827					0.03-0.37
Bas	x10^3/µL	M		0.0 - 0.1		0.01 - 0.08				0.0-0.1			0.01-0.14	0-0.08	0.01-0.05	
		F		0.0 - 0.1		0.01 - 0.06				0.0-0.1			0.01-0.11	0-0.08	0.0-0.05	
		MF	**0.011-0.092**	0.0 - 0.1	0.0 -0.065	0.01 - 0.07	0.0 - 0.09[g]	0.01-0.08			0 - 0.095					0.002-0.058
Mon	x10^3/µL	M	**0.31-0.88**	0.3 - 1.1		0.15 - 0.76				Age6-44:0.2-0.8<br>Age45-79:0.3-0.9				0.2-1.2	0.24-0.88	
		F	**0.27-0.73**	0.2 - 0.9		0.14 - 0.68				Age6-44:0.2-0.8<br>Age45-79:0.2-0.8				0.2-1.2	0.27-0.87	
		MF		0.3 - 1.0	0.09 - 0.6	0.14 - 0.74	0.26 - 0.94	0.22-0.73			0.385 - 1.141		0.26-0.83			0.23-0.72
Lym	x10^3/µL	M	**1.23-3.55**	1.0 - 3.2		1.36 - 3.58				1.0-3.2				1.2-3.8	1.1-3.84	
		F	**1.13-3.17**	1.1 - 3.1		1.22 - 3.24				1.0-3.2				1.2-3.8	1.2-3.98	
		MF		1.0 - 3.0	1.2 - 3.0	1.29 - 3.4	1.21 - 3.77	1.22-3.38			1.847 - 4.807		1.31-4.12			1.12-3.15
Neu%	%	M		43.5 - 73.5		27.4 - 60.3										
		F		42.7 - 76.8		29.5 - 65.4										
		MF	**38.8-70.1**	43.3 - 76.6	47.0 - 72.0	28.0 - 63.3	40 - 74	29-62					27.4-68.5			
Eos%	%	M	**0.8-8.66**	0.8 - 8.1		1.2 - 11.8		0.5-10.3					0.89-8.54			
		F	**0.75-6.56**	0.5 - 7.0		0.8 - 9.4		0.4-6.5					0.52-7.21			
		MF		0.6 - 7.9	0.5 - 5.0	1.1 - 11.9	0 - 6									
Bas%	%	M		0.2 - 1.5		0.4 - 1.2										
		F		0.2 - 1.3		0.3 - 1.0										
		MF	**0.21-1.51**	0.2 - 1.4	0.1 - 1.0	0.3 - 1.0	0.1 - 1.0[g]	0.2-1.5					0.09-1.59			
Mon%	%	M	**5.93-12.42**	5.5 - 13.7		3.5 - 14.3					3.1 - 11.6					
		F	**4.91-11.29**	4.3 - 10.9		3.2 - 11.0					3 - 11.8					
		MF		4.5 - 12.5	3.0 - 11.0	3.4 - 13.3	4.0 - 12	5.0-13.7			3.1 - 11.7					
Lym%	%	M		15.2 - 43.3		28.2 - 60.3					22 - 59.9					
		F		16.0 - 45.9		25.5 - 59.3					22.3 - 58.2					
		MF	**20.6-47.9**	16.0 - 43.5	19.0 - 37.0	27.2 - 60.0	17 - 47	27-60			22 - 59.2		23-58.4			

Analyte	Unit	Sex	Russian study[a]	Beckman IFU[a]	Russian Methodological guidelines[d,e] (33,34)	Kenyan study[f] (7)	Turkish study[g,h,i] (6)	Ghanian study[j] (8,31)	Japanese study[a,o] (5)	Canadian study CALIPER[m] (26)	Malaysian study[a,o] (27)	Erithea (Asmara), Africa[g] (28)	Saudi Arabian study[i] (9)	Moroccan study[r] (29)	Ethiopian study[s] (32)	Omani study[i,t] (30)
PLT	x10^3/µL	M	**132-288**	152.0 - 348.0		133 - 356		115-339		Age14-26: 138.7-319.6<br>Age27-79: 151.8-324	142 - 350	128.4-318.4	165-380	145-338	164.0-403.4	146-347
		F	**135-330**	179.0 - 408.0		152 - 443		157-402		Age14-26: 158.1-361.6<br>Age27-79: 153.2-361.3	171 - 399	145.4-351.6	160-443	150-378	202.3-444.5	164-368
		MF		159 - 386	180 - 320	144 - 409	152 - 383		158 - 348			134-344.2				
MPV	fL	M		7.4 - 11.4						7.0-10.3			6.36-10.01	9.4-13.7		
		F		7.9 - 10.8						7.0-10.3			6.34-11.0	9-13.7		
		MF	**7.41-11.8**	7.5 - 11.2	7.4 - 10.4		7.0 - 11.8[g]				8.9-11.9					6.61-10.3
PDW, RUO	%	MF	**16.0 - 17.9**										14.9-17.9			7.7-17.4

Analyte	Unit	Sex	Russian study[b,c]	Beckman IFU[b,c]	Kenyan study[e] (7)	Turkish study (6)	Ghanian study[k] (8,31)	Japanese study, Asian study[c,l] (4,5)	Chinese study[b] (10)	Indian study[u] (11)	Saudi Arabian study[p] (9)
Fe	µmol/L	M	9.8-35.1	12.5 - 32.2		5.9 - 31.6	8.6 - 28.5	8.0 - 34.0[h,6]	10.0-34.0	7.0-33.0	9.0-27.4
		F	7.2-30.5	10.7 - 32.2		3.5 - 27.8	5.5 - 22.4	4.0 - 32.0	5.0-30.0	4.0-26.0	4.3-25.6
		MF			8.8 - 28.9			5.0 - 33.0			
UIBC	µmol/L	M	18.7-54.1			21.5 - 64.7		19-55[6]			23.6-51.2
		F	26.2-63.5			28.3 - 78.1		24-75			29.2-71.9
		MF		27.8 - 63.6				21-69			
TIBC	µmol/L	M	46.0-71.4	N/A		44.0 - 82.2					
		F	47.0-78.4			46.8 - 88.9					
TF	g/L	M	1.88-3.16		1.9-3.2			1.99-3.18	1.83–3.02		1.99-3.11
		F	1.94-3.57		2.0-3.5			2.04-3.66	1.95–3.52		2.13-3.99
		MF		2.0 - 3.6				2.01-3.52			
TFRSat	%	M	16.6-64.3	N/A	15 - 49						
		F	11.5-51.3		10.0 - 44						
Ferr	µg/L	M	29.8-271[c]	23.9 - 336.2		13.0 - 276.0		24-268[i]			
		F	<45 yo: 5.5-67<br>>45 yo: 5.3-223	11. 0 - 306.8		<45 yo: 4.3 - 91<br>>45 yo: 5.9 - 175		3-121			
VitB12	pmol/L	M	113-549[c]					91 - 520[6]			
		F	125-710					112 - 606			
		MF		133 - 675[1]				100 - 575			
		MF		107 - 133[2]							
		MF		<107[3]							
Folate	ng/mL	M	3.15-16.5[c]					8.7-34.7[i]			
		F	3.58-21.1					10.8-46			
		MF		5.9 - >24.8[4]				9.4-40			
		MF		3.1 - 19.9[5]							

1 -Normal range; 2 -Indeterminate Range; 3 - Deficiency range; 4 - USA population; 5 - British population; 6 - all countries from Asian study

a) UniCel DxH 800 (Beckman Coulter Inc., USA); b) AU 5800 (Beckman Coulter Inc., USA); c) UniCel DxI (Beckman Coulter Inc., USA); d) Manual method (Goryaev's chamber); e) Gen-S (Beckman Coulter Inc., USA); f) AST 5 Diff (Beckman Coulter Inc., USA); g) LH 780 (Beckman Coulter Inc, USA); h) XT-2000i (Sysmex Corporation, Japan); i) Cell-Dyn 3700 Sapphire (Abbott Laboratories, USA); j) XN 1000 (Sysmex Corporation, Japan); k) AU 480 (Beckman Coulter Inc., USA); l) UniCel DxC (Beckman Coulter Inc.); m) HmX analyzer (Beckman Coulter Inc.); o) XE 5000 (Sysmex Corporation, USA); p) Architect 16000c (Abbott Laboratories, USA); r) KX21N (Sysmex Corporation, Japan); s) XS 500i (Sysmex Corporation, Japan); t) XS-1000i (Sysmex Corporation, Japan); u) DxC 800 (Beckman Coulter Inc., USA)

Fig. 4 A Between-country comparison of RIs for erythrocyte and iron markers

Fig. 4 B Between-country comparison of RIs for leukocyte parameters

Russia* - indicates RIs from the current Russian study; Russia (without *) –indicates RIs from the Russian Methodological Guidelines [33,34]. The back shades indicate Russian RIs for male (blue), female (pink), and both genders (purple).

2.2 Platelet counts

LLs of PLT in the current Russian study (male: 132; female:135×10^3/µL) were significantly lower compared to the LLs shown in the IFU (152; 179×10^3/µL) [43] and LLs in the Russian Methodological guidelines for interpretation of blood tests (180×10^3/µL for both sexes) [33]. In fact, the Russian RIs for PLT were the lowest among all countries examined, as shown in **Table 4** [4-11, 26-32] and **Fig. 4**. Although the PLT RI from the Russian Methodological guidelines was recently revised to 150−400 ×10^3/µL, RIs from this study are still shifted to a lower side: 132−288 (male) and 135−330 (female) ×10^3/µL. The reason for this difference is unknown. Hence it certainly requires further investigation by inclusion of more volunteers from Caucasian population. Noticeably, that in most studies female RIs for PLT are shifted to a higher side, which can be explained by the female predominance of iron deficiency, which tends to cause increase of thrombopoiesis [44,45].

2.3 Vitamin B12 and folate

The recommended laboratory evaluation for patients with suspected macrocytic anemia includes CBC, VitB12, and folate levels assessment. The concentration of plasma vitamin B12 suggested by WHO for defining vitamin B12 deficiency is < 150 pmol/L, whereas the American Academy of Family Physicians (AAFP) and RNHS suggest <111 and <103 pmol/L respectively, which corresponds to the cutoff provided in the IFU (107 pmol/L) [38-40,46]. Our LLs for VitB12 (male 113 and female 125 pmol/L) are above the AAFP, and RNHS cutoffs, and the LL reported in the Japanese study (91 and 112 pmol/L for males and females respectively), but noticeably lower than the WHO cutoff and the IFU LL of a "normal range" (133 pmol/L) **(Table 4, Fig 4A)** [5, 38, 46]. Folate LLs of Russian males and females (3.15 and 3.58 ng/mL) were a little lower than those recommended by WHO (< 4 ng/mL) and provided in the IFU for the USA population (5.9 ng/mL), but comparable to the IFU cutoff derived for the British population (3.1 ng/mL), and again much lower than the LLs reported in the Japanese study for males and females (8.7 and 10.8 ng/mL, respectively), that could be a result of an insufficient dietary intake **(Table 4, Fig 4B)** [5,38,47].

2.4 Leukocyte counts

Significant between-country differences were observed for RI limits of Neu count, with LLs being conspicuously low in Oman, Kenya, Ghana, and Ethiopia [7,8,30,32]. For black African population, Omuse et al. speculated that the low RIs for Neu count may be associated with the DARC-null genotype, an evolutionary adaptation that is thought to make Africans less susceptible to Plasmodium vivax infections [7]. Another factor could be benign ethnic neutropenia, a common form of neutropenia worldwide that affects African and Middle Eastern ethnicities, especially people living at high altitude [48].

RIs limits for both sexes for Eos in the Russian study were comparable with limits from Kenya, Ghana, Saudi Arabia, Morocco, Ethiopia, and Omani, and with those provided in the IFU [7-9,29,30,32,43]. Omuse et al., observed much lower RIs for Eos in comparison to other African studies, and attributed it to the recruitment of the urban population with a lower risk of parasitic infection [7].

The UL for Mon counts in Russia was prominently low, but comparable to RIs of the Russian Methodological guidelines [33], and to those of the Kenyan, Omani, and Ghanian studies [7,8,30].

Meanwhile, RI limits for Lym count are comparable across countries except Malaysia, which showed elevated RIs for WBC and all differential counts [27] of unknown epidemiological background.

Conclusion

With the participation of 506 healthy Russian adults, we investigated sources of variation and determined RIs parametrically for hematology parameters, including related iron markers and vitamins.

Gender-difference requiring separate RIs for each gender were apparent in most of the analytes, while age-related changes in RVs were noted only for ferritin in females. BMI-related increases in RVs were observed prominently for reticulocyte parameters, which required exclusion of individuals with BMI>28 kg/m^2 before determining the RIs. The LAVE method was found effective in excluding individuals with latent anemia without inclusion of iron makers in the reference tests.

International comparison revealed that Russian RIs featured a lower side shift of platelet counts. Besides, as in African countries, Russian RIs for total leukocyte and neutrophil counts were lower compared to most of other countries. The observed ethnic differences will require further investigation for validation.

Acknowledgement

The authors express sincere gratitude to Beckman Coulter, LLC (Russia) for their generous support of the assay reagents. We are grateful to Yury Andreychuk, CEO of Helix Laboratories Services, and the Helix staff for their kind assistance in the recruitment of volunteers, sample preparations, and provision of sampling equipment.

References:

1) IFCC and CLSI. EP28-A3c document. Defining, establishing, and verifying reference intervals in the clinical laboratory: approved guideline, 2010; 3rd ed., vol. 28 No. 30.

2) Ceriotti C, Hinzmann F, Panteghini R. Reference intervals: the way forward. Annals of clinical biochemistry. 2007; 46:8-17. https://doi: 10.1258/acb.2008.008170

3) Ozarda Y, Ichihara K, Barth JH, et al. Protocol and standard operating procedures for common use in a worldwide multicenter study on reference values. Clin Chem Lab Med. 2013; 51:1027–40. https://doi: 10.1177/2050312118807626

4) Ichihara K, Ceriotti F, Tam TH et al. The Asian project for collaborative derivation of reference intervals: (2) results of nonstandardized analytes and transference of reference intervals to the participating laboratories on the basis of cross-comparison of test results. Clin Chem Lab Med. 2013. https//doi 10.1515/cclm-2012-0422

5) Ichihara K, Yomamoto, Y. Hotta T et al. Collaborative derivation of reference intervals for major clinical laboratory tests in Japan. Annals of Clinical Biochemistry. 2016; Vol. 53(3) 347–356. https//doi: 10.1177/0004563215608875

6) Ozarda Y, Ichihara K, Bakan E et al. A nationwide multicentre study in Turkey for establishing reference intervals of haematological parameters with novel use of a panel of whole blood. Biochem Med. 2017; 27:350-377. https://doi: 10.11613/BM.2017.038

7) Omuse G, Maina D, Mwangi J et al. Complete blood count reference intervals from a healthy adult urban population in Kenya. PLoS One. 2018;13(6): e0198444. https://doi.org/10.1371/journal.pone.0198444

8) Bawua A., Ichihara K, Keatley R et al. Establishing Ghanaian adult reference intervals for hematological parameters controlling for latent anemia and inflammation. Int J Lab Hematol, 2020; 42(6): 705–717. https://doi: 10.1111/ijlh.13296

9) Borai A, Ichihara K, Bahijri S. et al. Establishment of reference intervals for hematological parameters of adult population in the western region of Saudi Arabia. PLoS ONE. 2023, 18(2): e0281494. https://doi.org/10.1371/journal.pone.0281494

10) Xia L, Chen M, Liu M et al. Nationwide Multicenter Reference Interval Study for 28 Common Biochemical Analytes in China. Medicine. 2016; Vol. 95, 9. https://doi: 10.1097/MD.0000000000002915

11) Shah SAV, Ichihara K, Dherai AJ et al. Reference intervals for 33 biochemical analytes in healthy Indian population: C-RIDL IFCC initiative. Clin Chem Lab Med. 2018; 56(12): 2093–2103. https:// doi: 10.1515/cclm-2018-0152

12) Evgina S, Ichihara K, Ruzhanskaya A et al. Establishing reference intervals for major biochemical analytes for the Russian population: a research conducted as a part of the IFCC global study on reference values. Clin Biochem. 2020; 81:47–58. https:// 10.1016/j.clinbiochem.2020.04.001

13) Ruzhanskaya A, Ichihara K, Evgina S et al. Sources of variation and establishment of Russian reference intervals for major hormones and tumor markers. Plos One, 2021; https://doi.org/10.1371/journal.pone.0234284

14) Ichihara K. Statistical considerations for harmonization of the global multicenter study on reference values. Clin Chim Acta. 2014; 432:108–18. https://doi.org/10.1016/j.cca.2014.01.025 PMID:24518360

15) Ichihara K, Ozarda Y, Barth JH et al. et al. A global multicenter study on reference values: 2. Exploration of sources of variation across the countries. Clin Chim Acta. 2017; 467: 83–97. https://doi.org/10.1016/j.cca.2016.09.015 PMID: 27666762

16) Cohen J. A power primer. Psychological Bulletin. 1992; 112: 155–9. https://doi.org/10.1037//0033-2909.112.1.155 PMID: 19565683

17) Fraser CG. Biological variation: from principles to practice. AACC Press. Copyright 2001; 29–66.

18) Access Ferritin, Instructions for Use C64647 A, December 2021

19) AU Iron, Instructions for Use BLOSR6X86 08, May 2021

20) Ma A, Jia R, Ma X et al. Iron Storage in Women is Positively Correlated with Aging and BMI Values. FASEB. 2017; vol. 30, Iss.3. https://doi.org/10.1096/fasebj.30.1_supplement.lb377

21) Pagani A, Nai A, Silvestri L et al. Hepcidin and Anemia: A Tight Relationship. Front. Physiol. 10:1294. doi: 10.3389/fphys.2019.01294

22) Pérez OM, López MA, Alcalde IM et al. Relationship between Obesity and Iron Deficiency in Healthy Adolescents. Child Obes. 2020 Sep;16(6):440-447. https://doi: 10.1089/chi.2019.0276

23) Jeong HR, Lee HS, Shim YS et al. Positive Associations between Body Mass Index and Hematological Parameters, Including RBCs, WBCs, and Platelet Counts, in Korean Children and Adolescents. Children 2022, 9, 109. https://doi.org/10.3390/children9010109

24) He S, Gu H, Yang J et al. Hemoglobin concentration is associated with the incidence of metabolic syndrome. BMC Endocr Disord. 2021; 21, 53. https://doi.org/10.1186/s12902-021-00719-4

25) Shimizu Y, Kawashiri SY, Yamanashi H et al. Reticulocyte levels have an ambivalent association with hypertension and atherosclerosis in the elderly: a cross-sectional study. Clin Interv Aging. 2019; 14:849-857. https://doi: 10.2147/CIA.S197982.

26) Adeli K, Raizman JE, Chen Y et al. Complex biological profile of hematologic markers across pediatric, adult, and geriatric ages: establishment of robust pediatric and adult reference intervals on the basis of the Canadian Health Measures Survey. Clin Chem. 2015, Aug;61(8):1075-86. https//doi: 10.1373/clinchem.2015.240531. Epub 2015 Jun 4. PMID: 26044509

27) Ambayya A, Su AT, Osman NH et al. Haematological Reference Intervals in a Multiethnic Population. PLOS ONE. 2014; 9(3): e91968. https://doi.org/10.1371/journal.pone.0091968

28) Siraj N, Issac J, Anwar M et al. Establishment of hematological reference intervals for healthy adults in Asmara. Siraj et al. BMC Res Notes. 2018; 11:55. https://doi.org/10.1186/s13104-018-3142-y

29) Bakrim S, Motiaa Y, Benajiba M et al. Establishment of the hematology reference intervals in a healthy population of adults in the Northwest of Morocco (Tangier-Tetouan region). Pan Afr Med J. 2018; Mar 23;29:169. https//doi: 10.11604/pamj.2018.29.169.13042. PMID: 30050633; PMCID: PMC6057597

30) Al-Mawali A, Pinto AD Al-Busaidi R. et al. Comprehensive haematological indices reference intervals for a healthy Omani population: First comprehensive study in Gulf Cooperation Council (GCC) and Middle Eastern countries based on age, gender and ABO blood group comparison. PLoS One. 2018; Apr 5;13(4):e0194497. https//doi: 10.1371/journal.pone.0194497. PMID: 29621271; PMCID: PMC5886408

31) Abbam G, Tandoh S, Tetteh M et al. Reference intervals for selected hematological and biochemical parameters among apparently healthy adults in different eco-geographical zones in Ghana. PLoS One. 2021; 16(1): e0245585. https://doi.org/10.1371/journal.pone.0245585

32) Bimerew LG, Demie T, Eskinder K Reference intervals for hematology test parameters from apparently healthy individuals in southwest Ethiopia. SAGE Open Medicine. 2018; Vol. 6: 1–10. https://doi: 10.1177/2050312118807626

33) Луговская СА, Почтарь МЕ, Долгов ВВ. Гематологические анализаторы. Интерпретация анализа крови. Методические рекомендации. Кафедра КЛД, 2007/ Lugovskaya SA, Pochtar ME, Dolgov VV. Hematological analyzers. Interpretation of the blood test. Russian Methodological guidelines. Department of Clinical Laboratory Diagnostics, Russian Medical Academy of Postgraduate Education, 2007

34) Луговская СА, Почтарь МЕ, Ретикулоциты. Москва, Российская Медицинская академия постдипломного образования. 2006/ Lugovskaya SA, Pochtar ME. Reticulocytes. Russian Medical Academy of Postgraduate Education. 2006

35) Serum ferritin concentrations for the assessment of iron status in individuals and populations: technical brief. WHO, ISBN 978-92-4-000852-6 (electronic version). 2020 https://www.who.int/publications-detail-redirect/9789240008526

36) Iron Deficiency Anemia: Guidelines from the American Gastroenterological Association AGA Clinical Practice Guidelines on the Gastrointestinal Evaluation of Iron Deficiency Anemia. Am Fam Physician. 2021;104(2):211-212

37) Клинические рекомендации МЗ РФ, Железодефицитная анемия, 2021 / Clinical recommendations of the Ministry of Health of the Russian Federation, Russian National Hematology Society. Iron deficiency anemia, 2021

38) de Benoist B. Conclusions of a WHO technical consultation on folate and vitamin B12 deficiencies. Food Nutr Bull 2008;29(Suppl 2): S238– 44

39) Langan R, Goodbred A. Vitamin B12 Deficiency: Recognition and Management. Vol. 96, 6 American Family Physician, 2017. www.aafp.org/afp

40) Клинические рекомендации МЗ РФ. Витамин В12 дефицитная анемия. 2021 / Clinical guidelines of the Ministry of Health of the Russian Federation, Russian National Hematology Society. Vitamin B12 deficiency anemia. 2021

41) Muckenthaler MU, Mairbäurl H, Gassmann M. Iron metabolism in high-altitude residents. J Appl Physiol 129: 920–925, 2020. doi:10.1152/japplphysiol.00019.2020

42) https://www.who.int/data/gho/data/themes/topics/anaemia_in_women_and_children

43) UniCel DxH Series with System Manager Software, PN B26647AE. January 2017

44) Choi SI, Simone JV. Platelet Production in Experimental Iron Deficiency Anemia. Blood. Volume 42, Iss. 2, August 1973, Pages 219-228

45) Evstatiev R, Bukaty A, Jimenez K et al. Iron deficiency alters megakaryopoiesis and platelet phenotype independent of thrombopoietin. Am J Hematol. 2014 (5):524-9. doi: 10.1002/ajh.23682

46) Access Vitamin B12 Cobalamin, Instruction for Use, A89094 M, October 2022

47) Access Folate; Instruction for Use, B03898 L, April 2020

48) Alkhaldy H, Awan Z, Abouzaid A et al. Prevalence of Isolated Neutropenia at High Altitude in Southern Saudi Arabia: Does Altitude Affect Leucocyte Count? Int J Gen Med. 2020; 13:1373-1379. https//doi: 10.2147/IJGM.S284133. PMID: 33299343; PMCID: PMC7721288

Figure Legends

Fig 1 Between-gender differences for major parameters

Gender-specific distributions of RVs for major parameters are drawn by scattergrams. The background shades represent RIs determined for male (blue), female (pink), and for both (purple). The central 50% range and median of RVs are shown by the horizontal box and the vertical line.

Fig 2 Hematology RVs influenced by BMI and age

RVs of RBC, Ret, Ret%, and IFR were partitioned by gender and BMI into five groups, while RVs were partitioned by age at 45 years. The central 50% range and median of RVs are shown by the horizontal box and the vertical line. No secondary exclusion was done while drawing the scattergrams.

Fig 3 The effect of LAVE on RIs for hematology tests in comparison to IDA criteria

Female RIs of 6 representative hematology tests were determined parametrically using the following 4 sets of RV datasets.

1: All: RVs without any prior exclusion

2: LAVE(-) 7RT: RVs with no abnormal value among 7 tests (Hb, MCH, MCHC, RDW-CV, Ret, Fe, Ferr) other than itself

4: LAVE(-) 5RT: RVs with no abnormal value among 5 ref tests (Hb, MCH, MCHC, RDW-CV, Ret) other than itself

6: IDA(-): RVs with no abnormal results for Fe and Ferr from respective lower limit of the manufacturer's RI.

Each bar shown over the scattergram represents the span of RI with its lower and upper limits indicated by numerical labels. The three scattergrams of RVs shown in red dots in the 3rd, 5th, and 7th rows represent values that were excluded by the criteria applied to obtain RVs shown above in the 2nd, 4th, and 6th rows in green. Thus, their group labels were designated as follows:
As a reference, the following three datasets were prepared and plotted in the 3rd, 5th , and 7th row.

3: LAVE(+) 7RT: RVs with one or more abnormal values among 7 ref tests (Hb, MCH, MCHC, RDW-CV, Ret, Fe, Ferr)

5: LAVE(+) 5RT: RVs with one or more abnormal values among 5 ref tests (Hb, MCH, MCHC, RDW-CV, Ret)

7: IDA(+): RVs had abnormal results either for Fe or Ferr.

Fig 4 A Between-country comparison of RIs for erythrocyte and iron markers

Fig 4 B Between-country comparison of RIs for leukocyte parameters

Russia* - indicates RIs from the current Russian study; Russia (without *) –indicates RIs from the Russian Methodological Guidelines [33,34]. The back shades indicate Russian RIs for male (blue), female (pink), and both genders (purple).

Suppl Fig 1 Bar-chart comparison of LAVE effect for RBC & AAM

Supplemental Tables

Suppl Table 1 Assay methods and traceability

Analyte	Abbr	Method	Analyzer	Reagent	Tracebility	Unit
Iron	Fe	Photometric colour test	DxC 5800 AU	Beckman Coulter	Beckman Coulter Master Calibrator	µmol/L
Unsaturated iron binding capacity	UIBC	Photometric colour test	DxC 5800 AU	Beckman Coulter	NIST SRM 937	µmol/L
Transferrin	TF	Immuno-turbidimetric test	DxC 5800 AU	Beckman Coulter	IFCC standard CRM 470	g/L
Ferritin	Ferr	Chemiluminescent immunoassay	UniCel DxI 800	Beckman Coulter	WHO 3rd IS 94/572	µg/L
Vitamin B12	Vit B12	Chemiluminescent immunoassay	UniCel DxI 800	Beckman Coulter	Manufacturer's working calibrator	pmol/L
Folate	Folate	Chemiluminescent immunoassay	UniCel DxI 800	Beckman Coulter	Manufacturer's working calibrator	µg/L
Red Blood Cells	RBC	Triple-aperture impedance cell counter	UniCel DxH 800	Beckman Coulter	Clin Lab Haemat, 1994, 16(2):131-138	10^6 /µL
Hemoglobin	Hb	Hemiglobincyanide spectrophotometric procedure, modified Drabkins (Ziljstra) Reagent is referenced to NIST certified filters	UniCel DxH 800	Beckman Coulter	CLSI Standard H15-A3	g/L
Mean corpuscular volume	MCV	PCV/RBC x 10, CLSI Standard H7-A3.2	UniCel DxH 800	Beckman Coulter	CLSI Standard H7-A3	fL
Mean corpuscular hemoglobin	MCH	N/A	UniCel DxH 800	Beckman Coulter	N/A	pg
Mean corpuscular hemoglobin concentration	MCHC	N/A	UniCel DxH 800	Beckman Coulter	N/A	g/L
Red cell distribution width - standard deviation	RDW-SD		UniCel DxH 800	Beckman Coulter	N/A	fL
Reticolocytes	Ret		UniCel DxH 800	Beckman Coulter	Accuracy is assessed by comparison of results from DxH and a comparative method. Results may be compared to either a comparator hematology analyzer or to a 400 cell manual differential prepared according to CLSI H20-A2	10^9/L
Mean reticulocyte volume	MRV		UniCel DxH 800	Beckman Coulter	N/A	fL
Immature reticulocyte fraction	IRF		UniCel DxH 800	Beckman Coulter	N/A	_
White blood cells	WBC	Triple-aperture impedance cell counter	UniCel DxH 800	Beckman Coulter	Clin Lab Haemat, 1994, 16(2):131-138	10^3 /µL
Neutrphiles	Neu		UniCel DxH 800	Beckman Coulter	Accusracy for the differential parameters is assessed by comparison of results from DxH and a comparator hematoogy instrument using CLSI EP09-A3	10^3 /µL
Eosinophiles	Eos		UniCel DxH 800	Beckman Coulter		10^3 /µL
Basophiles	Bas		UniCel DxH 800	Beckman Coulter		10^3 /µL
Monocytes	Mon		UniCel DxH 800	Beckman Coulter		10^3 /µL
Lymphocytes	Lym		UniCel DxH 800	Beckman Coulter		10^3 /µL
Platelets	PLT	Phase contrast microscopy	UniCel DxH 800	Beckman Coulter	Phase Contrast Microscopy	10^3 /µL
Mean platelet volume	MPV	Latex particles	UniCel DxH 800	Beckman Coulter	Latex Particles (PN 7531820)	fL

Suppl Table 2 Assessment of reference tests for use in the LAVE method

(A) Correlation matrix among parameters of leukocyte and inflammatory markers

	TF	Ferr	Hb	WBC	Neu	Eos	Bas	Mon	Lym	PLT
**TF**	----	**-0.412**	**-0.269**	0.021	0.061	-0.090	0.028	-0.002	0.003	0.213
**Ferr**	**-0.412**	----	**0.647**	0.121	0.072	0.129	-0.073	0.193	0.149	**-0.251**
**Hb**	**-0.269**	**0.647**	----	0.216	0.129	0.166	0.006	**0.305**	0.218	-0.207
**WBC**	0.021	0.121	0.216	----	**0.879**	**0.326**	**0.339**	**0.664**	**0.620**	0.174
**Neu**	0.061	0.072	0.129	**0.879**	----	0.159	**0.256**	**0.510**	**0.270**	0.167
**Eos**	-0.090	0.129	0.166	**0.326**	0.159	----	**0.329**	**0.265**	**0.326**	0.070
**Bas**	0.028	-0.073	0.006	**0.339**	**0.256**	**0.329**	----	0.227	**0.291**	0.130
**Mon**	-0.002	0.193	**0.305**	**0.664**	**0.510**	**0.265**	0.227	----	**0.458**	0.132
**Lym**	0.003	0.149	0.218	**0.620**	**0.270**	**0.326**	**0.291**	**0.458**	----	0.137
**PLT**	0.213	**-0.251**	-0.207	0.174	0.167	0.070	0.130	0.132	0.137	----

(B) Correlation matrix among parameters of erythrocyte and iron markers

	Fe	UIBC	Ferr	RBC	Hb	Ht	MCV	MCH	MCHC	RDW-CV	Ret	MRV	IRF
**Fe**	----	**-0.687**	**0.332**	0.169	**0.334**	**0.303**	0.241	**0.296**	**0.282**	**-0.297**	0.064	0.088	-0.002
**UIBC**	**-0.687**	----	**-0.442**	-0.096	**-0.319**	**-0.272**	**-0.323**	**-0.392**	**-0.352**	**0.368**	0.024	-0.103	0.025
**Ferr**	**0.332**	**-0.442**	----	**0.461**	**0.647**	**0.610**	**0.251**	**0.359**		**-0.335**	**0.322**	0.006	0.114
**RBC**	0.169	-0.096	**0.461**	----	**0.828**	**0.855**	**-0.259**	-0.165	0.147	0.017	**0.424**	**-0.253**	0.032
**Hb**	**0.334**	**-0.319**	**0.647**	**0.828**	----	**0.965**	0.205	**0.334**	**0.398**	-0.200	**0.410**	0.019	0.100
**Ht**	**0.303**	**-0.272**	**0.610**	**0.855**	**0.965**	----	0.210	0.246	0.221	-0.143	**0.398**	0.046	0.106
**MCV**	0.241	**-0.323**	**0.251**	**-0.259**	0.205	0.210	----	**0.878**	0.089	**-0.293**	-0.064	**0.627**	0.192
**MCH**	**0.296**	**-0.392**	**0.359**	-0.165	**0.334**	0.246	**0.878**	----	**0.459**	**-0.399**	0.036	**0.508**	0.165
**MCHC**	**0.282**	**-0.352**	**0.421**	0.147	**0.398**	0.221	0.089	**0.459**	----	**-0.452**	0.196	-0.095	-0.051
**RDW-CV**	**-0.297**	**0.368**	**-0.335**	0.017	-0.200	-0.143	**-0.293**	**-0.399**	**-0.452**	----	-0.048	0.027	0.129
**Ret**	0.064	0.024	**0.322**	**0.424**	**0.410**	**0.398**	-0.064	0.036	0.196	-0.048	----	-0.185	**0.419**
**MRV**	0.088	-0.103	0.006	**-0.253**	0.019	0.046	**0.627**	**0.508**	-0.095	0.027	-0.185	----	0.247
**IRF**	-0.002	0.025	0.114	0.032	0.100	0.106	0.192	0.165	-0.051	0.129	**0.419**	0.247	----

(B') Corr matrix of 7 reference tests chosen for LAVE

	Fe	Ferr	Hb	MCH	MCHC	RDW-CV	Ret
**Fe**	----	**0.332**	**0.334**	**0.296**	**0.282**	**-0.297**	0.064
**Ferr**	**0.332**	----	**0.647**	**0.359**		**-0.335**	**0.322**
**Hb**	**0.334**	**0.647**	----	**0.334**	**0.398**	-0.200	**0.410**
**MCH**	**0.296**	**0.359**	**0.334**	----	**0.459**	**-0.399**	0.036
**MCHC**	**0.282**	**0.421**	**0.398**	**0.459**	----	**-0.452**	0.196
**RDW-CV**	**-0.297**	**-0.335**	-0.200	**-0.399**	**-0.452**	----	-0.048
**Ret**	0.064	**0.322**	**0.410**	0.036	0.196	-0.048	----

7 reference tests, allowing no abnormal result among them, but LL and UL were extended by 3% of the RI width on both limits.

(B'') Corr matrix of 5 reference tests chosen for LAVE

	Hb	MCH	MCHC	RDW-CV	Ret
**Hb**	----	**0.334**	**0.398**	-0.200	**0.410**
**MCH**	**0.334**	----	**0.459**	**-0.399**	0.036
**MCHC**	**0.398**	**0.459**	----	**-0.452**	0.196
**RDW-CV**	-0.200	**-0.399**	**-0.452**	----	-0.048
**Ret**	**0.410**	0.036	0.196	-0.048	----

5 reference tests, allowing no abnormal result among them, but LL and UL were extended by 3% of the RI width on both limits.

Suppl Table 3 Full list of RIs for erythrocyte and iron markers partitioned by sex

Item	Unit	LAVE	Sex	N	90%CI of LL		LL	Me	UL	90%CI of UL		SDR_{sex}	BR<0.375		BR<0.57		decision
													LAVE effect		Gender diff		
													BR_{LL}	BR_{UU}	BR_{LL}	BR_{UU}	
RBC	10^6 /µL	(-)	MF	487	3.82	3.89	3.85	4.66	5.51	5.45	5.58	1.106	0.22	-0.14	1.14	0.94	Partition by sex, LAVE(-)
		(+)	MF	352	3.88	3.99	3.95	4.69	5.46	5.39	5.52				1.50	1.17	
		(-)	M	222	4.18	4.38	4.33	4.96	5.54	5.49	5.64		0.49	-0.03			
		(+)	M	177	4.43	4.52	4.48	4.96	5.53	5.48	5.61						
		(-)	F	261	3.77	3.88	3.85	4.40	5.14	5.07	5.23		0.18	-0.19			
		(+)	F	179	3.87	3.96	3.91	4.40	5.08	5.02	5.20						
Hb	g/L	(-)	MF	488	103	110	107	141	165	163	167	1.381	0.85	0.09	1.95	1.06	Partition by sex, LAVE(+) for female
		(+)	MF	361	116	120	119	142	166	164	167				1.65	1.29	
		(-)	M	223	127	135	133	151	166	164	167		0.33	0.07			
		(+)	M	181	134	138	136	151	166	165	168						
		(-)	F	261	101	109	104	131	150	148	152		1.02	0.08			
		(+)	F	185	113	118	116	133	151	148	153						
Ht	%	(-)	MF	485	32.1	33.7	32.8	41.2	48.0	47.9	48.7	1.369	0.62	-0.06	1.97	1.11	Partition by sex, LAVE(+) for female
		(+)	MF	352	34.7	35.8	35.2	41.7	47.7	47.4	48.2				1.66	1.31	
		(-)	M	222	38.6	40.0	39.5	43.9	48.5	48.2	49.3		0.24	-0.10			
		(+)	M	177	39.8	40.3	40.1	43.9	48.2	47.7	49.0						
		(-)	F	261	31.4	32.8	31.9	38.6	44.2	43.7	44.9		0.91	-0.04			
		(+)	F	178	34.0	35.0	34.8	39.0	44.1	43.5	44.6						
MCV	fL	(-)	MF	475	76.2	79.3	78.6	88.4	94.8	95.0	96.9	0.061	1.10	-0.04	2.14	0.49	Partition by sex, LAVE(+) for female
		(+)	MF	352	82.6	83.6	83.1	88.8	94.6	94.0	95.0				0.05	-0.06	
		(-)	M	224	81.4	82.8	82.3	88.6	96.5	95.6	98.4		0.10	-0.69			
		(+)	M	176	82.2	83.3	82.6	88.7	94.0	93.7	95.0						
		(-)	F	259	70.9	77.5	73.5	88.0	94.5	94.8	96.3		1.68	-0.06			
		(+)	F	179	81.9	83.2	82.5	88.5	94.2	93.7	94.8						
MCH	pg	(-)	MF	477	25.2	26.2	26.2	30.2	32.6	32.7	33.7	0.212	1.23	0.05	2.46	0.27	Actual difference in LL is small. No partiton by sex, LAVE(+)
		(+)	MF	364	27.8	28.2	28.2	30.3	32.7	32.5	32.9				0.56	0.48	
		(-)	M	222	27.1	28.1	27.8	30.4	32.9	32.6	34.2		0.34	-0.14			
		(+)	M	184	27.8	28.5	28.3	30.4	32.7	32.4	32.9						
		(-)	F	260	23.1	25.3	23.8	29.9	32.4	32.4	33.2		1.74	-0.13			
		(+)	F	185	26.9	28.0	27.6	30.0	32.2	32.1	32.9						
MCHC	g/L	(-)	MF	477	321	326	325	341	354	353	356	0.460	0.72	-0.11	1.41	0.45	Actual difference in LL is small. No partiton by sex, LAVE(+)
		(+)	MF	370	327	331	330	341	353	352	355				0.86	0.87	
		(-)	M	219	330	332	331	343	355	354	359		0.16	-0.07			
		(+)	M	180	330	333	332	343	354	353	357						
		(-)	F	258	317	322	321	339	352	351	355		0.81	-0.28			
		(+)	F	188	325	329	327	339	349	349	352						
RDW-SD	fL	(-)	MF	482	36.9	37.6	37.4	41.0	46.7	46.3	47.3	0.000	0.13	-0.51	-0.12	0.33	No partition by sex, LAVE(+)
		(+)	MF	351	37.5	37.9	37.7	40.9	45.5	45.1	46.0				-0.30	0.05	
		(-)	M	224	36.7	37.4	37.3	40.8	47.2	46.4	47.9		0.10	-0.65			
		(+)	M	177	37.3	37.8	37.5	40.7	45.5	44.8	46.4						
		(-)	F	258	37.0	38.0	37.6	41.2	46.4	45.9	47.1		0.24	-0.43			
		(+)	F	178	37.8	38.3	38.1	40.9	45.4	44.7	46.1						
RDW-CV	%	(-)	MF	482	12.1	12.3	12.3	13.2	15.7	15.3	16.3	0.256	0.04	-1.26	-0.09	-3.32	Partition by sex, LAVE(+) for female
		(+)	MF	373	12.2	12.3	12.3	13.1	14.6	14.4	15.0				-0.03	-1.41	
		(-)	M	222	12.0	12.2	12.2	13.1	14.2	14.2	14.5		0.12	-0.17			
		(+)	M	186	12.2	12.3	12.3	13.1	14.1	14.1	14.6						
		(-)	F	260	12.2	12.3	12.3	13.4	17.1	16.3	17.6		0.00	-1.75			
		(+)	F	189	12.2	12.4	12.3	13.2	15.0	14.9	15.5						
Ret	10^9/L	(-)	MF	395	23.4	25.9	24.9	50.6	94.6	91.2	100.4	0.339	0.03	-0.06	0.33	0.45	No partition by sex, LAVE(-)
		(+)	MF	299	23.5	26.4	25.4	51.2	93.5	88.3	96.3				0.49	0.67	
		(-)	M	161	26.7	31.9	29.5	56.1	97.0	90.5	110.8		0.09	0.12			
		(+)	M	133	27.9	34.3	31.1	56.7	99.1	91.3	107.3						
		(-)	F	232	21.8	24.2	23.6	46.9	89.0	87.4	96.4		-0.06	-0.09			
		(+)	F	168	21.3	24.8	22.7	46.5	87.4	81.8	91.5						
Ret%	%	(-)	MF	393	0.55	0.60	0.56	1.10	1.92	1.88	2.07	0.000	0.16	-0.07	0.19	0.35	No partition by sex, LAVE(-)
		(+)	MF	289	0.58	0.64	0.62	1.11	1.89	1.85	2.00				0.52	0.42	
		(-)	M	163	0.56	0.65	0.61	1.13	2.05	1.94	2.17		0.31	-0.13			
		(+)	M	127	0.69	0.76	0.72	1.15	2.00	1.87	2.19						
		(-)	F	232	0.52	0.58	0.55	1.07	1.93	1.86	2.09		0.02	-0.18			
		(+)	F	164	0.52	0.59	0.55	1.06	1.86	1.77	1.98						
MRV	fL	(-)	MF	392	96.9	98.2	97.6	106.8	117.8	117.1	119.3	0.000	0.11	-0.06	-0.01	0.06	No partition by sex, LAVE(-)
		(+)	MF	290	97.6	99.0	98.2	107.1	117.6	116.8	118.4				-0.04	-0.26	
		(-)	M	162	96.9	98.7	97.7	106.8	118.6	116.7	120.7		0.10	-0.44			
		(+)	M	127	97.3	99.2	98.2	106.8	116.3	114.6	117.8						
		(-)	F	232	96.6	98.3	97.7	106.8	118.3	117.2	119.2		0.13	-0.15			
		(+)	F	164	97.4	99.0	98.4	106.9	117.6	116.3	118.9						

**IRF**	–	(-)	MF	395	22.8	24.6	**23.9**	**36.8**	**49.1**	48.4	50.1	0.000	-0.08	-0.15	0.24	-0.08	No partition by sex, LAVE(-)
		(+)	MF	291	22.3	25.0	23.4	36.5	48.2	47.1	49.1				0.56	0.00	
		(-)	M	162	23.2	26.6	24.8	36.9	48.8	48.0	51.2		0.10	-0.15			
		(+)	M	127	23.1	27.5	25.4	36.9	47.9	46.5	49.6						
		(-)	F	233	21.6	24.5	23.3	36.8	49.3	48.1	50.6		-0.20	-0.22			
		(+)	F	164	20.7	23.6	21.9	36.2	47.9	46.3	49.3						
**Fe**	μmol/L	(-)	MF	483	5.4	6.7	6.3	17.3	32.4	31.8	34.7	**0.356**	0.29	0.16	**0.78**	**0.69**	Partition by sex, LAVE(+) for female
		(+)	MF	351	7.6	8.8	8.2	17.5	33.4	31.2	35.0				0.37	**0.82**	
		(-)	M	225	8.5	10.6	**9.8**	**18.8**	**35.1**	32.5	37.3		-0.03	0.10			
		(+)	M	176	8.3	10.3	9.6	18.9	35.8	33.2	37.6						
		(-)	F	260	3.9	5.9	4.6	15.7	30.5	30.1	34.0		**0.39**	-0.01			
		(+)	F	177	6.0	7.8	**7.2**	**16.4**	**30.5**	29.0	33.7						
**UIBC**	μmol/L	(-)	MF	418	19.4	22.7	22.4	40.8	66.9	64.2	68.9	**0.478**	-0.07	**-0.70**	**-0.62**	**-1.75**	Partition by sex, LAVE(+) for female
		(+)	MF	313	19.6	23.2	21.6	40.2	58.9	57.0	61.5				**-0.81**	**-0.91**	
		(-)	M	195	17.4	21.6	**18.7**	**37.8**	**54.1**	52.7	57.5		-0.03	0.08			
		(+)	M	157	16.5	21.5	18.4	37.9	54.8	53.2	58.6						
		(-)	F	224	20.8	25.3	25.8	44.0	74.0	68.8	76.0		0.03	**-0.85**			
		(+)	F	155	22.8	27.9	**26.2**	**42.9**	**63.5**	60.2	65.8						
**TIBC**	μmol/L	(-)	MF	418	45.4	47.0	47.4	58.7	79.2	76.6	80.1	**0.338**	-0.09	**-0.75**	-0.05	**-1.40**	Partition by sex, LAVE(+)
		(+)	MF	311	45.4	46.8	46.7	58.2	73.1	71.9	76.1				-0.13	**-1.04**	
		(-)	M	194	44.5	47.0	46.8	57.0	71.2	68.7	72.3		-0.13	0.03			
		(+)	M	157	44.2	46.8	**46.0**	**57.3**	**71.4**	69.2	73.6						
		(-)	F	225	45.5	48.1	47.3	60.8	82.6	79.6	85.3		-0.03	**-0.46**			
		(+)	F	155	44.9	47.8	**47.0**	**59.6**	**78.4**	75.5	81.0						
**TF**	g/L	(-)	MF	485	1.84	1.93	1.93	2.53	3.65	3.53	3.74	**0.426**	-0.01	**-0.74**	-0.17	**-1.57**	Partition by sex, LAVE(+) for female
		(+)	MF	349	1.84	1.95	1.92	2.49	3.32	3.24	3.39				-0.20	**-1.18**	
		(-)	M	225	1.80	1.92	**1.88**	**2.42**	**3.16**	3.07	3.22		-0.03	-0.05			
		(+)	M	176	1.80	1.92	1.87	2.42	3.14	3.05	3.24						
		(-)	F	261	1.86	2.00	1.95	2.66	3.85	3.73	3.97		-0.03	**-0.59**			
		(+)	F	178	1.85	1.98	**1.94**	**2.59**	**3.57**	3.43	3.70						
**TRFSat**	%	(-)	MF	418	8.0	10.5	9.4	29.8	57.1	55.9	60.2	**0.429**	0.37	0.25	**0.85**	**0.93**	Partition by sex, LAVE(+)
		(+)	MF	313	12.2	14.6	13.9	30.2	60.1	55.9	63.0				0.43	**1.10**	
		(-)	M	195	13.7	18.1	17.1	33.1	63.9	59.1	67.8		-0.04	0.03			
		(+)	M	157	12.9	18.1	**16.6**	**33.1**	**64.3**	58.7	67.6						
		(-)	F	224	5.7	8.4	6.7	26.3	52.6	50.2	56.6		**0.41**	-0.11			
		(+)	F	155	10.2	13.3	**11.5**	**27.5**	**51.3**	48.4	56.7						
**Ferr**	μg/L	(-)	MF	486	5.3	5.5	5.3	48	245	227	297	**1.252**	0.02	0.12	0.41	**2.06**	Partition by sex, LAVE(+) for males
		(+)	MF	350	6.1	7.4	6.6	57	252	240	323				0.38	**2.08**	
		(-)	M	221	19.7	29.9	30.1	92	265	248	343		-0.01	0.10			
		(+)	M	173	20.9	32.6	**29.8**	**97**	**271**	251	381						
		(-)	F	260	5.0	5.6	**5.3**	**21**	**139**	127	186		0.01	0.05			
		(+)	F	178	5.3	6.6	5.8	25	141	118	190						
**VitB12**	pmol/L	(-)	MF	482	107	123	119	278	644	602	686	0.175	0.02	0.11	-0.09	**-1.20**	Partition by sex, LAVE(-)
		(+)	MF	350	109	128	122	281	659	602	710				-0.06	**-1.32**	
		(-)	M	223	99	127	**113**	**263**	**549**	519	653		0.02	-0.16			
		(+)	M	174	98	129	115	268	532	504	617						
		(-)	F	259	110	132	**125**	**293**	**710**	647	761		-0.01	0.01			
		(+)	F	179	115	136	123	291	712	636	750						
**Folate**	μg/L	(-)	MF	486	3.04	3.42	3.33	6.87	19.2	17.7	20.6	0.276	0.00	-0.26	-0.11	**-1.12**	Partition by sex, LAVE(-)
		(+)	MF	350	3.03	3.34	3.34	6.76	18.2	15.9	20.2				-0.10	**-1.11**	
		(-)	M	225	2.78	3.31	**3.15**	**6.22**	**16.5**	14.1	18.0		0.00	0.01			
		(+)	M	176	2.84	3.33	3.16	6.22	16.6	13.5	18.9						
		(-)	F	261	3.29	3.69	**3.58**	**7.51**	**21.1**	18.3	22.7		-0.01	-0.07			
		(+)	F	178	3.01	3.80	3.53	7.25	20.8	17.1	24.4						

For LAVE method, 5 reference tests (Hb, MCH, MCHC, RDS-CV, Ret) were used allowing no abn value among them, but the RI limits were extended by 3% of the RI width so as not to exclude too many RVs

Suppl Table 4 A full list of RIs for leukocyte and platelet partitioned by sex

												BR<0.57		
												Gener-diff		
Item	Unit	Sex	N	90%CI of LL		LL	Me	UL	90%CI of UL		SDR_{sex}	BR_{LL}	BR_{UU}	decision
WBC	10^3 /µL	MF	457	3.37	3.63	**3.54**	**5.71**	**9.21**	8.86	9.72	0.167	0.20	0.19	No partition by sex
		M	199	3.64	3.96	3.74	5.91	9.33	8.89	10.25				
		F	258	3.14	3.56	3.45	5.55	9.06	8.75	9.69				
Neu	10^3 /µL	MF	480	1.53	1.70	**1.67**	**3.06**	**5.85**	5.73	6.42	0.000	0.24	0.27	No partition by sex
		M	223	1.66	1.88	1.78	3.04	5.97	5.45	6.97				
		F	258	1.42	1.61	1.53	3.07	5.69	5.54	6.55				
Eos	10^3 /µL	MF	459	0.037	0.045	0.043	0.135	0.487	0.435	0.533	0.219	0.08	**1.88**	Partition by sex
		M	200	0.042	0.054	**0.050**	**0.151**	**0.588**	0.524	0.689				
		F	257	0.033	0.043	**0.041**	**0.126**	**0.375**	0.342	0.430				
Bas	10^3 /µL	MF	460	0.010	0.012	**0.011**	**0.039**	**0.092**	0.090	0.100	0.000	-0.05	0.16	No partition by sex
		M	200	0.009	0.012	0.010	0.038	0.095	0.089	0.108				
		F	262	0.010	0.012	0.011	0.039	0.092	0.087	0.095				
Mon	10^3 /µL	MF	460	0.26	0.28	0.28	0.46	0.81	0.80	0.88	**0.414**	0.32	**1.08**	Partition by sex
		M	200	0.30	0.32	**0.31**	**0.51**	**0.88**	0.84	0.95				
		F	260	0.25	0.27	**0.27**	**0.43**	**0.73**	0.70	0.79				
Lym	10^3 /µL	MF	460	1.10	1.17	1.15	1.88	3.32	3.21	3.46	0.262	0.18	**0.68**	Partition by sex
		M	200	1.15	1.28	**1.23**	**2.01**	**3.55**	3.26	3.79				
		F	260	1.04	1.16	**1.13**	**1.78**	**3.17**	2.91	3.33				
Neu%	%	MF	485	36.5	39.9	**38.8**	**54.1**	**70.1**	68.9	71.8	0.250	0.18	-0.25	No partition by sex
		M	225	38.2	40.0	39.0	52.1	68.6	67.1	71.3				
		F	262	35.7	39.8	37.6	55.9	70.6	69.3	71.8				
Eos%	%	MF	485	0.68	0.83	0.77	2.38	7.73	7.06	8.30	0.172	0.03	**1.18**	Partition by sex
		M	226	0.70	0.91	**0.80**	**2.59**	**8.66**	7.84	9.39				
		F	259	0.63	0.82	**0.75**	**2.24**	**6.56**	5.80	7.37				
Bas%	%	MF	483	0.19	0.22	**0.21**	**0.67**	**1.51**	1.44	1.65	0.134	-0.09	-0.18	No partition by sex
		M	223	0.16	0.21	0.19	0.62	1.48	1.34	1.67				
		F	261	0.20	0.24	0.22	0.70	1.54	1.46	1.72				
Mon%	%	MF	482	4.98	5.37	5.28	8.20	11.91	11.68	12.47	**0.367**	**0.60**	**0.67**	Partition by sex
		M	222	5.61	6.16	**5.93**	**8.64**	**12.42**	12.07	12.89				
		F	260	4.64	5.11	**4.91**	**7.82**	**11.29**	10.90	11.92				
Lym%	%	MF	484	19.2	20.8	**20.6**	**33.8**	**47.9**	47.2	50.3	0.099	-0.04	-0.08	No partition by sex
		M	226	18.6	21.8	20.3	34.9	47.8	46.7	49.0				
		F	260	19.0	21.9	20.6	32.8	48.4	47.2	50.8				
PLT	10^3 /µL	MF	485	117	133	131	216	317	307	325	**0.317**	-0.06	**-0.90**	Partition by sex
		M	222	108	135	**132**	**205**	**288**	279	302				
		F	261	120	142	**135**	**227**	**330**	318	339				
MPV	fL	MF	487	7.35	7.50	**7.41**	**9.14**	**11.80**	11.64	12.03	0.129	-0.07	0.44	No partition by sex
		M	225	7.24	7.54	7.37	9.32	12.05	11.70	12.33				
		F	262	7.33	7.60	7.45	8.98	11.55	11.23	11.85				
PDW	%	MF	406	15.8	16.0	**16.0**	**16.8**	**17.9**	18.0	18.5	0.171	0.39	**0.82**	No partition by sex
		M	168	15.9	16.2	16.1	16.9	18.1	18.1	19.1				
		F	236	15.7	15.9	15.9	16.8	17.7	17.7	18.1				

For LAVE method, 5 reference tests (Hb, MCH, MCHC, RDS-CV, Ret) were used allowing no abn value among them, but the RI limits were extended by 3% of the RI width so as not to exclude too many RVs

Suppl Table 5 A list of RIs partitioned by age at 45 years

						Parametric method								BR<0.375		BR<0.57		
													SDR_{age}	BR by LAVE		BR by age		Decision
Analyte	Unit	LAVE	Sex	Age	n	90%CI of LL		LL	Me	UL	90%CI of UL			LL	UL	LL	UL	
Ferr	μg/L	(-)	F	All	260	5.0	5.6	5.3	21	139	135	186	0.656	0.00	0.04	0.00	**-4.59**	Partition by age for female LAVE(-)
		(+)		All	238	5.0	5.6	5.4	22	138	122	182				-0.01	**-4.68**	
		(-)		~45	150	4.7	5.8	**5.5**	**15**	**67**	56	94		0.00	-0.02			
		(+)		~45	143	5.0	5.6	5.5	15	67	58	93						
		(-)		45~	111	4.7	5.9	**5.3**	**37**	**223**	174	261		-0.01	-0.03			
		(+)		45~	100	4.9	6.7	5.8	40	225	170	287						
RDW-SD	fL	(-)	M	All	224	36.7	37.4	37.3	40.8	47.2	46.4	47.9	0.470	-0.10	**0.65**	-0.40	-0.42	No partition by age for male due to small data. Thus, no need to partion by sex
		(+)		All	177	37.3	37.8	**37.5**	**40.7**	**45.5**	44.8	46.4				-0.42	**-1.06**	
		(-)		~45	140	36.5	37.4	37.1	40.2	46.6	45.0	47.7		-0.05	**0.51**			
		(+)		~45	98	37.0	37.5	**37.2**	**40.1**	**45.4**	44.8	46.4						
		(-)		45~	84	37.8	38.6	**38.1**	**41.8**	**47.7**	46.3	48.8		0.01	0.05			
		(+)		45~	78	37.7	38.6	38.1	41.6	47.6	45.6	49.6						

Suppl Table 6 A list of RIs with/without limiting BMI below 28 kg/m^2

												BR <0.375		BR <0.375		
												BR for LAVE effec		BR for limiting BMI		Decision
Analyte	BMI	LAVE	Sex	N	90%CI of LL		LL	Me	UL	90%CI of UL		LL	UL	LL	UL	
RBC	All	(-)	M	223	4.21	4.55	4.38	4.95	5.63	5.49	5.77	**-0.42**	0.03	**0.45**	**0.53**	BMI limit≦28 for male LAVE(+)
RBC	All	(+)	M	179	4.46	4.56	4.51	4.96	5.62	5.51	5.72			0.16	**0.56**	
RBC	<28	(-)	M	157	4.11	4.36	4.23	4.92	5.46	5.40	5.53	**-0.74**	0.00			
RBC	<28	(+)	M	124	4.41	4.53	**4.46**	**4.92**	**5.46**	5.39	5.51					
RBC	All	(-)	F	262	3.78	3.91	3.85	4.40	5.16	5.06	5.25	-0.21	0.12	0.11	**0.41**	BMI limit≦28 for female LAVE(+)**
RBC	All	(+)	F	188	3.88	3.96	3.92	4.39	5.12	5.02	5.22			0.11	**0.57**	
RBC	<28	(-)	F	181	3.74	3.90	3.81	4.33	5.02	4.93	5.08	-0.23	0.25			
RBC	<28	(+)	F	132	3.84	3.94	**3.88**	**4.34**	**4.94**	4.83	5.05					
Hb	All	(-)	M	223	132	137	**134**	**151**	**166**	165	168	-0.22	-0.06	**0.74**	0.28	No BMI limit for male LAVE(-)**
Hb	All	(+)	M	183	134	138	136	151	167	165	168			0.03	0.23	
Hb	<28	(-)	M	157	125	135	128	150	164	163	166	**-0.83**	-0.11			
Hb	<28	(+)	M	128	134	138	136	150	165	164	167					
Hb	All	(-)	F	261	102	109	105	131	150	149	152	**-0.93**	-0.13	0.05	0.26	BMI limit≦28 for female LAVE(+)
Hb	All	(+)	F	195	113	119	116	133	152	150	154			0.22	**0.45**	
Hb	<28	(-)	F	181	100	109	105	129	147	145	149	**-0.85**	-0.04			
Hb	<28	(+)	F	139	112	116	**114**	**130**	**148**	145	151					
Ht	All	(-)	M	225	38.1	39.9	39.0	44.0	48.4	47.8	49.0	**-0.45**	0.05	0.08	0.16	No BMI limit for male LAVE(+)
Ht	All	(+)	M	179	39.7	40.5	**40.1**	**44.0**	**48.3**	47.8	48.8			0.10	0.27	
Ht	<28	(-)	M	157	38.0	39.6	38.8	43.6	48.0	47.4	48.5	**-0.45**	0.13			
Ht	<28	(+)	M	124	39.2	40.3	39.9	43.7	47.7	47.0	48.3					
Ht	All	(-)	F	261	30.9	33.2	32.1	38.7	44.2	43.7	44.7	**-0.90**	-0.07	0.06	0.26	BMI limit≦28 for female LAVE(+)
Ht	All	(+)	F	188	34.5	35.1	34.8	39.0	44.4	43.7	45.1			0.31	**0.77**	
Ht	<28	(-)	F	181	30.8	32.5	31.9	38.0	43.4	42.3	44.0	**-0.76**	0.29			
Ht	<28	(+)	F	132	33.6	34.7	**34.1**	**38.4**	**42.5**	42.0	42.9					
Ret	All	(-)	MF	396	23.1	26.6	24.8	50.2	96.0	90.3	101.8	-0.07	0.01	0.10	**0.66**	BMI limit≦28 for M+F LAVE(-)
Ret	All	(+)	MF	304	24.6	27.7	26.2	50.9	95.9	90.8	100.9			0.15	**0.58**	
Ret	<28	(-)	MF	270	21.3	24.8	**23.0**	**45.6**	**84.1**	79.8	87.1	-0.04	-0.09			
Ret	<28	(+)	MF	222	22.7	25.6	23.6	45.7	85.6	82.2	90.6					
Ret%	All	(-)	MF	396	0.56	0.60	0.58	1.1	2.05	1.97	2.12	-0.12	0.21	0.11	**0.75**	BMI limit≦28 for M+F LAVE(-)
Ret%	All	(+)	MF	296	0.60	0.66	0.63	1.1	1.97	1.88	2.06			0.14	**0.61**	
Ret%	<28	(-)	MF	269	0.52	0.57	**0.54**	**1.0**	**1.77**	1.70	1.85	-0.12	0.02			
Ret%	<28	(+)	MF	215	0.56	0.61	0.58	1.0	1.76	1.66	1.84					
IRF	All	(-)	MF	396	23.4	25.5	**24.5**	**36.8**	**49.8**	48.7	50.9	0.12	0.22	0.21	0.22	No BMI limit for M+F LAVE(-)
IRF	All	(+)	MF	296	22.3	25.1	23.7	36.3	48.4	47.4	49.3			0.18	0.16	
IRF	<28	(-)	MF	269	22.0	24.0	23.1	35.6	48.4	47.3	49.3	0.09	0.16			
IRF	<28	(+)	MF	215	21.1	23.7	22.5	35.4	47.4	45.9	48.7					

** decided by consensus to avoid interaction of LAVE effect and BMI effect

Figure 1 Click here to access/download;Figure;Fig 1.pdf

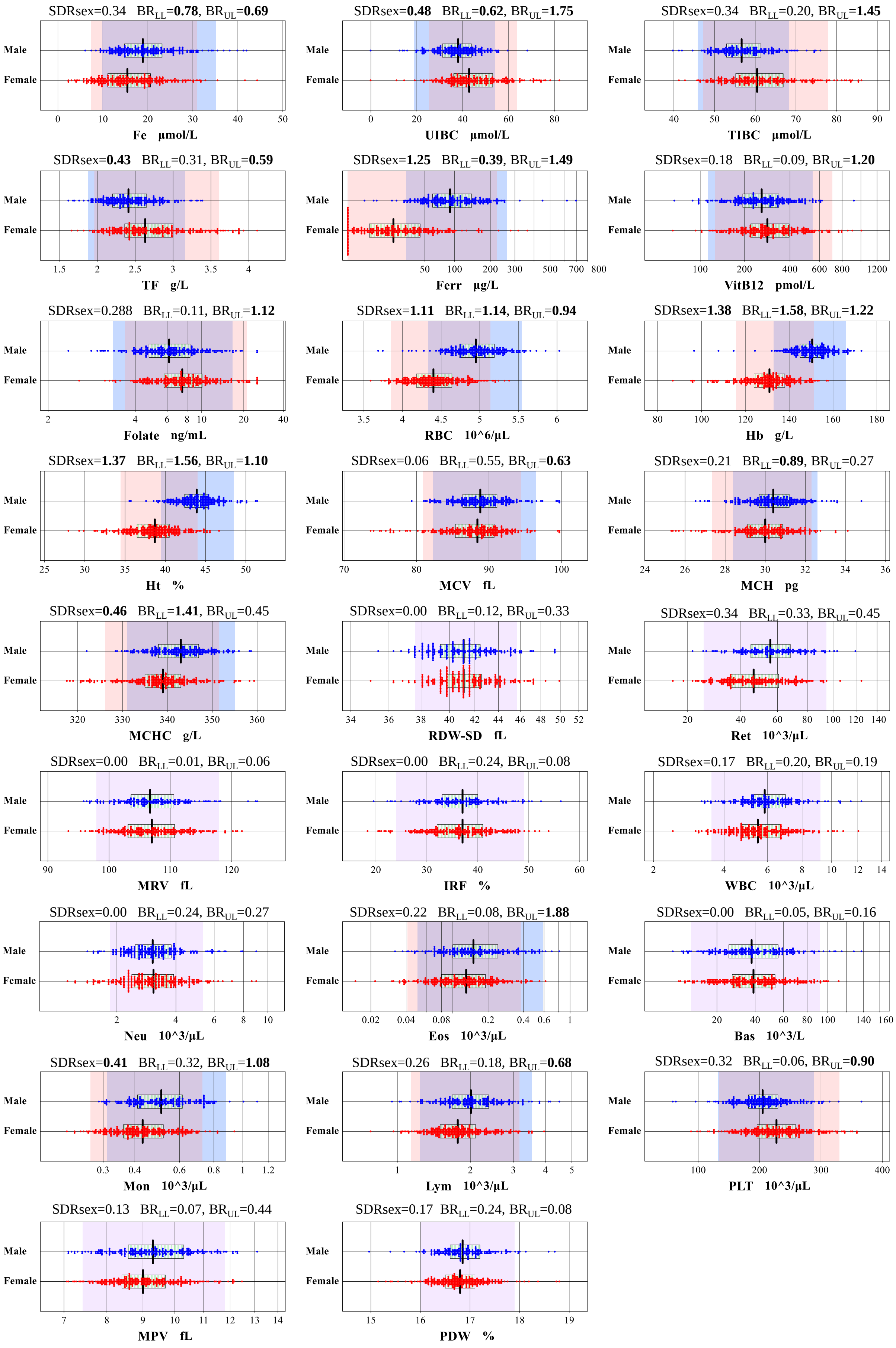

Figure 2

Click here to access/download;Figure;Fig 2.pdf

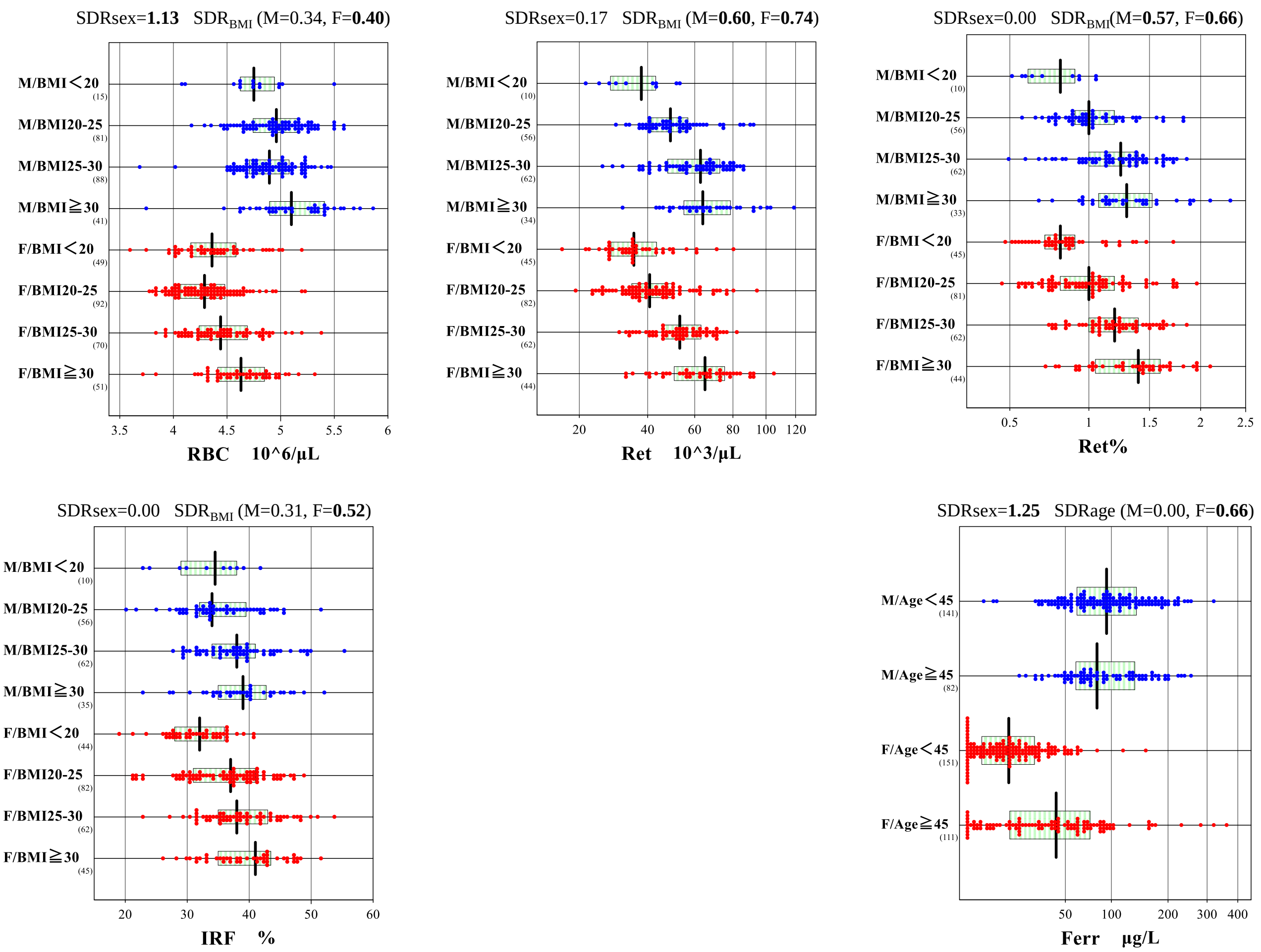

Figure 3

Click here to access/download;Figure;Fig 3.pdf

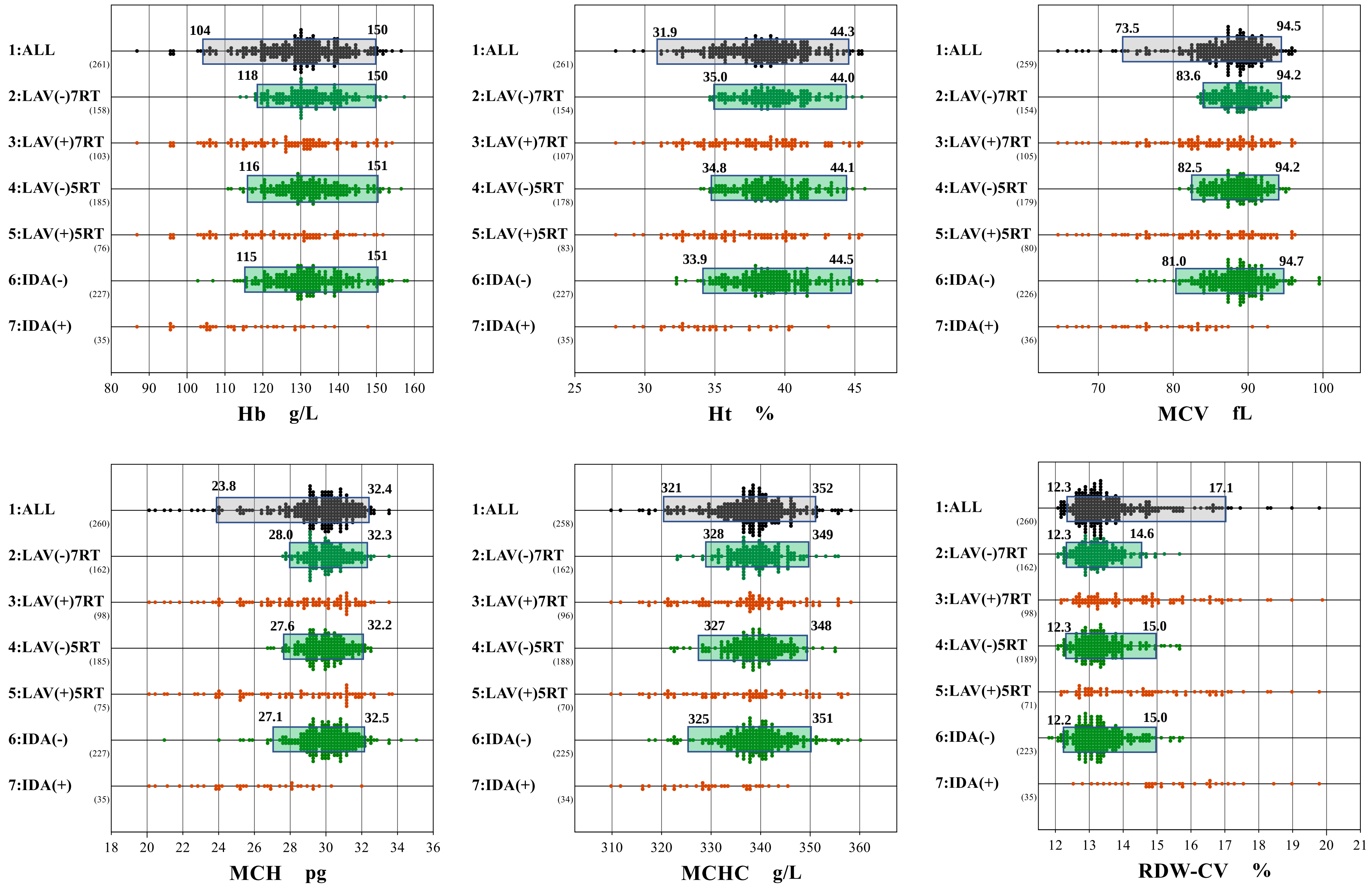

Figure 4A

Click here to access/download;Figure;Fig 4 A.pdf

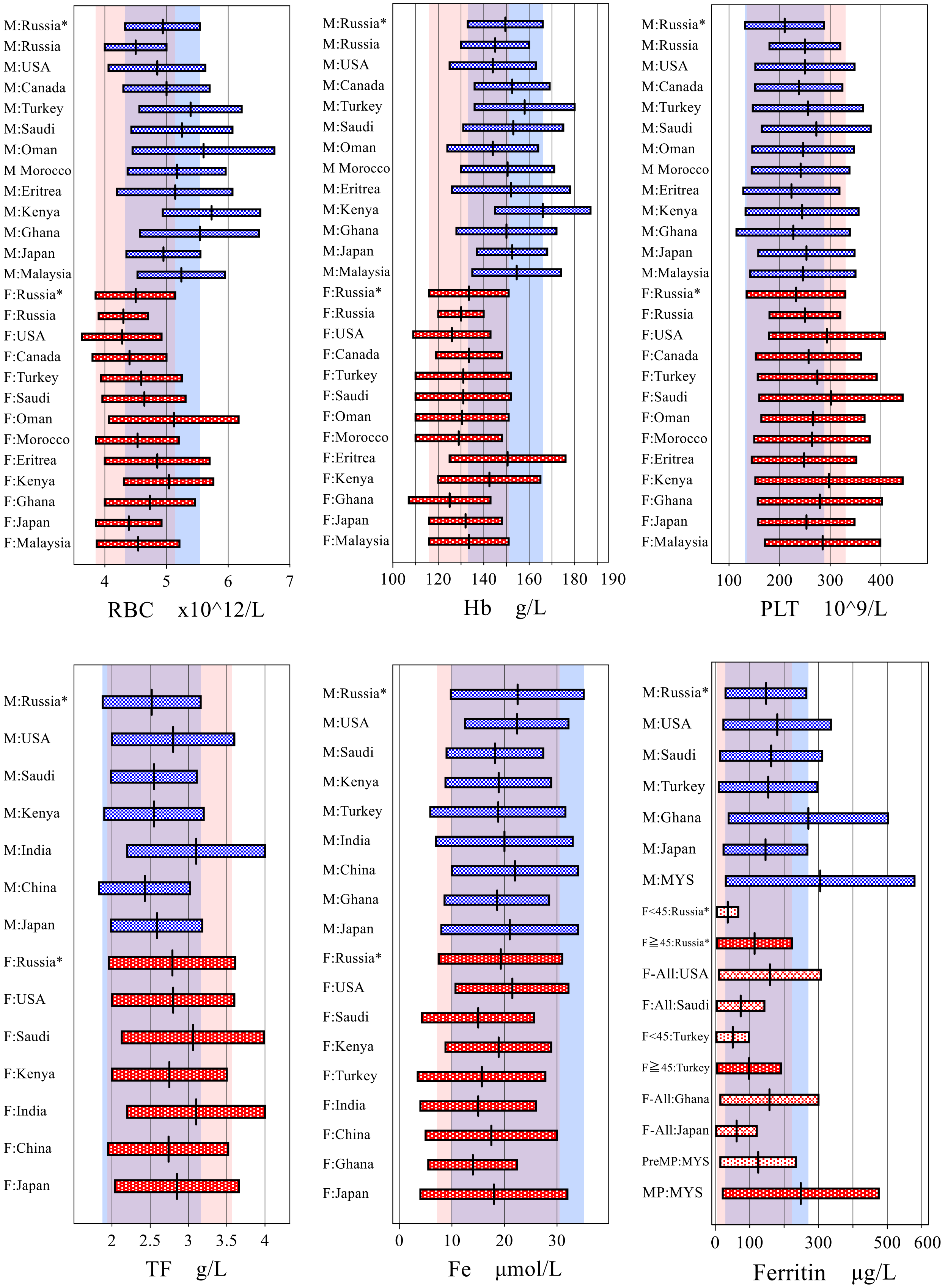

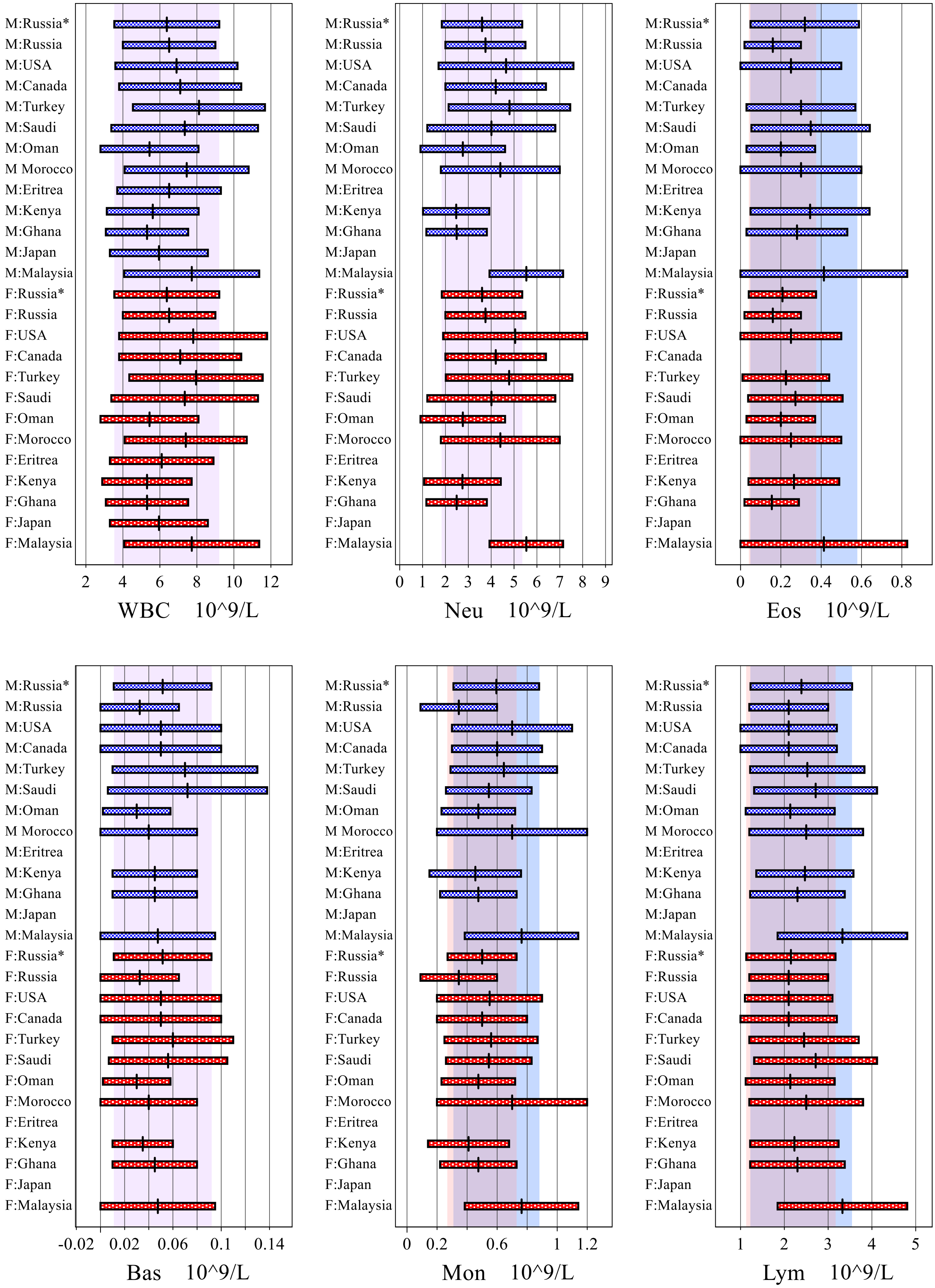

M:Russia*
M:Russia
M:USA
M:Canada
M:Turkey
M:Saudi
M:Oman
M Morocco
M:Eritrea
M:Kenya
M:Ghana
M:Japan
M:Malaysia
F:Russia*
F:Russia
F:USA
F:Canada
F:Turkey
F:Saudi
F:Oman
F:Morocco
F:Eritrea
F:Kenya
F:Ghana
F:Japan
F:Malaysia
2 4 6 8 10 12
WBC 10^9/L
0 1 2 3 4 5 6 7 8 9
Neu 10^9/L
0 0.2 0.4 0.6 0.8
Eos 10^9/L
-0.02 0.02 0.06 0.1 0.14
Bas 10^9/L
0 0.2 0.6 1 1.2
Mon 10^9/L
1 2 3 4 5
Lym 10^9/L

Figure 4 B

Click here to access/download;Figure;Fig 4 B.pdf

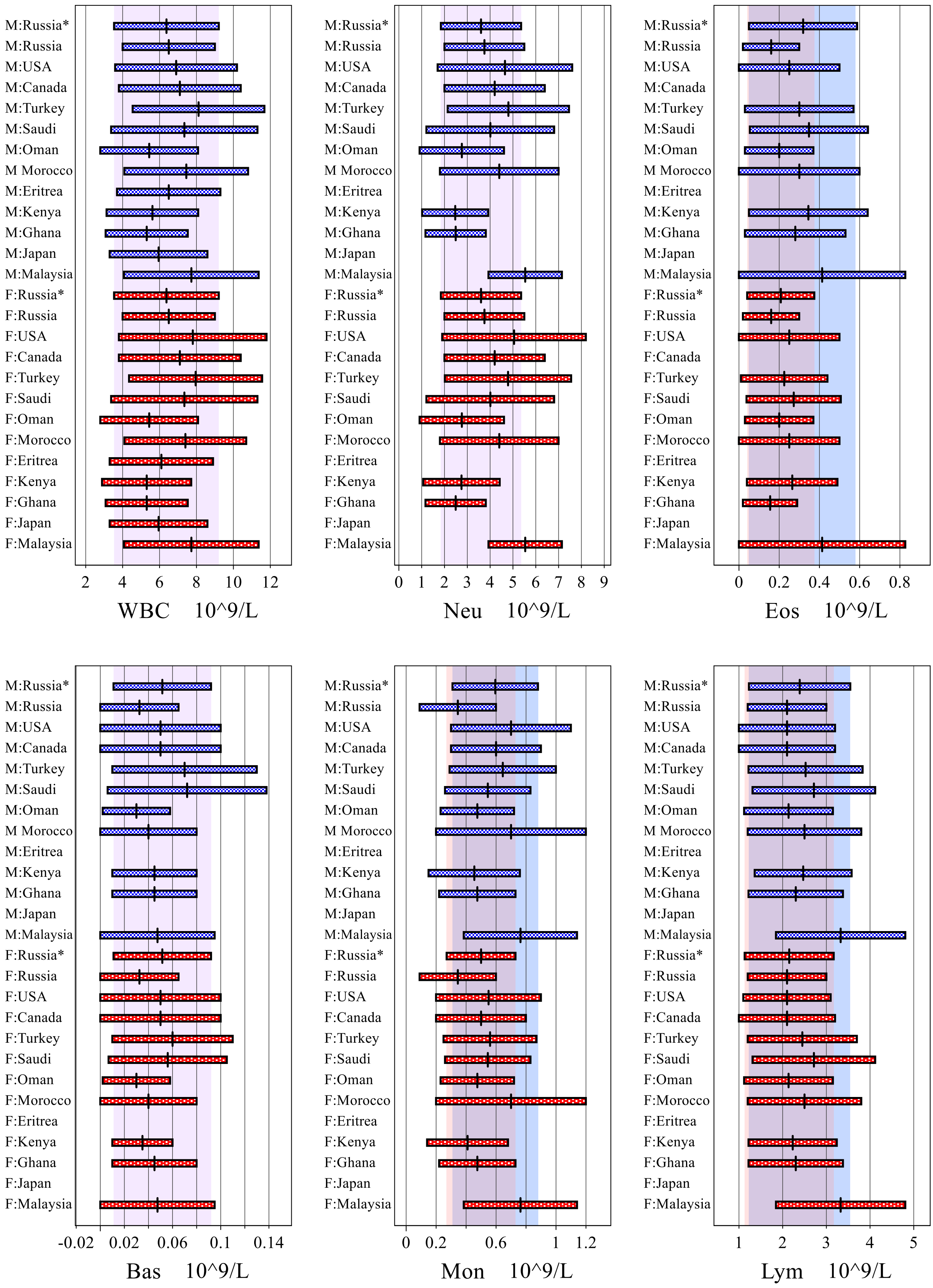

Supp Figure 1

Click here to access/download;Figure;Suppl Fig 1.pdf

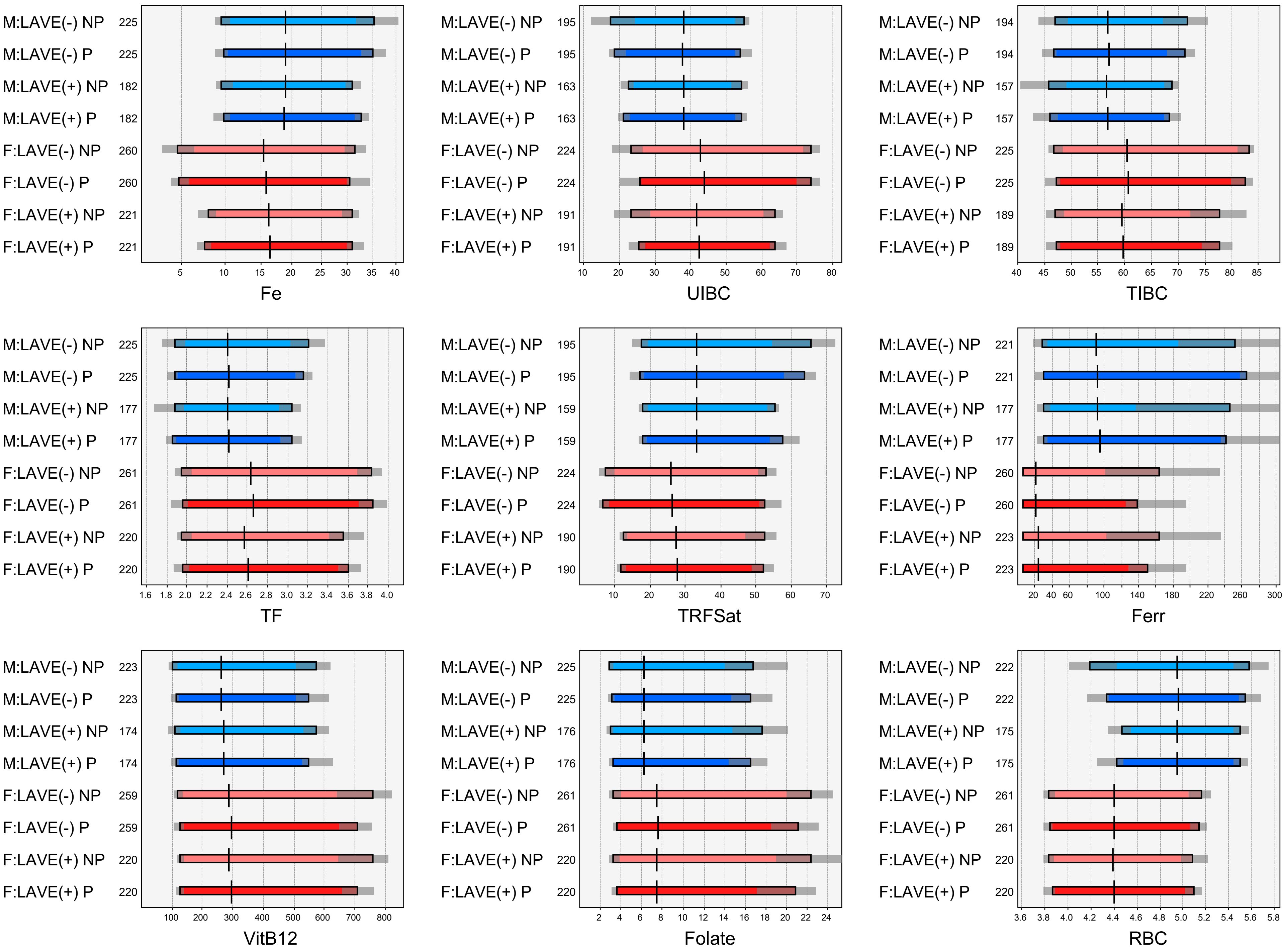

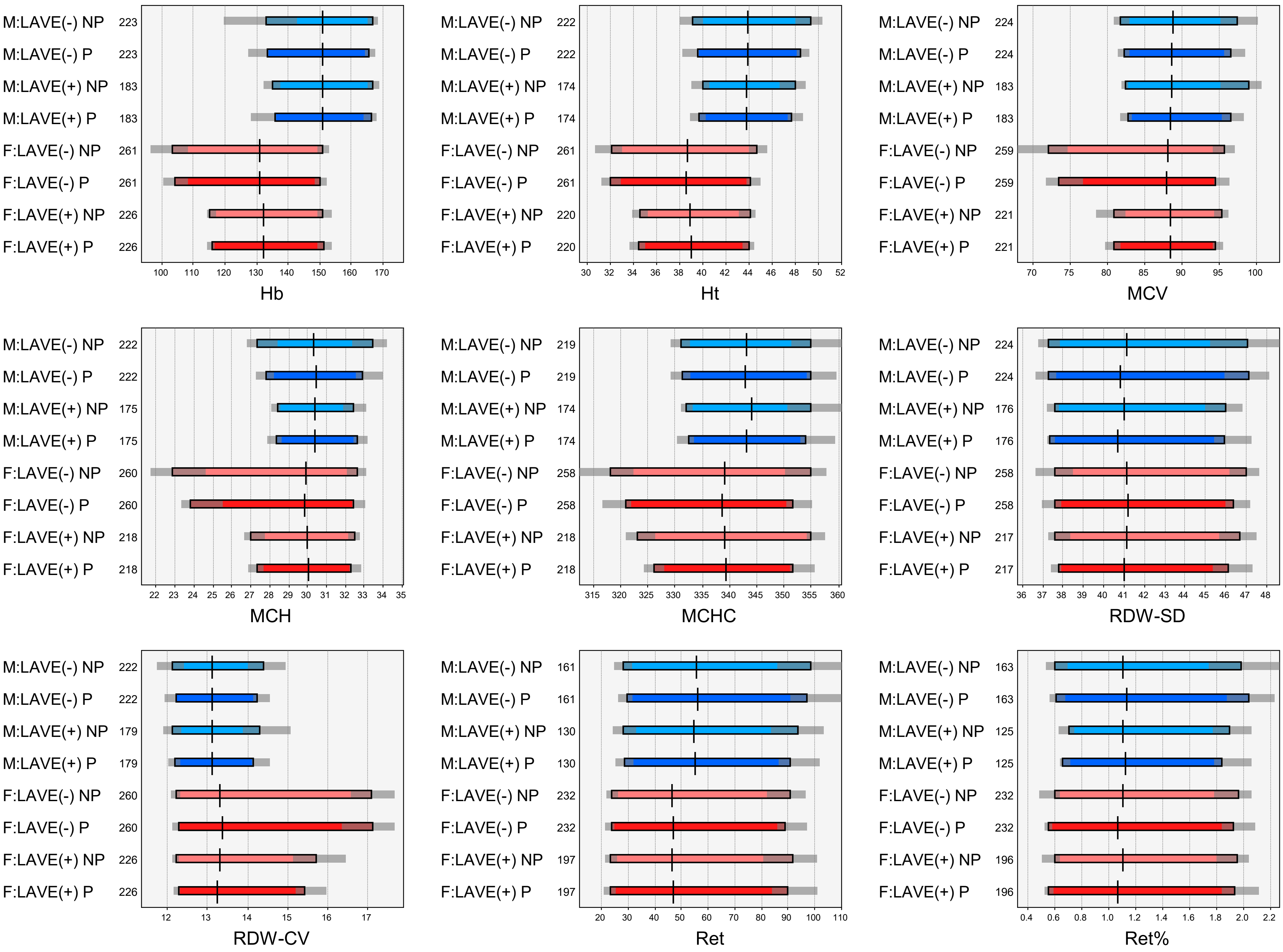
M:LAVE(-) NP 223
M:LAVE(-) P 223
M:LAVE(+) NP 183
M:LAVE(+) P 183
F:LAVE(-) NP 261
F:LAVE(-) P 261
F:LAVE(+) NP 226
F:LAVE(+) P 226
100 110 120 130 140 150 160 170
Hb
M:LAVE(-) NP 222
M:LAVE(-) P 222
M:LAVE(+) NP 174
M:LAVE(+) P 174
F:LAVE(-) NP 261
F:LAVE(-) P 261
F:LAVE(+) NP 220
F:LAVE(+) P 220
30 32 34 36 38 40 42 44 46 48 50 52
Ht
M:LAVE(-) NP 224
M:LAVE(-) P 224
M:LAVE(+) NP 183
M:LAVE(+) P 183
F:LAVE(-) NP 259
F:LAVE(-) P 259
F:LAVE(+) NP 221
F:LAVE(+) P 221
70 75 80 85 90 95 100
MCV
M:LAVE(-) NP 222
M:LAVE(-) P 222
M:LAVE(+) NP 175
M:LAVE(+) P 175
F:LAVE(-) NP 260
F:LAVE(-) P 260
F:LAVE(+) NP 218
F:LAVE(+) P 218
22 23 24 25 26 27 28 29 30 31 32 33 34 35
MCH
M:LAVE(-) NP 219
M:LAVE(-) P 219
M:LAVE(+) NP 174
M:LAVE(+) P 174
F:LAVE(-) NP 258
F:LAVE(-) P 258
F:LAVE(+) NP 218
F:LAVE(+) P 218
315 320 325 330 335 340 345 350 355 360
MCHC
M:LAVE(-) NP 224
M:LAVE(-) P 224
M:LAVE(+) NP 176
M:LAVE(+) P 176
F:LAVE(-) NP 258
F:LAVE(-) P 258
F:LAVE(+) NP 217
F:LAVE(+) P 217
36 37 38 39 40 41 42 43 44 45 46 47 48
RDW-SD
M:LAVE(-) NP 222
M:LAVE(-) P 222
M:LAVE(+) NP 179
M:LAVE(+) P 179
F:LAVE(-) NP 260
F:LAVE(-) P 260
F:LAVE(+) NP 226
F:LAVE(+) P 226
12 13 14 15 16 17
RDW-CV
M:LAVE(-) NP 161
M:LAVE(-) P 161
M:LAVE(+) NP 130
M:LAVE(+) P 130
F:LAVE(-) NP 232
F:LAVE(-) P 232
F:LAVE(+) NP 197
F:LAVE(+) P 197
20 30 40 50 60 70 80 90 100 110
Ret
M:LAVE(-) NP 163
M:LAVE(-) P 163
M:LAVE(+) NP 125
M:LAVE(+) P 125
F:LAVE(-) NP 232
F:LAVE(-) P 232
F:LAVE(+) NP 196
F:LAVE(+) P 196
0.4 0.6 0.8 1.0 1.2 1.4 1.6 1.8 2.0 2.2
Ret%

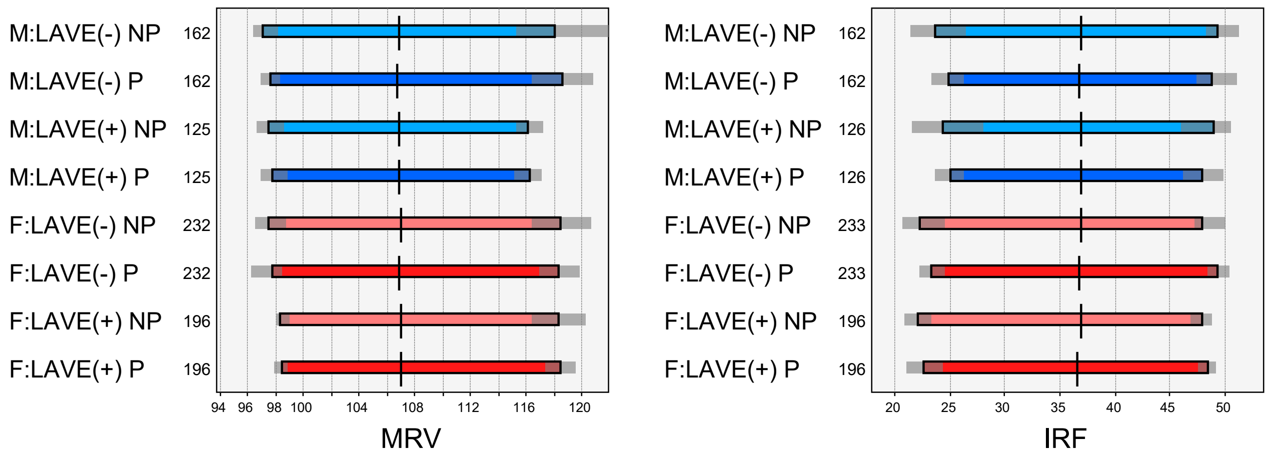

M:LAVE(-) NP
162
M:LAVE(-) P
162
M:LAVE(+) NP
125
M:LAVE(+) P
125
F:LAVE(-) NP
232
F:LAVE(-) P
232
F:LAVE(+) NP
196
F:LAVE(+) P
196
94 96 98 100 104 108 112 116 120
MRV
M:LAVE(-) NP
162
M:LAVE(-) P
162
M:LAVE(+) NP
126
M:LAVE(+) P
126
F:LAVE(-) NP
233
F:LAVE(-) P
233
F:LAVE(+) NP
196
F:LAVE(+) P
196
20 25 30 35 40 45 50
IRF
