# Supplementary material for: Derivation of Russian-specific reference intervals for complete blood count, iron markers and related vitamins
Source: PLoS One. 2024 Sep 30;19(9):e0304020. doi: 10.1371/journal.pone.0304020 (PMC11441669; doi:10.1371/journal.pone.0304020)
Supplement: Attachment — Submitted filename: Responses to Reviewers for R1.docx [file pone.0304020.s005.docx]

Journal requirements:

When submitting your revision, we need you to address these additional requirements.

1. Please ensure that your manuscript meets PLOS ONE's style requirements, including those for file naming. The PLOS ONE style templates can be found at

[https://journals.plos.org/plosone/s/file?id=wjVg/PLOSOne_formatting_sample_main_body.pdf](https://urldefense.proofpoint.com/v2/url?u=https-3A__journals.plos.org_plosone_s_file-3Fid-3DwjVg_PLOSOne-5Fformatting-5Fsample-5Fmain-5Fbody.pdf&d=DwMDaQ&c=9mghv0deYPYDGP-W745IEdQLV1kHpn4XJRvR6xMRXtA&r=7LlEuRFcxcRwo-3j0-NnnGhMlKecQE_GYm1wzH5gfgw&m=dBd-qjSVnQA7Oso202BjUW_6Z8DH2nJz0Sh_6Yxc2hOis6hbrVrFEUmQkcvPzF8S&s=3Av5QBoQYHAqcZtrFCmeSQuyUZSYicDQZakAbpiacIE&e=) and

[https://journals.plos.org/plosone/s/file?id=ba62/PLOSOne_formatting_sample_title_authors_affiliations.pdf](https://urldefense.proofpoint.com/v2/url?u=https-3A__journals.plos.org_plosone_s_file-3Fid-3Dba62_PLOSOne-5Fformatting-5Fsample-5Ftitle-5Fauthors-5Faffiliations.pdf&d=DwMDaQ&c=9mghv0deYPYDGP-W745IEdQLV1kHpn4XJRvR6xMRXtA&r=7LlEuRFcxcRwo-3j0-NnnGhMlKecQE_GYm1wzH5gfgw&m=dBd-qjSVnQA7Oso202BjUW_6Z8DH2nJz0Sh_6Yxc2hOis6hbrVrFEUmQkcvPzF8S&s=T09Fsl47RlnsG2JN8QxkExr3RdAYIUyUldiudKwBOV4&e=)

2. We note that you have indicated that data from this study are available upon request. PLOS only allows data to be available upon request if there are legal or ethical restrictions on sharing data publicly. For more information on unacceptable data access restrictions, please see [http://journals.plos.org/plosone/s/data-availability#loc-unacceptable-data-access-restrictions](https://urldefense.proofpoint.com/v2/url?u=http-3A__journals.plos.org_plosone_s_data-2Davailability-23loc-2Dunacceptable-2Ddata-2Daccess-2Drestrictions&d=DwMDaQ&c=9mghv0deYPYDGP-W745IEdQLV1kHpn4XJRvR6xMRXtA&r=7LlEuRFcxcRwo-3j0-NnnGhMlKecQE_GYm1wzH5gfgw&m=dBd-qjSVnQA7Oso202BjUW_6Z8DH2nJz0Sh_6Yxc2hOis6hbrVrFEUmQkcvPzF8S&s=_EoRJH8dnkihYV99sIlu2e8eCwBivgvDtJ0jiAY-4ts&e=).

In your revised cover letter, please address the following prompts:

a) If there are ethical or legal restrictions on sharing a de-identified data set, please explain them in detail (e.g., data contain potentially sensitive information, data are owned by a third-party organization, etc.) and who has imposed them (e.g., an ethics committee). Please also provide contact information for a data access committee, ethics committee, or other institutional body to which data requests may be sent.

b) If there are no restrictions, please upload the minimal anonymized data set necessary to replicate your study findings as either Supporting Information files or to a stable, public repository and provide us with the relevant URLs, DOIs, or accession numbers. For a list of acceptable repositories, please see [http://journals.plos.org/plosone/s/data-availability#loc-recommended-repositories](https://urldefense.proofpoint.com/v2/url?u=http-3A__journals.plos.org_plosone_s_data-2Davailability-23loc-2Drecommended-2Drepositories&d=DwMDaQ&c=9mghv0deYPYDGP-W745IEdQLV1kHpn4XJRvR6xMRXtA&r=7LlEuRFcxcRwo-3j0-NnnGhMlKecQE_GYm1wzH5gfgw&m=dBd-qjSVnQA7Oso202BjUW_6Z8DH2nJz0Sh_6Yxc2hOis6hbrVrFEUmQkcvPzF8S&s=vhojvvwfw61kq8GIeRWyWRnpULA4EWDFZLH-KFt1phs&e=).

We will update your Data Availability statement on your behalf to reflect the information you provide.

3. We notice that your supplementary tables are included in the manuscript file. Please remove them and upload them with the file type 'Supporting Information'. Please ensure that each Supporting Information file has a legend listed in the manuscript after the references list.

4. We notice that your supplementary figures are uploaded with the file type 'Figure'. Please amend the file type to 'Supporting Information'. Please ensure that each Supporting Information file has a legend listed in the manuscript after the references list.

Additional Editor Comments:

The reviewers has returned their reviews with comments. Please go through and address issues raised adequately


[Note: HTML markup is below. Please do not edit.]

Reviewers' comments:

Reviewer's Responses to Questions

**Comments to the Author**

1. Is the manuscript technically sound, and do the data support the conclusions?

The manuscript must describe a technically sound piece of scientific research with data that supports the conclusions. Experiments must have been conducted rigorously, with appropriate controls, replication, and sample sizes. The conclusions must be drawn appropriately based on the data presented.

Reviewer #1: Yes

Reviewer #2: No

Reviewer #3: Yes

Our responses→ The internationally harmonized protocol stipulated by IFCC, Committee on Reference Intervals and Decision Limits was closely followed, especially for recruitment and sample size. We’ve also taken into account factors specific to our study, hence the manuscript is technically sound and the data supports the conclusions.

2. Has the statistical analysis been performed appropriately and rigorously?

Reviewer #1: Yes

Reviewer #2: No

Reviewer #3: I Don't Know

Our responses→According to the suggestion by Reviewer #2, we added more detailed descriptions on the methodological issues used in this study, rather than simply citing previous papers.

3. Have the authors made all data underlying the findings in their manuscript fully available?

The [PLOS Data policy](https://urldefense.proofpoint.com/v2/url?u=http-3A__www.plosone.org_static_policies.action-23sharing&d=DwMDaQ&c=9mghv0deYPYDGP-W745IEdQLV1kHpn4XJRvR6xMRXtA&r=7LlEuRFcxcRwo-3j0-NnnGhMlKecQE_GYm1wzH5gfgw&m=dBd-qjSVnQA7Oso202BjUW_6Z8DH2nJz0Sh_6Yxc2hOis6hbrVrFEUmQkcvPzF8S&s=Q6XVefBe8R2hWG1EkuYnLbgfJQe25x4-ZM3j1w7zxTs&e=) requires authors to make all data underlying the findings described in their manuscript fully available without restriction, with rare exception (please refer to the Data Availability Statement in the manuscript PDF file). The data should be provided as part of the manuscript or its supporting information, or deposited to a public repository. For example, in addition to summary statistics, the data points behind means, medians and variance measures should be available. If there are restrictions on publicly sharing data—e.g. participant privacy or use of data from a third party—those must be specified.

Reviewer #1: Yes

Reviewer #2: No

Reviewer #3: Yes

Our responses→ We had explicitly declared to make the source data available on request from researchers. However, we chose to upload the source data as supplemental material in submitting the revised manuscript.

4. Is the manuscript presented in an intelligible fashion and written in standard English?

PLOS ONE does not copyedit accepted manuscripts, so the language in submitted articles must be clear, correct, and unambiguous. Any typographical or grammatical errors should be corrected at revision, so please note any specific errors here.

Reviewer #1: Yes

Reviewer #2: No

Reviewer #3: Yes

Our responses→Final manuscript has been copy edited in standard English.

5. Review Comments to the Author

Please use the space provided to explain your answers to the questions above. You may also include additional comments for the author, including concerns about dual publication, research ethics, or publication ethics. (Please upload your review as an attachment if it exceeds 20,000 characters)

--------------------------------------------------------------

**Reviewer #1**: Data on the RIs when iron deficiency is excluded would be useful. This is said to be shown in a figure but I cannot see the figure. A Table similar to Table 3 would be informative. The differences from other published ranges are interesting and informative.

Our responses→Thanks for your time and efforts for reviewing our manuscript.

Data on the RIs when iron deficiency was excluded were listed in Table 3. As for the figure, it was clearly indicated in Figure 3. In the figure, we realized that amongst the three methods of excluding iron deficiency, it was difficult to understand which one we finally adopted. Therefore, we updated the figure by emphasizing the second method (shown in the 4^th^ row) was the one we adopted by using dark-green shade and RI limits in red

Female RIs of 6 representative hematology tests were determined parametrically using the following 4 sets of RV datasets.

1: All: RVs without any prior exclusion

2: LAVE(-) 7RT: RVs with no abnormal value among 7 tests (Hb, MCH, MCHC, RDW-CV, Ret, Fe, Ferr) other than itself

4: LAVE(-) 5RT: RVs with no abnormal value among 5 ref tests (Hb, MCH, MCHC, RDW-CV, Ret) other than itself

6: IDA(-): RVs with no abnormal results for Fe and Ferr from respective lower limit of the manufacturer’s RI.

Each bar shown over the scattergram represents the span of RI with its lower and upper limits indicated by numerical labels. Please note that RIs indicated by green shade (at the 4^th^ row) correspond to what we adopted in this study. The three scattergrams of RVs shown in red dots in the 3rd, 5th, and 7th rows represent values that were excluded by the criteria applied to obtain RVs shown above in the 2nd, 4th, and 6th rows in green. Thus, their group labels were designated as follows:

As a reference, the following three datasets were prepared and plotted in the 3rd, 5th , and 7th row.

3: LAVE(+) 7RT: RVs with one or more abnormal values among 7 ref tests (Hb, MCH, MCHC, RDW-CV, Ret, Fe, Ferr)

5: LAVE(+) 5RT: RVs with one or more abnormal values among 5 ref tests (Hb, MCH, MCHC, RDW-CV, Ret)

7: IDA(+): RVs had abnormal results either for Fe or Ferr.

As for the last sentence of the comments, we appreciate your kind message regarding our international comparison of RI limits recent reported, which we laid out in Table 4 and Fig. 4.

**Reviewer #2**: The methodology for subject recruitment was not described adequately. The methodology needs to be written understandably. Avoid quoting other reference manuals as part of your method, but make efforts to describe your methodology thoroughly. I also noticed that your normal subjects included smokers and alcohol consumers. Did you compare the smokers versus non-smokers in your reference range calculation? Did you assess if the alcohol consumption affected the reference range calculation? On what basis have you decided to include smoking and alcohol consumption?

Our responses → We really appreciate your time and efforts for reviewing our manuscript and for providing us critical comments to improve our manuscript.

We apologize for the lack of sufficient descriptions on our methodology, especially for subject recruitment. We have added the descriptions extensively as red-marked in the revised manuscript.

Regarding the influence of smoking and drinking habits, we analyzed their associations with test results by calculating “partial correlation coefficient (r_p_)” using multiple regression analysis and listed them in **Table 1**, which was also included here on the right for your quick reference.

As we regarded, |r_p_|>0.30 as significant, smoking and drinking habits were deemed to not have influence on RVs, while BMI and age did influence RVs, for which we managed by restricting BMI at 28kg/m^2^, and by partitioning by age if necessary.

Regarding the inclusion of individuals with drinking and smoking habits, we followed the consensus reached among IFCC delegates regarding the protocol to “not exclude anyone who considered themselves as healthy” so long as the exclusion criteria were fulfilled. The lenient recruitment policy was adopted to facilitate recruitment, due to the difficulty in identifying “truly healthy individuals”. However, it certainly entailed the need for secondary exclusion of inappropriate individuals after the measurements, as we emphasized the issue in the revised Method section.

Also, the results are scattered without proper directions. It would be best to amend your methodology to reflect your result section accurately. There is no synchronization in the findings and method.

Our response → Thanks for the advice. In Methods, there were three subsections in “4) Statistical methods”.

4.1) Sources of variation of reference values

4.2) Criteria for partitioning RVs.

4.3) Derivation of reference intervals

These three sub sections have been matched to the three subheadings of the revised “Results” under the same name to ensure synchronization between the findings and methods, as suggested by the reviewer.

This paper also requires major English language editing. It is very difficult to understand the content in the current language.

Our responses → Thank you for pointing out the problem. The revised manuscript was thoroughly checked and edited by native English speaker with medical and scientific background.

A major revision is required; otherwise, i suggest rejecting this manuscript completely.

Our responses → We have made all changes as requested and addressed all comments.

**Reviewer #3**: The study is important as it define RIs of hematological parameters including related iron markers and vitamins for adult population in Russian in accordance with the harmonized protocol and explore their specific features from an international perspective. This will help avoid of diagnostic errors, allow clinicians to interpret with greater specificity the hematological examinations and to improve the quality of medical care distributed to patients.

Our responses → We really appreciate your time and efforts for reviewing our manuscript and providing us comments to improve our manuscript. We revised the manuscript as described below.

Here Some comments may need to clear
-What are the limitations of the study?
- Can the authors consider the selection of study participants based on their willingness to participate (with no specific randomization method)? ...is the main limitation of this study?

Our responses → We understand that this comment raises the issue of appropriateness of our sampling scheme in recruiting healthy volunteers. To address this issue, we set up a new subheading of “Limitations” at the end of Discussion and expressed our thoughts on the issue as shown below.

*“***Limitations**

The methodologies used in this study were in close alignment with internationally harmonized protocol elaborated by IFCC C-RIDL in 2012. Nevertheless, there are two limitations to the generalizability of our findings due to our recruitment scheme. One of the limitation was brought about by our adoption of convenient sampling targeting individuals who are easily accessible from clinical laboratories, like hospital personnels and their family, and anyone who could get access to the study-advertisement posted in the hospital. Therefore, unlike population-based random sampling recommended for epidemiological studies of public health interest, our sample population may not reflect individuals in quite different living environments and physical activities. Nevertheless, it is not practical to set up such a grand-scale population-based sampling just for deriving RIs. In fact, recent RI studies conducted in a nationwide scale in Japan [50], China [10], and Turkey[51], which basically adopted the C-RIDL scheme, reported no regional differences within each country in any of the biochemical analytes examined. Therefore, it is generally agreed upon that so long as life style, physical activity level and living environment are not biased, the convenient sampling is acceptable for determination of RIs.

Another limitation regarding the generalizability of our finding is the adoption of very lenient exclusion and inclusion criteria to attract sufficient number volunteers to participate. However, we made efforts to identify inappropriate individuals after measurements: i.e., restriction of individuals with obesity in determining RIs for analytes with BMI related changes, and exclusion of those exhibiting iron-deficiency state detected by the LAVE method.”

- Does the results indicated that the currently used reference interval does not represent the population in Russian.

Our responses →Thank you very much for pointing out this important question. For majority of the parameters, we didn’t find any significant differences of the RIs derived in the current study compared to the RIs in the manufacturer’s IFU, and RIs from Russian Methodological Recommendations (MR). However, prominent differences were observed in LL of RIs for Hb, Fe, Ferritin in females, which are meant to be used in diagnosing iron deficiency anemia (IDA). Their LLs of this study was much lower than that of MR, and also lower than the IDA cut-off value presented in Clinical Guidelines (AGA, WHO, local) and IFU (except Hb). LLs of our study for WBC, PLT, and UL for Neu in both sexes were lower than those of MR.

A potential limitation of the frequently used RIs from Russian Methodological Recommendations is that they were established a long time ago (more than 30 years for some parameters) and some of the analytical systems used in that analysis remain unknown.

In addition, further studies (e.g. clinical validation) would aid in subsequent validation of our findings, which remain the first set of RIs for the Russian population using up-to-date methods proposed by C-RIDL.

- The author should maintain a single format in all references (---year, volume, issue, page)—accordingly recheck references no. 41,44.

- Re-check the published date in year in References No.2.

- Recheck reference no. 4, 13 regarding the date of publication in day, month

- Recheck the published date in years in References No.20, and issue.

Our responses →Thank you for highlighting errors in the References. We have amended them accordingly, together with other typos and inconsistent formatting.

6. PLOS authors have the option to publish the peer review history of their article ([what does this mean?](https://urldefense.proofpoint.com/v2/url?u=https-3A__journals.plos.org_plosone_s_editorial-2Dand-2Dpeer-2Dreview-2Dprocess-23loc-2Dpeer-2Dreview-2Dhistory&d=DwMDaQ&c=9mghv0deYPYDGP-W745IEdQLV1kHpn4XJRvR6xMRXtA&r=7LlEuRFcxcRwo-3j0-NnnGhMlKecQE_GYm1wzH5gfgw&m=dBd-qjSVnQA7Oso202BjUW_6Z8DH2nJz0Sh_6Yxc2hOis6hbrVrFEUmQkcvPzF8S&s=hQMG9wxeXEO_U0Q_o_v_4eKznJQ0S-T8eWLGgufB1EQ&e=)). If published, this will include your full peer review and any attached files.


If you choose “no”, your identity will remain anonymous but your review may still be made public.


**Do you want your identity to be public for this peer review?** For information about this choice, including consent withdrawal, please see our [Privacy Policy](https://urldefense.proofpoint.com/v2/url?u=https-3A__www.plos.org_privacy-2Dpolicy&d=DwMDaQ&c=9mghv0deYPYDGP-W745IEdQLV1kHpn4XJRvR6xMRXtA&r=7LlEuRFcxcRwo-3j0-NnnGhMlKecQE_GYm1wzH5gfgw&m=dBd-qjSVnQA7Oso202BjUW_6Z8DH2nJz0Sh_6Yxc2hOis6hbrVrFEUmQkcvPzF8S&s=RZwmAoAHbjLkazv7rh5TUNy2LjyRfJmS2_QGnNEmNTw&e=).

Reviewer #1: No

Reviewer #2: No

Reviewer #3: No

[NOTE: If reviewer comments were submitted as an attachment file, they will be attached to this email and accessible via the submission site. Please log into your account, locate the manuscript record, and check for the action link "View Attachments". If this link does not appear, there are no attachment files.]

While revising your submission, please upload your figure files to the Preflight Analysis and Conversion Engine (PACE) digital diagnostic tool, [https://pacev2.apexcovantage.com/](https://urldefense.proofpoint.com/v2/url?u=https-3A__pacev2.apexcovantage.com_&d=DwMDaQ&c=9mghv0deYPYDGP-W745IEdQLV1kHpn4XJRvR6xMRXtA&r=7LlEuRFcxcRwo-3j0-NnnGhMlKecQE_GYm1wzH5gfgw&m=dBd-qjSVnQA7Oso202BjUW_6Z8DH2nJz0Sh_6Yxc2hOis6hbrVrFEUmQkcvPzF8S&s=zdxsAAS_J9Ysano0pCi9A1sjykbQ11A4rWYVBw6Tfp0&e=). PACE helps ensure that figures meet PLOS requirements. To use PACE, you must first register as a user. Registration is free. Then, login and navigate to the UPLOAD tab, where you will find detailed instructions on how to use the tool. If you encounter any issues or have any questions when using PACE, please email PLOS at [figures@plos.org](mailto:figures@plos.org). Please note that Supporting Information files do not need this step.

*In compliance with data protection regulations, you may request that we remove your personal registration details at any time.* [*(Remove my information/details)*](https://urldefense.proofpoint.com/v2/url?u=https-3A__www.editorialmanager.com_pone_login.asp-3Fa-3Dr&d=DwMDaQ&c=9mghv0deYPYDGP-W745IEdQLV1kHpn4XJRvR6xMRXtA&r=7LlEuRFcxcRwo-3j0-NnnGhMlKecQE_GYm1wzH5gfgw&m=dBd-qjSVnQA7Oso202BjUW_6Z8DH2nJz0Sh_6Yxc2hOis6hbrVrFEUmQkcvPzF8S&s=bjJx73olWR3OAjcf5k5_D9tU5E9d_HIWVACGB8f4Vs4&e=)*. Please contact the publication office if you have any questions.*

Please be advised that this email may contain confidential information. If you are not the intended recipient, please notify us by email by replying to the sender and delete this message. The sender disclaims that the content of this email constitutes an offer to enter into, or the acceptance of, any agreement; provided that the foregoing does not invalidate the binding effect of any digital or other electronic reproduction of a manual signature that is included in any attachment.
