# Supplementary material for: Derivation of Russian-specific reference intervals for complete blood count, iron markers and related vitamins
Source: PLoS One. 2024 Sep 30;19(9):e0304020. doi: 10.1371/journal.pone.0304020 (PMC11441669; doi:10.1371/journal.pone.0304020)
Supplement: Attachment — Submitted filename: Our responces to Reviewers for R2 V2.docx [file pone.0304020.s007.docx]

Reviewers' comments:

Reviewer's Responses to Questions

**Comments to the Author**

1. If the authors have adequately addressed your comments raised in a previous round of review and you feel that this manuscript is now acceptable for publication, you may indicate that here to bypass the “Comments to the Author” section, enter your conflict of interest statement in the “Confidential to Editor” section, and submit your "Accept" recommendation.

Reviewer #3: All comments have been addressed

Reviewer #4: (No Response)

Reviewer #5: (No Response)

2. Is the manuscript technically sound, and do the data support the conclusions?

The manuscript must describe a technically sound piece of scientific research with data that supports the conclusions. Experiments must have been conducted rigorously, with appropriate controls, replication, and sample sizes. The conclusions must be drawn appropriately based on the data presented.

Reviewer #3: Yes

Reviewer #4: Partly

Reviewer #5: Yes

3. Has the statistical analysis been performed appropriately and rigorously?

Reviewer #3: I Don't Know

Reviewer #4: Yes

Reviewer #5: Yes

4. Have the authors made all data underlying the findings in their manuscript fully available?

The [PLOS Data policy](http://www.plosone.org/static/policies.action#sharing) requires authors to make all data underlying the findings described in their manuscript fully available without restriction, with rare exception (please refer to the Data Availability Statement in the manuscript PDF file). The data should be provided as part of the manuscript or its supporting information, or deposited to a public repository. For example, in addition to summary statistics, the data points behind means, medians and variance measures should be available. If there are restrictions on publicly sharing data—e.g. participant privacy or use of data from a third party—those must be specified.

Reviewer #3: Yes

Reviewer #4: Yes

Reviewer #5: Yes

5. Is the manuscript presented in an intelligible fashion and written in standard English?

PLOS ONE does not copyedit accepted manuscripts, so the language in submitted articles must be clear, correct, and unambiguous. Any typographical or grammatical errors should be corrected at revision, so please note any specific errors here.

Reviewer #3: Yes

Reviewer #4: Yes

Reviewer #5: Yes

6. Review Comments to the Author

Please use the space provided to explain your answers to the questions above. You may also include additional comments for the author, including concerns about dual publication, research ethics, or publication ethics. (Please upload your review as an attachment if it exceeds 20,000 characters)

**Reviewer #3**: The findings in the study highlight the complexity of developing RIs. the results suggest that racial/ethnic subpopulations have unique distributions in laboratory tests, accordingly further work is needed to establish of racial/ethnic-specific RIs that may have significant clinical and public health implication for more accurate disease diagnosis and appropriate treatment to improve quality of patient care. ** the author should give a more emphasizes in the recommendation
I hope the study may draws attention to a possible revision of the WHO reference intervals in regard

I think the manuscript is going to be useful like that
no further comments

**Our response →** We appreciate a great deal for reviewing our manuscript. We are pleased to have your encouraging message to our work. Yes, it was a very complex and demanding work to determine RIs appropriately for many heterogenous analytes simultaneously. We highlighted the point in the end of Introduction, in the beginning of the Discussion, and in the Conclusion. We hope the table we made for international comparison of recently reported RIs is of relevance to the readers.

**Reviewer #4**: Ruzhanskaya et al present complete blood count and iron related marker reference intervals (RIs)from subjectively healthy Russians recruited as part of the global study on reference intervals carried out by the Interbational Federation of Clinical Chemistry. They use both parametric and non-parametric approaches to derive RIs after secondarily excluding individuals based on different criteria including latent abnormal value exclusion. Overall, this is a well written paper presenting useful evidence based information on CBC reference intervals for adoption in Russian.

**Our response →**We are very grateful for your time and efforts in reviewing our manuscript. We are pleased to have your positive over-all evaluation to our work. Besides, we appreciate a great deal for providing us with invaluable comments to improve the scientific quality of our manuscript. We addressed the issues raised and corrected one by one as shown below.

**Major concerns**
1. The statistical approach is subjective and not applied in a consistent manner that would make the results reproducible. For example, the basis for excluding ‘outliers’ appears to be arbitrary since the RI width for both upper and lower limits was increased by 3% for no other reason other than to reduce number of reference individuals excluded.

**Our response →** We appreciate for pointing out the importance of reproducibility in our methodologies. We admit the problem of somewhat arbitrary nature of our condition setting to perform the latent abnormal value exclusion (LAVE) method. In fact, there are many options to choose from: i.e., (1) what analytes for use as reference tests (RTs: 5, 7, or more), (2) how many times to adopt for iterative adjustment of RIs (i.e., usually 6 times but can be 5 or 7~), (3) when to adjust the RIs of RTs (i.e., no adjustment of the RI, 3% or 5% extension on both-ends of the RI), etc. Therefore, these adjustments have been done in a trial-and-error fashion over the past 25 years, and we obtained empirical thresholds of 6-time repetitive adjustment of RIs and 0~5% extension of RIs for RTs were most suitable in terms of “how many data remain after LAVE procedure” and “how-much narrowing of the RI occurs”.

In any case, we added the following point in the Limitation as to the need for optimizing the performance of the LAVE method by adjusting the settings.

“*Another limitation of this study is somewhat arbitrary nature of setting conditions for secondary exclusion using the LAVE method. In fact, there can be many options to attain optimal LAVE effect without much data-size reduction, which depend on how many reference tests to set up and how much to expand the RI limits in judging test result abnormality. Although we adopted 3% expansion on both-end of the RI from our past trial and error experiences, other settings can lead to similar results*.”

2. Exclusion of individuals was partly based on manufacturer recommended cut-offs for ferritin (notably higher than WHO recommended cut-offs) despite not knowing whether they are appropriate for the Russian population. It is possible that many individuals were inappropriately excluded as being iron deficient. As observed, the derived RIs for ferritin in women had a lower limit that was lower than the manufacturer recommend cut-off.

**Our response →** We understand the reviewer’s concern over the use of unconfirmed ferritin (Ferr) RI to select women with iron-deficiency anemia (IDA). Unfortunately, our writing seems to have aroused some confusion. In our final determination of RIs, we did not apply manufacturer’s Ferr cutoff value in excluding individual with latent anemia. Rather, we just needed to choose a subgroup of women without IDA by the levels of Ferr and iron in order to demonstrate the effectiveness of the LAVE method in excluding the influence of IDA (please refer to **Fig. 3**). Therefore, it is certainly unlikely that “many individuals were inappropriately excluded as being iron deficient”.

In any case, as for the manufacturer recommended cutoff for female ferritin, we understand that it was fully aligned to those provided in Russian guidelines of National Hematology Laboratory Society (NHLS; 11 mcg/L), which was close to WHO cutoff (15 mcg/L).

Regarding our finding that “the derived RIs for ferritin in women had a lower limit that was lower than the manufacturer recommend cut-off”. We must acknowledge it as a fact for Russian women despite our best efforts of reducing the influence of IDA by using the LAVE methods. **We recognized this point as** **we wrote about it in the Discussion as pasted below**:

“*In Russian females we observed a slightly reduced LL of Hb in comparison to the AGA and RNHS guidelines (114 vs. 120 g/L) [36,37], meanwhile significantly lower ferritin LL was observed in both age groups <45 and ≥45 years (5.5 and 5.3 vs. 45 and 11 µg/L, respectively) [36-38]. Even a lower level of ferritin was reported in the Turkish and Japanese studies (4.3 and 3 µg/L, respectively) [5,6]. Low levels of ferritin in age group <45 years are associated with reduced iron storage due to menstrual bleeding, pregnancy or latent anemia [8]. Bawua et. al. showed age-related increase in ferritin and decrease in TFR, which was confirmed in this study. However, LL for ferritin in Russian females for the age group >45 is still very low (5.3 µg/L)*.”

3. It is indicated that an SDR>0.4 was part of the criteria used to determine the need for partitioning. However, partitioning based on sex was done for platelets despite having an SDRsex that was less than 0.4.

**Our response →** We applied two tiers of criteria in judging the need for partitioning reference values (RVs): The first by using (1) SD ratio (SDR): between-group SD divided by within-group SD, then (2) by using bias ratio (BR) at lower and upper limits (LL, UL) of the RI

Accordingly, there can be situation where SDRsex is less than the threshold (<0.40), but actual BR at UL exceeds its threshold of 0.57. Such discrepancy was typically observed in RVs of platelets and Eos as picked-up below.

4. Given the complexity of the analyses, it would be beneficial to have a clear schema that summarizes the logic behind partitioning of the RIs. For example, for the bias ratio, was 0.57 or 0.375 used as the criteria? It is not clear the basis for multiplying 0.375 with the square root of two. What does the square root of two represent.

**Our response →** We are sorry about the complicate nature of defining threshold for judging the magnitude of “between-group” differences. The important distinction between SDR and BR is that **SD is calculated as standard deviation** ($\sqrt{\sum_{i=1}^{n} {(x_{i}-\bar{x})}^{2}/(n-1)}$ while **BR is a calculated as a difference of two values** |$\bar{x}_{1}-\bar{x}_{2}|$or bias.

Therefore, to set the threshold of BR aligned to the threshold of SDR=0.4 (for judging between-sex difference), we multiplied SDR=0.4 by $\sqrt{2}$ to obtain its threshold of 0.4×$\sqrt{2}$=0.57: i.e., absolute difference (bias) of two values (x_1_ and x_2_), $|x_{1}-x_{2}|$ is $\sqrt{2}$times larger than SD of the two values

$\sqrt{\left\{ \left( x_{1}-\bar{x} \right)^{2}+\left( x_{2}-\bar{x} \right)^{2} \right\}/(2-1)}$ . **Please refer to the proof below in brown texts for cases of SDR of two groups with n_1_ and n_2_**.

Another complicate thing was our use of two different thresholds (1) for judging between-sex differences and (2) for judging the effect of LAVE method. The former (SDR=0.4 or BR=0.57) was inferred from conventional clinical practice of setting sex-specific, while for the latter, the threshold of BR=0.375 was decided in analogy to the conventional allowable analytical bias.

**In the revised manuscript, we added the following red-texts to clarify our scheme of using different threshold**.

**“4.2) Criteria for partitioning RVs**

The standard deviation ratio (SDR) was used as a criterion for assessing the need of partitioning RVs by sex and age. To calculate SDR, pure components of between-sex SD (SD_sex_) and between-age SD (SD_age_) were first computed by two-level nested ANOVA and within-group SD (approximately 1/4 the width of RI, or SD_RI_). The SDRs for sex or age (SDR_sex_, SDR_age_) were calculated by taking respective ratios of SD_sex_ and SD_age_ to SD_RI_. Since SDR_age_ often differs between sexes, it was also calculated by one-way ANOVA after partitioning RVs by male and female, as SDR_ageM_, SDR_ageF_. The need for partition of RVs was considered by setting SDR≥0.4 as a primary guide: i.e., the threshold of 0.4 was inferred from conventional clinical practice of setting sex-specific RIs [14].

However, SDR can be too sensitive, when the width of RI constituting the denominator of SDR is narrow. Conversely, SDR may be insensitive, when between-subgroup differences occur only at the periphery of distribution (LL or UL) — SDR represents between-subgroup differences at the center of the distributions. Therefore, we additionally considered actual difference (bias) at LL or UL as “bias ratio” (BR) using the following formula, illustrating a case of gender difference:

BR_LL_ = $\frac{|{LL}_{M}－{LL}_{F}|}{({UL}_{MF}－{LL}_{MF})/3.92}$, BR_UL_ = $\frac{|{UL}_{M}－{UL}_{F}|}{({UL}_{MF}－{LL}_{MF})/3.92}$

where subscript M, F, and MF represent male, female, and male+female, respectively. The denominator of each formula represents the standard deviation (SD_RI_) comprising the RI, the width of which corresponds to 3.92 times SD_RI_. To make the threshold of BR aligned to SDR with the common denominator (SD_RI_), we multiplied the threshold of SDR=0.4 by $\sqrt{2}$ to obtain BR’s threshold of 0.57 (i.e., absolute difference (bias) of two values (x_1_ and x_2_), or |x_1_−x_2_|, is $\sqrt{2}$ times larger than SD of the two values).

Whereas, when BR was used for judging the effectiveness of the LAVE method, its threshold was set according to the conventional specification of allowable analytical bias (=difference) of a minimum level: 0.375 ×$\sqrt{{SD}_{G}^{2}+{SD}_{I}^{2}}$ (= SD_RI_) [17]. Hence 0.375 was set as the threshold for BR in contrast to the above-mentioned threshold of BR for between-sex bias, which was inferred from conventional clinical practice.

--------------------

**Appendix: Relationship of between-group SD vs. between-group deference in means** ${\bar{\boldsymbol{x}}}_{\boldsymbol{1}}\boldsymbol{-}{\bar{\boldsymbol{x}}}_{\boldsymbol{2}}$

In considering a threshold of SDR for a case of two groups, it is convenient **to convert between-group SD (the numerator of SDR) to between-group deference of means (**${\bar{\boldsymbol{x}}}_{\boldsymbol{1}}\boldsymbol{-}{\bar{\boldsymbol{x}}}_{\boldsymbol{2}}$**)** because analytical bias is conventionally expressed in terms of a deference in means of two sets of values. The relationship between the SD and $\bar{x}_{1}-\bar{x}_{2}$ can be derived as below by setting the number of groups as k=2, sample sizes as n_1_ and n_2_, SDs as s_1_, s_2_.

From definition of SD, $s_{1}=\sqrt{\frac{\sum_{i=1}^{n_{1}} {(x_{1i}-\bar{x}_{1})}^{2}}{n_{1}-1}}$ , $s_{2}=\sqrt{\frac{\sum_{i=1}^{n_{1}} {(x_{2i}-\bar{x}_{2})}^{2}}{n_{2}-1}}$ . The total sample size $N=n_{1}+n_{2}$.

$s_{A}^{2}=\frac{n_{1}{(\bar{x}_{1}-\overset{̿}{x})}^{2}+n_{2}{(\bar{x}_{2}-\overset{̿}{x})}^{2}}{2-1}=\frac{n_{1}\cdot n_{2}}{n_{1}+n_{2}}{(\bar{x}_{1}-\bar{x}_{2})}^{2}$ *(E1) ⸱⸱⸱⸱⸱* where grand mean $\overset{̿}{x}=\frac{n_{1}\bar{x}_{1}+n_{2}\bar{x}_{2}}{n_{1}+n_{2}}$

$s_{E}^{2}=\frac{{(n}_{1}-1)s_{1}^{2}+{(n}_{2}-1)s_{2}^{2}}{n_{1}+n_{2}-2}$	*(E2)*

$n_{0}=N-\frac{n_{1}^{2}+n_{2}^{2}}{N}$	*(E5)*

$\hat{s}_{A}^{2}=\frac{s_{A}^{2}-s_{E}^{2}}{n_{0}}=\frac{\frac{n_{1}\cdot n_{2}}{N}{(\bar{x}_{1}-\bar{x}_{2})}^{2}-s_{E}^{2}}{N-\frac{n_{1}^{2}+n_{2}^{2}}{N}}=\frac{n_{1}\cdot n_{2}\left( \bar{x}_{1}-\bar{x}_{2} \right)^{2}-N\cdot s_{E}^{2}}{N^{2}-(n_{1}^{2}+n_{2}^{2})}=\frac{1}{2} \left[ {(\bar{x}_{1}-\bar{x}_{2})}^{2}-\frac{n_{1}+n_{2}}{n_{1}\cdot n_{2}}s_{E}^{2} \right]$	*(E4)*

Assuming $n_{1}=n_{2}=n$, the second term in (E4) turns to ${2s}_{E}^{2}/n$.

When n is large (say n>100), the second term is regarded as near zero. Hence, the (E4') reduces to

$\hat{s}_{A}^{2}\approx\frac{1}{2}{(\bar{x}_{1}-\bar{x}_{2})}^{2}$	*(E4’)*

This implies that SD for SV factor of 'A' for a two category case, $\hat{s}_{A}$ can be calculated as $1/\sqrt{2}$ of between-group deference in means or $|\bar{x}_{1}-\bar{x}_{2}|$.

$\boldsymbol{SDR}_{A}=\frac{{\hat{\boldsymbol{s}}}_{\boldsymbol{A}}}{\boldsymbol{s}_{\boldsymbol{E}}}\approx\frac{{\boldsymbol{|}\bar{\boldsymbol{x}}}_{\boldsymbol{1}}\boldsymbol{-}{\bar{\boldsymbol{x}}}_{\boldsymbol{2}}\boldsymbol{|/}\sqrt{\boldsymbol{2}}}{\boldsymbol{s}_{\boldsymbol{E}}}$ *(E6)*

--------------------

**Minor concerns**
See in-text comments for minor comments.

**Our response →** We requested the editorial office for the “in-text comments”, but there was no response.

**Reviewer #5**: The article plays an important role in standardizing reference values for a population with little ethnic diversity and demonstrating variations and correlations between reference values, influenced by sex, age and BMI. However, I suggest that the authors provide more details about the secondary patient exclusion criteria (information about health status acquired in the questionnaire). It was not clear how the exclusion and inclusion, even presenting a reference to Cohen, required more details.

**Our response →**We are very grateful to your time and efforts for reviewing our manuscript and providing us with comments to make our manuscript clearer for the readers to follow.

**Regarding the inclusion and exclusion criteria, we wrote it clear in the beginning of the Materials and Methods as pasted below**:

“**Materials and Methods**

**1) The study protocol and recruitment**

Healthy volunteers were recruited with the objectives of determining RIs for hematological and related parameters and exploring the source of variations of each analyte for better derivation and clinical interpretation of test results. The study protocol was prepared in harmonization to that provided by the IFCC, C-RIDL [3]. The inclusion and exclusion criteria adopted in the recruitment were as follows:

**Inclusion criteria**

The participants were feeling well, between the ages of 18 and 65 and ideally, they were not taking any medication or supplements. However, if they were taking medication, doses and frequency were recorded accordingly.

**Exclusion criteria**

The participants were excluded if any of the following were applicable: if he or she was diabetic and on oral therapy or insulin, had a history of chronic liver or kidney disease, had results from their blood samples that clearly point to a severe disease, had been a hospital in-patient or been subjectively seriously ill during the previous 4 weeks of participation, donated blood in the previous 3 months, if they were a known carrier of HBV, HCV, or HIV, if she was pregnant or within one year after childbirth and if they had participated in another research study involving an investigational product in the past 12 weeks.

*This rather lenient criteria clearly entailed the need for secondary exclusion after measurements based on the pattern of the actual test results as described below. The health-status information acquired from the questionnaire (i.e., BMI, habits of regular exercise, drinking, or smoking, etc. [3]) was also used to consider for the secondary exclusion.*”

Regarding the ways for secondary exclusion, we did it meticulously because of its necessity ***as described above in the italic part of descriptions***. The most important scheme of secondary exclusion was (1) to exclude highly prevalent latent anemia using the latent abnormal values exclusion method, and (2) to exclude obese individuals for determining RIs for analytes with significant association of their values with BMI. As for the effect of smoking or allergy, we found their effects were negligible by the multiple regression analysis. Since these descriptions are already in the current manuscript, we hope it is not necessary to add more detailed descriptions.

As for the Cohen’s criteria, it is customary to judge the magnitude of observed correlations (partial correlation coefficients) in terms of “effect size”, rather than judging the magnitude by P value, which depends on data size and may not represent practically important degree of “correlation”.

Therefore, we modified its description in Statistical methods as follows:

“*Since statistical testing of r_p_ is too sensitive with large data size, we interpreted its practical significance (“effect size”) in reference to the Cohen’s guide [16] as small 0.1< |r_p_| <0.3; medium 0.3≤ |r_p_| <0.5, large |r_p_| ≤ 0.5. Hence, we regarded* *|0.3| ≤r_p_ as significant when considering the influence of a given factor on the RVs.*”

----------------

We hope our answer to the Reviewer-5’s comments are satisfactory also to answer to the following comments kindly offered by the Academic Editor:

==============================
ACADEMIC EDITOR:

Dr. Ichihara

This is an interesting manuscript that show relevant information on reference values of some biochemical parameters. I recommend to evaluate the suggestion of the reviewer 5 "I suggest that the authors provide more details about the secondary patient exclusion criteria (information about health status acquired in the questionnaire). It was not clear how the exclusion and inclusion, even presenting a reference to Cohen, required more details". Such information is relevant to strengten your results. Our decision is justified on PLOS ONE’s [publication criteria](https://journals.plos.org/plosone/s/criteria-for-publication) and not, for example, on novelty or perceived impact.
==============================

7. PLOS authors have the option to publish the peer review history of their article ([what does this mean?](https://journals.plos.org/plosone/s/editorial-and-peer-review-process#loc-peer-review-history)). If published, this will include your full peer review and any attached files.


If you choose “no”, your identity will remain anonymous but your review may still be made public.


**Do you want your identity to be public for this peer review?** For information about this choice, including consent withdrawal, please see our [Privacy Policy](https://www.plos.org/privacy-policy).

Reviewer #3: **Yes:** ANISA H. ALBITI

Reviewer #4: No

Reviewer #5: No

[NOTE: If reviewer comments were submitted as an attachment file, they will be attached to this email and accessible via the submission site. Please log into your account, locate the manuscript record, and check for the action link "View Attachments". If this link does not appear, there are no attachment files.]

While revising your submission, please upload your figure files to the Preflight Analysis and Conversion Engine (PACE) digital diagnostic tool, <https://pacev2.apexcovantage.com/>. PACE helps ensure that figures meet PLOS requirements. To use PACE, you must first register as a user. Registration is free. Then, login and navigate to the UPLOAD tab, where you will find detailed instructions on how to use the tool. If you encounter any issues or have any questions when using PACE, please email PLOS at [figures@plos.org](mailto:figures@plos.org). Please note that Supporting Information files do not need this step.
